# Design, synthesis and evaluation of novel 1,2,4-triazole derivatives as promising anticancer agents

**DOI:** 10.1186/s13065-022-00887-x

**Published:** 2022-11-12

**Authors:** Leila Emami, Sara Sadeghian, Ayyub Mojaddami, Soghra khabnadideh, Amirhossein Sakhteman, Hossein Sadeghpour, Zeinab Faghih, Masood Fereidoonnezhad, Zahra Rezaei

**Affiliations:** 1 Pharmaceutical Sciences Research Center, School of Pharmacy, Shiraz University of Medical Sciences, P.O. Box: 71345-1798, Shiraz, Iran; 2 Department of Medicinal Chemistry, School of Pharmacy, Shiraz University of Medical Sciences, Shiraz, Iran; 3 Department of Medicinal Chemistry, School of Pharmacy, Ahvaz Jundishapur University of Medical Sciences, Ahvaz, Iran; 4 Toxicology Research Center, Medical Basic Sciences Research Institute, Ahvaz Jundishapur University of Medical Sciences, Ahvaz, Iran

**Keywords:** 1,2,4-Triazole, Anticancer, MTT assay, Molecular docking, ADME

## Abstract

Herein, we reported the synthesis of nineteen novel 1,2,4-triazole derivatives including 1,3-diphenyl-2-(1H-1,2,4-triazol-1-yl) propan-1-ones (***7a-e***), 1-(1,3-diphenylpropan-2-yl)-1*H*-1,2,4-triazole (***8a-c***) and 1,4-diphenyl-2-(1H-1,2,4-triazol-1-yl) butane-1,4-diones (***10a-k***). The structures of these derivatives were confirmed by spectroscopic techniques like IR, ^1^H-NMR, Mass spectroscopy and Elemental analysis. The cytotoxic activities of the synthesized compounds were evaluated against three human cancer cell lines including MCF-7, Hela and A549 using MTT assay. Compounds ***7d***, ***7e***, ***10a*** and ***10d*** showed a promising cytotoxic activity lower than 12 μM against Hela cell line. The safety of these compounds was also, evaluated on MRC-5 as a normal cell line and relieved that most of the synthesized compounds have proper selectivity against normal and cytotoxic cancerous cell lines. Finally, molecular docking studies were also, done to understand the mechanism and binding modes of these derivatives in the binding pocket of aromatase enzyme as a possible target.

**Supplementary Information:**

The online version contains supplementary material available at 10.1186/s13065-022-00887-x.

## Introduction

Cancer is characterized by the uncontrolled growth and proliferation of abnormal cells and is the second leading cause of morbidity and mortality in the world [1, 2]. According to the global cancer statistics, 9.6 million deaths and also, more than 18 million new cancer occurred in 2018 [3]. It is expected that the cancer mortality rate will rise dramatically in the future [3].

Various internal and external factors cause abnormal cell proliferation, leading to development of various cancers, including genetics, viruses, drugs, diet and smoking [4, 5]. Currently, three strategies including chemotherapy, radiotherapy, and surgery are used for the treatment of cancer. Chemotherapy is the most common treatment for cancer disease, in which various chemotherapeutic agents are utilized to kill the cancer cells with minimum harmful effect on normal cells [4, 6]. However, drug resistance, non-selectivity and toxicity of many anticancer drugs have limited their clinical uses [1, 7]. Hence, the discovery and development of more effective and potent anticancer agents is one of the most clinical challenges in modern medicinal chemistry [8].

**Fig. 1:**
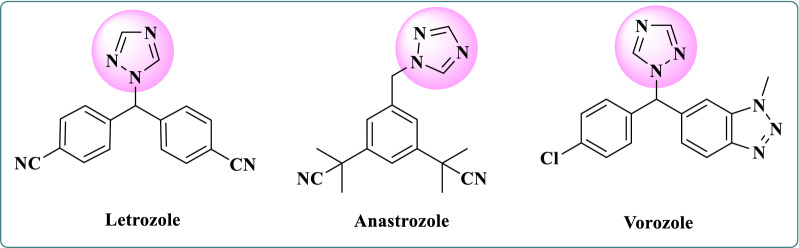
Chemical structures of 1,2,4-triazole-based drugs

Heterocyclic compounds containing nitrogen atoms, especially heterocyclic rings with three nitrogen atoms, like 1,2,4-triazole ring, are one of the most important active pharmaceutical scaffolds. These scaffolds are able to form hydrogen bonds with different targets, which leads to the improvement of pharmacokinetics, pharmacological, and toxicological properties of compounds [2–4, 9]. Among heterocyclic compounds, 1,2,4-triazole derivatives have attracted much attention because of their various biological activities such as antiviral [10], antibacterial [11], antifungal [12, 13], anti-tubercular [14–16], immunosuppressant [17], antihypertensive [18], anti-inflammatory [19, 20], anticonvulsant [21, 22], analgesic [23], hypoglycemic [24], antidepressant [25, 26] and anticancer [9, 27, 28] activities. Currently, Letrozole, Anastrozole, and Vorozole which are 1,2,4-triazole-based drugs, are widely used in the treatment of estrogen-dependent breast cancer [29, 30] (Fig. 1).

**Fig. 2:**
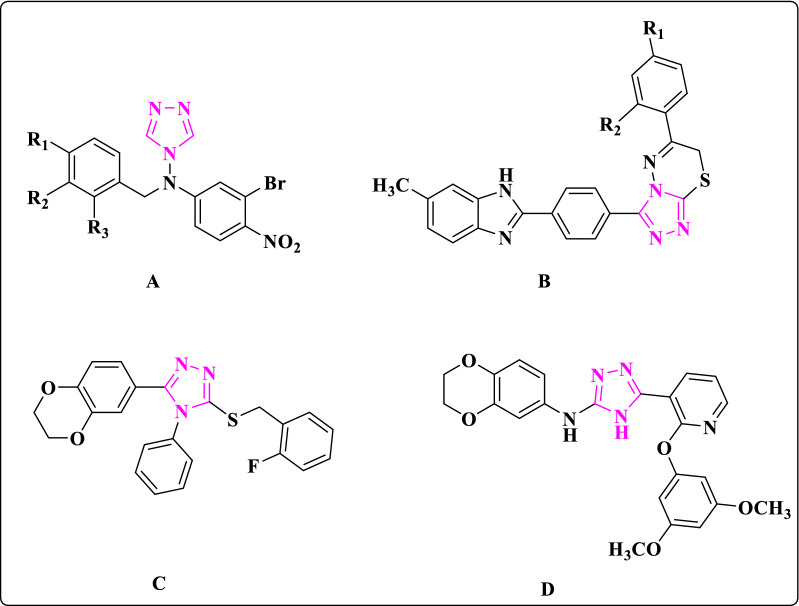
Structure of 1,2,4-triazole derivatives with anticancer activity

Some Clotrimazole derivatives have been reported as antifungal agents [2, 9, 31–33]. Song et al. synthesized a series of 4-N-nitrophenyl substituted amino-4H-1,2,4-triazole derivatives as promising aromatase inhibitors (Fig. 2, A) [34]. Moreover, Cevik et al. explored a new set of benzimidazole-triazolothiadiazine hybrids with potent aromatase inhibitory activities (Fig. 2, B) [35]. Hou et al. reported a series of 1,2,4-triazole derivatives with potent inhibitory activity against HepG2 cancer cell line (Fig. 2, C) [36]. In addition, X. Ouyang et al. showed that a set of 1,2,4-triazole derivatives, completely inhibited the tubulin polymerization by inducing cell cycle arrest at the G2/M phase of A431 cell line (Fig. 2, D) [37].

In the present study, three series of 1,2,4-triazole derivatives including 1,3-diphenyl-2-(1H-1,2,4-triazol-1-yl) propan-1-ones (***7a-e***), 1-(1,3-diphenylpropan-2-yl)-1*H*-1,2,4-triazole (***8a-c***) and 1,4-diphenyl-2-(1H-1,2,4-triazol-1-yl) butane-1,4-diones (***10a-k***) derivatives were designed, synthesized and evaluated for their anticancer activity against three human cancer cell lines (MCF-7, Hela and A549). The cytotoxic activity of all the synthesized compounds were assessed using the standard 3-(4,5-dimethylthiazol-yl)-2,5-diphenyl-tetrazolium bromide (MTT) assay. Furthermore, molecular docking study was carried out to find the possible interaction mode of these derivatives in the active site of aromatase enzyme as possible target.

## Results and discussion

### Design

**Fig. 3:**
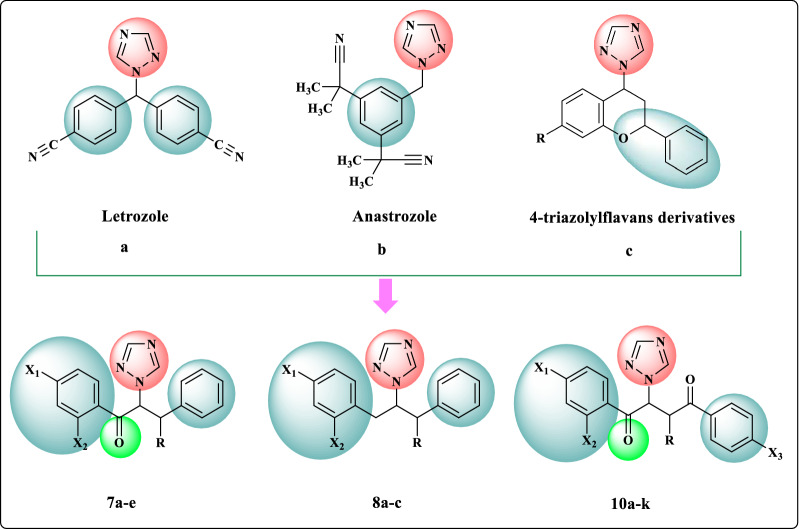
Design of new 1,2,4-triazole derivatives

The target 1,2,4-triazole derivatives were designed based on the chemical structures of Letrozole (**a**), Anastrozole (**b**) and 4-triazolylflavans (**c**) which act as aromatase inhibitors. Aromatase is a member of the cytochrome P450 superfamily that catalyzes the estrogen biosynthesis and can be considered as a therapeutic target due to its overexpression in breast cancer. Anastrozole and Letrozole are potent aromatase inhibitors that use in the treatment of ER‐positive breast cancer. In addition, it has been previously reported that 4-triazolylflavans derivatives exhibited aromatase inhibitory effect [38, 39]. In these aromatase inhibitors, nitrogen atoms of 1,2,4-triazole ring bind to the iron in the heme moiety of CYP-450 and phenyl moieties have a key interaction in the active site of enzyme. Furthermore, carbonyl group is incorporated in the designed structures due to its ability to form hydrogen bonds. Therefore, 1,2,4-triazole moiety, phenyl rings and carbonyl groups were incorporated in the designed scaffolds (Fig. 3).

### Chemistry

**Fig. 4:**
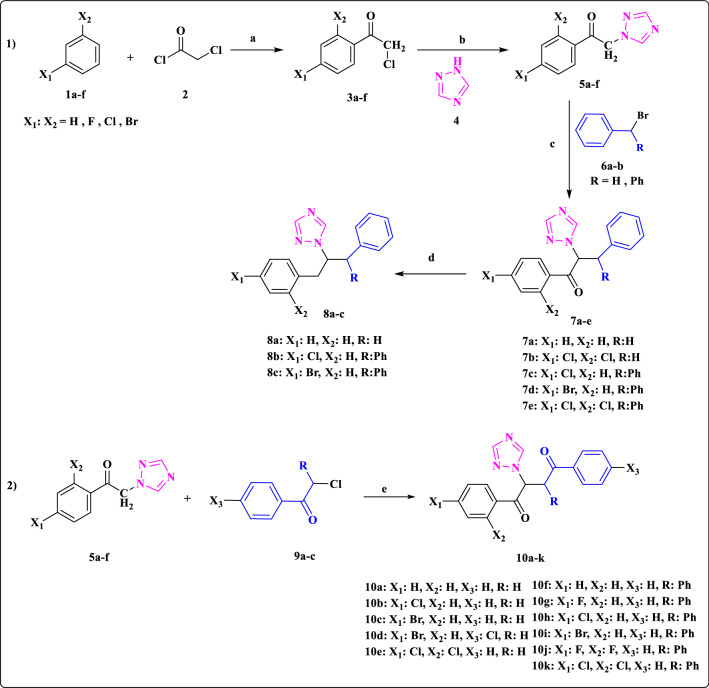
Synthesis of target compounds ***7a-e*, *8a-c*** and ***10a-k***. Reagents and conditions: a) AlCl_3_, dichloromethane, r.t., 24 h, b) NaHCO_3_, toluene, reflux, 20 h, c) NaH, CH_3_CN, reflux, 24 h, d) N_2_H_4_.H_2_O, KOH, Ethylene glycol,170 ˚C, 4 h, e) NaH, CH_3_CN, reflux, 24 h

The synthesis of the desired compounds (***7a-e*, *8a-c*, *10a-k***) was carried out according to the synthetic pathway illustrated in Fig. 4. The phenacyl chloride derivatives ***3a-f*** were prepared in high yield through Fridel Crafts acylation of mono or di-substituted benzene (***1a-f***) with chloroacetyl chloride (**2**) using aluminum trichloride (AlCl_3_) as a strong Lewis acid catalyst [40, 41]. In the second step, intermediates ***5a-f*** were synthesized from the reaction of intermediates ***3a-f*** with 1,2,4-triazole (**4**) in the presence of sodium bicarbonate (NaHCO_3_). Compounds ***7a-e*** were synthesized by treating benzyl bromide (***6a***) or benzhydryl bromide (***6b***) with intermediates ***5a-f*** in the presence of NaH as a strong base catalyst in acetonitrile. Subsequently, Huang Minlon reduction of compounds ***7a*** and ***7c-d*** in the presence of N_2_H_4_.H_2_O and KOH yielded compounds ***8a-c*** [42]. Compounds ***10a-k*** were prepared from the reaction of 2-chloro-1,2-diphenylethanone (desyl chloride) or 2-chloro-1-phenylethanone derivatives with key intermediates ***5a-f*** utilizing NaH as base and in acetonitrile as solvent. The structures of the synthesized compounds were confirmed through ^1^H-NMR, Mass and IR techniques. In the IR spectra of compounds ***7a-e*,** a signal for C = O group was observed at 1690–1723 cm^−1^, while this signal was removed for compounds ***8a-c***. The IR absorption spectra of ***10a-k*** were characterized the presence of two signals for C = O groups at 1650–1712 cm^−1^. ^1^H-NMR spectrum of compounds ***7a-e*** showed two singlet peaks at 8.12–8.33 and 7.77–7.91 ppm assigned to the 1,2,4-triazole ring. In addition, in compounds ***7a*** and ***7b*,** the CH proton was observed at 6.17–6.24 ppm as a doublet of doublet peak and in compounds ***7c-e*** the CH proton was observed at 6.73–6.79 ppm as a doublet peak. ^1^H-NMR spectrum of compounds ***8a-c*** showed two singlet peaks at 8.17–8.29 ppm and 7.78–7.85 ppm assigned to the 1,2,4-triazole ring and a multiplet peak at 4.59–4.67 ppm assigned to the CH proton. ^1^H-NMR spectrum of compounds ***10a-k*** showed two singlet peaks at 7.97–8.35 ppm and 7.79–8.03 ppm assigned to the 1,2,4-triazole ring. Furthermore, in compounds ***10a-e*** with R = H, the CH proton appeared at 6.52–6.69 ppm as a triplet peak whereas in compounds ***10f-k*** with R = Ph, the CH proton appeared at 6.45–6.81 ppm as a doublet peak. All the analytical data were documented in the Additional file 1: Data.

### Evaluation of anticancer activity

**Fig. 5:**
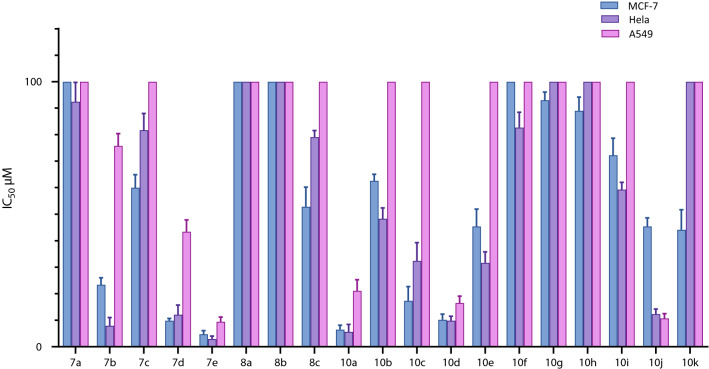
Cytotoxic effect of compounds ***7a-e, 8a-c*** and ***10a-k*** on MCF-7, Hela and A549 cell lines Taken to gather, regarding the cytotoxic evaluations on ***7a****-****e*** and ***8a****-****c*** derivatives, it can be realizing that ***7e*** was the most potent derivative against all three tested cell lines. The structure activity relationship disclosed that electronegative substitution such as Cl and Br at para position of phenyl ring (X_1_) and also, the presence of phenyl ring at R position could increase the inhibitory activity significantly in a ***7a-e*** series. Also, the presence of one-carbonyl group showed necessary for pharmacological effect. In addition, propane-1-yl-derivatives (***8a****-****c***) had least effect on cytotoxic activity. In the case of ***10a****-****k***, replacement of H with Ph moiety at R position led to decreased cytotoxic activity (***10f****-****k***) and also, no substituted analogue (***10a***) had favorable pharmacological effect on MCF-7 and Hela cell lines. The cytotoxicity of all synthesized compounds were shown in Table 1.

All of the synthetic compounds (***7a****-****e****, ****8a****-****c*** and ***10a****-****k***) were screened for their in vitro cytotoxic effects against three human cancerous cell lines (MCF-7, Hela and A549) using MTT assay [43–45]. The biological results were expressed in terms of IC_50_ (Table 1). Generally, the best anticancer effect was seen on breast (MCF-7) and cervical (Hela) cell lines and less on lung cancer (A549) cell line. In this assay, *cis platin* was used as positive control [46–48]. As shown in Table 1, some of the compounds such as ***7b***, ***7d-7e***, ***10a*** and ***10c-d*** showed better antiproliferative activity compared to *cis platin* in all studied cancer cell lines. Assessments on propane-1-one derivatives (***7a****-****e***), compound ***7e*** bearing X_1_:X_2_ = Cl, R = Ph showed significant activity with IC_50_ = 4.7, 2.9 and 9.4 μM against MCF-7, Hela and A549 cell lines, respectively, followed by compound ***7d*** possessing X_1_ = Br, X_2_ = H, R = Ph with IC_50_ = 9.8, 12.1 and 43.4 μM. The obtained results indicated that the absence of electronegative groups at X position and also, the absence of phenyl ring at R position in analogue ***7a*** (unsubstituted one), dramatically decreased the cytotoxic effects against all studied cancer cell lines. On the other hand, propane-1-yl-derivatives (***8a****-****c***), showed less cytotoxicity effect compared to other studied compounds (***7a****-****e*** and ***10a****-****k***), which can be attributed to the reduction of carbonyl group at position 1 of propane chain. Further, a comparison of the cytotoxicity of compounds ***7a-e*** on Hela cell line showed that the anticancer activities of different substitutions on phenyl ring followed the order 2,4-di-Cl > 4-Br > 4-Cl > H, and interestingly, the relative order of the substitution effect on cytotoxicity for compounds ***8a****-****c*** was 4-Br > 4-Cl > H which was in line with above-mentioned results. In the case of butane-1,4-dione derivatives (***10a****-****k***), compound ***10a*** was found to have promising anticancer activity with IC_50_ = 6.43, 5.6 and 21.1 μM against MCF-7, Hela and A549 cell lines, respectively. The substitution of Br and Cl at X_1_ and X_3_ position produced appropriate derivative (***10d***) with IC_50_ values of 10.2, 9.8 and 16.5 μM. Besides, the cytotoxic activity of compounds ***10a****-****e*** on Hela cell line showed that the anticancer activities of different substitutions on the phenyl rings followed the order of H > 4Br > 2,4-di-Cl > 4-Cl. The incorporation of phenyl group at R position decreased cytotoxic activity. Among these compounds, ***10j*** bearing 2,4-diflouro group at the phenyl moiety demonstrated adequate cytotoxic effect. The safety of these compounds was also, evaluated on MRC-5 as normal cell line. The resulted indicated that most of the synthesized compounds have proper selectivity against cancer cell lines (Fig. 5).

**Table 1:** Cytotoxicity of the synthesized compounds against MCF-7, Hela and A549 cell lines [IC_50_ (μM)]

Compound	X_1_	X_2_	X_3_	Y	R	IC_50_ (μM ± SEM)			
						MCF-7	Hela	A549	MRC-5
***7a***	H	H	–	C = O	H	154.2 ± 5.8	92.4 ± 7.3	149.6 ± 5.3	> 300
***7b***	Cl	Cl	–	C = O	H	23.4 ± 2.6	7.9 ± 3.1	75.8 ± 4.6	72.6 ± 3.4
***7c***	Cl	H	–	C = O	C_6_H_5_	60.0 ± 4.9	81.7 ± 6.3	177.3 ± 5.9	201.2 ± 9.5
***7d***	Br	H	–	C = O	C_6_H_5_	9.8 ± 0.9	12.1 ± 3.6	43.4 ± 4.5	35.6 ± 2.7
***7e***	Cl	Cl	–	C = O	C_6_H_5_	4.7 ± 1.4	2.9 ± 1.1	9.4 ± 1.8	27.8 ± 3.7
***8a***	H	H	–	CH_2_	H	158.3 ± 3.5	141.5 ± 2.1	127.3 ± 4.9	279.1 ± 2.5
***8b***	Cl	H	–	CH_2_	C_6_H_5_	178.5 ± 2.5	198.3 ± 5.5	124.1 ± 3.4	279.1 ± 2.8
***8c***	Br	H	–	CH_2_	C_6_H_5_	52.8 ± 7.4	79.1 ± 2.5	104.5 ± 7.3	104.5 ± 7.3
***10a***	H	H	H	–	H	6.4 ± 1.7	5.6 ± 2.8	21.1 ± 4.2	21.7 ± 1.5
***10b***	Cl	H	H	–	H	62.6 ± 2.5	48.3 ± 4.1	110.5 ± 1.3	259.3 ± 8.3
***10c***	Br	H	H	–	H	17.3 ± 5.4	32.4 ± 6.9	103.7 ± 1.3	57.8 ± 1.7
***10d***	Br	H	Cl	–	H	10.2 ± 2.1	9.8 ± 1.7	16.5 ± 2.6	42.8 ± 2.1
***10e***	Cl	Cl	H	–	H	45.4 ± 6.5	31.6 ± 4.2	103.7 ± 5.9	104.5 ± 7.3
***10f***	H	H	H	–	C_6_H_5_	134.5 ± 4.5	82.7 ± 5.8	114.5 ± 7.6	289.1 ± 10.2
***10 g***	F	H	H	–	C_6_H_5_	93.0 ± 3.1	127.9 ± 6.1	149.8 ± 4.9	257.2 ± 4.3
***10 h***	Cl	H	H	–	C_6_H_5_	89.0 ± 5.2	172.3 ± 7.1	112.5 ± 10.8	249.3 ± 5.8
***10i***	Br	H	H	–	C_6_H_5_	72.2 ± 6.5	59.3 ± 2.7	134.8 ± 5.3	218 ± 3.2
***10 J***	F	F	H	–	C_6_H_5_	45.4 ± 3.2	12.3 ± 1.9	100.7 ± 1.8	101.2 ± 1.3
***10 k***	Cl	Cl	H	–	C_6_H_5_	44.1 ± 7.6	121.2 ± 6.8	102.5 ± 5.2	147.9 ± 6.1
***Cis platin***	–	–	–	–	–	36.5 ± 1.9	12.3 ± 3.3	14.8 ± 0.27	45.2 ± 2.5

### Molecular docking

**Fig. 6:**
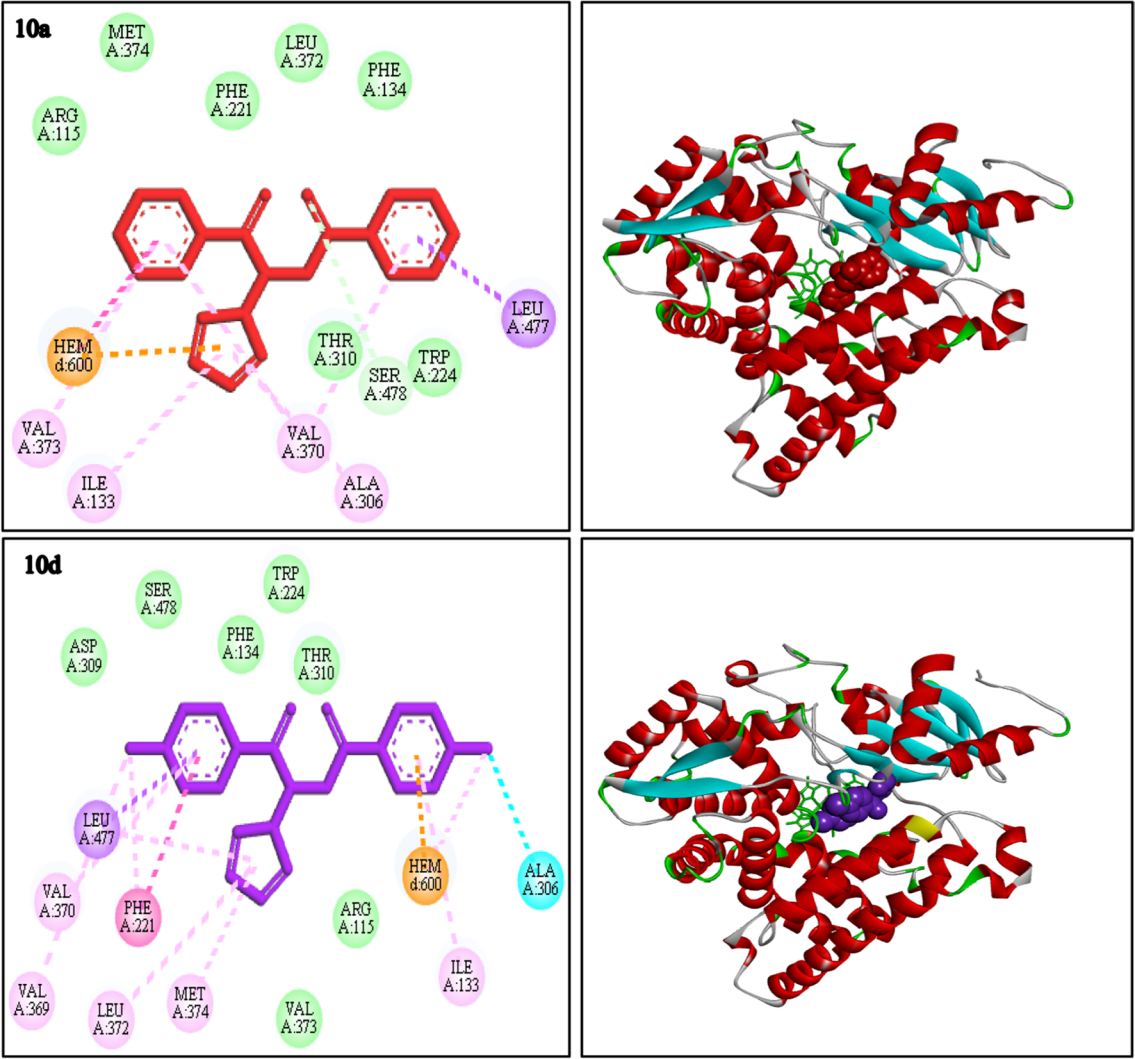
2D interaction diagrams representing the docked conformation of compounds ***10a*** and ***10d*** in the human placental aromatase (3EQM). (Van der waals: green, dark pink: π-π, light pink: π-alkyl, purple: π-sigma, orange: π-cation, blue: halogen bond)

**Fig. 7:**
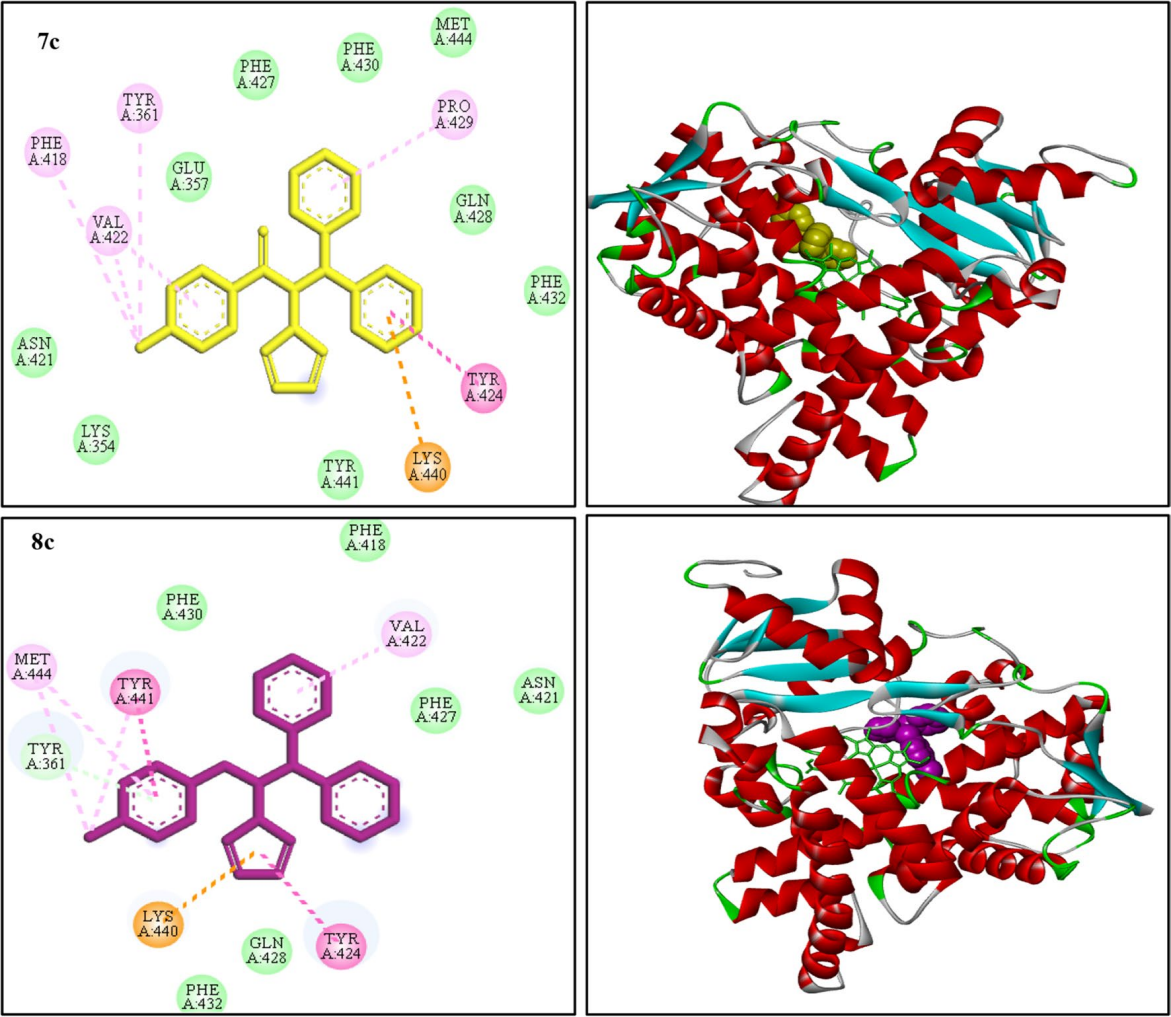
2D interaction diagrams representing the docked conformation of compounds ***7c*** and ***8c*** in the human placental aromatase (3EQM). (Van der waals: green, dark pink: π-π, light pink: π-alkyl, purple: π-sigma, orange: π-cation, blue: halogen bond)

The docking information of four 1,2,4-triazole derivatives possessing the highest (***10a*** and **1*0d*)** and lowest (***7c*** and ***8c*)** cytotoxic activity were shown in Fig. 6 and Fig. 7. Redocking of 4-androstene-3–17-dione as co-crystal ligand, was done to assessment the docking results. The RMSD was obtained 0.40 Å in comparison to its coordination in the crystal structure. The all interaction and score binding of all studied compounds was shown in Table 2.

**Table 2:** The bonding energies (kcal/mol) and the detailed interactions of all synthesized compounds on 3EQM target using AutoDock Vina

Entry	Amino Acid	Ligand involved moiety	Type of interaction	B.E (kcal/mol)	Entry	Amino Acid	Ligand involved moiety	Type of interaction	B.E (kcal/mol)
***7a***	Met 374	benzyl	pi-sulfur	− 9.9	***10c***	HEM 600	benzoyl	pi-cation	− 9.2
	Leu 477, Ile 133, Ala 306, Val 370	1,2,4-triazole & benzoyl	pi-alkyl			Phe 134, Phe 221	1,2,4-triazole & benzoyl	pi-pi	
	HEM 600	benzoyl	pi-cation			Leu 372, Ser 478	1,2,4-triazole & C = O	Carbon hydrogen bond	
	Thr 310, Arg 115, Val 373, Phe 134, Leu 372, Phe 221, Ser 478, Trp 224	–	Vander waals			Ala 306, Ile 133, Val 370, Met 374	benzoyl & 1,2,4-triazole	pi-alkyl & alkyl	
***7b***	Val 370, Leu 477, Val 373, Ala 306, Ile 133, Ile 305, Phe 221, Trp 224	benzyl & benzoyl & 1,2,4-triazole	pi-alkyl & alkyl	− 10.5		Arg 115, Trp 224, Val 373, Thr 310, Leu 477, Asp 309	–	Vander waals	
	HEM 600	benzyl & benzoyl	pi-pi		***10d***	Ala 306	benzoyl	halogen	− 7.9
	Arg 115	benzyl	pi-cation			HEM 600	benzoyl	pi-cation	
	Glu 302, Ser 478, Phe 134, Met 374, Leu 372, Thr 310, Asp 309	–	Vander waals			Leu 477	benzoyl	pi-sigma	
***7c***	Tyr 424	di-phenyl	pi-pi	− 8.2		Phe 221	benzoyl	pi-pi	
	Lys 440	di-phenyl	pi-cation			Val 370, Val 369, Leu 372, Met 374, Ile 133, HEM 600, Phe 221	benzoyl & 1,2,4-triazole	alkyl & pi-alkyl	
	Phe 418, Val 422, Tyr 361, Pro 429	benzoyl & di-phenyl	pi-alkyl &| alkyl			Asp 309, Ser 478, Phe 134, Trp 224, Thr 310, Val 373, Arg 115	–	Vander waals	
	Asn 421, Lys 354, Glu 357, Phe 427, Phe 430, Met 444, Gln 428, Phe 432, Tyr 441	–	Vander waals		***10e***	Arg 115	1,2,4- triazole	Hydrogen bond	− 9.7
***7d***	Lys 440	di-phenyl	pi-alkyl	− 7.9		Ser 478	C = O	Carbon hydrogen bond	
	Tyr 424, Tyr 441	benzoyl & di-phenyl	pi-pi			HEM 600	benzoyl	pi-cation	
	Gln 428	C = O	Hydrogen bond			HEM 600	benzoyl	pi-sigma	
	Phe 432, Phe 430, Met 444, Tyr 361, Pro 429, Phe 427, Val 422, Phe 418	–	Vander waals			Ile 133, Phe 134, Ala 306, Val 370, Met 374, Val 373	benzoyl & 1,2,4-triazole	alkyl & pi-alkyl	
***7e***	Tyr 441, Phe 430, Met 444, Lys 440, Pro 429	1,2,4-triazole & benzoyl	pi-alkyl & alkyl	− 7.3		Leu 372, Leu 477, Phe 221, Asp 309, Trp 224, Thr 310	–	Vander waals	
	Tyr 424	di-phenyl	pi-pi		***10f***	Lys 440	C = O	Carbon hydrogen bond	− 8.9
	Lys 440	1,2,4-triazole	pi-cation			Tyr 361	benzoyl	pi-pi	
	Pro 429	1,2,4-triazole	Carbon hydrogen bond			Tyr 424	phenyl	pi-pi	
	Lys 440	1,2,4-triazole	Hydrogen bond			Pro 429	benzoyl	pi-alkyl	
	Phe 432, Gln 428, Phe 427	–	Vander waals			Val 422, Phe 427, Phe 432, Gln 428, Met 444, Phe 430, Tyr 441	–	Vander waals	
***8a***	Arg 115	benzyl	pi-cation	9.4	***10 g***	Tyr 424	phenyl	pi-pi	− 7.5
	HEM 600	benzyl	pi-pi			Pro 429	benzoyl	pi-alkyl	
	Met 374, Ala 306, Thr 310, Ile 133, Trp 224, Phe 221, Ser 478, Phe 134, Leu 372	–	Vander waals			Phe 418, Tyr 361, Tyr 441, Lys 440, Phe 430, Phe 432, Gln 428, Val 422, Phe 427	–	Vander waals	
	Val 373, Val 370, Leu 477	benzyl	pi-alkyl		***10 h***	Glu 357	benzoyl	pi-anion	-7.2
***8b***	Phe 432, Tyr 424, Pro 429, Met 444	benzyl & di-phenyl	pi-alky	− 7.8		Tyr 424, Tyr 361	benzoyl & phenyl	pi-pi	
	Tyr 424, Tyr 441	benzyl & di-phenyl	pi-pi			Val 422	benzoyl	pi-alkyl	
	Tyr 361	di-phenyl	pi-donor hydrogen bond			Phe 432, Tyr 441, Lys 440, Gln 428, Phe 430, Pro 429, Phe 427, Phe 418, Lys 354, Asn 421	–	Vander waals	
	Phe 427, Gln 428, Gly 433, Lys 440, Phe 430	–	Vander waals		***10i***	Tyr 244	1,2,4-triazole	Hydrogen bond	− 6.7
***8c***	Met 444, Val 422, Tyr 441	benzyl & di-phenyl	pi-alkyl & alkyl	− 7.8		Asp 476	phenyl	pi-anion	
	Tyr 441, Tyr 424	benzyl & 1,2,4-triazole	pi-pi			Ile 474	benzoyl	pi-sigma	
	Tyr 361	benzyl	pi-donor hydrogen bond			Ala 226, Ile 474	1,2,4-triazole & benzoyl	alkyl & pi-alkyl	
	Lys 440	1,2,4-triazole	pi-cation			His 475, Gln 225, Lys 230, Gly 69, Ile 229, Phe 65, Leu 66, Trp 67	–	Vander waals	
	Phe 430, Phe 418, Phe 427, Asn 421, Gln 428, Phe 432	–	Vander waals		***10j***	Lys 440, Gln 428	1,2,4-triazole & C = O	Hydrogen bond	− 7.8
***10a***	Ser 478	C = O	Carbon hydrogen bond	− 9.4		Lys 440	1,2,4-triazole	pi-cation	
	HEM 600	1,2,4-triazole	pi-cation			Tyr 361	phenyl	pi-donor hydrogen bond	
	Leu 477	benzoyl	pi-sigma			Tyr 424, Tyr 441	benzoyl & 1,2,4-triazole & phenyl	pi-pi	
	HEM 600	benzoyl	pi-pi			Pro 429, Met 444	Benzoyl & phenyl	pi-alkyl	
	Val 373, Ile 133, Val 370, Ala 306	1,2,4-triazole & benzoyl	pi-alkyl			Phe 432, Val 422, Phe 418, Phe 427, Phe 430,	––-	Vander waals	
	Arg 115, Met 374, Phe 221, Leu 372, Phe 134, Thr 310, Trp 224	–	Vander waals		***10 k***	Asn 421	1,2,4-triazole	Hydrogen bond	− 7.3
***10b***	Met 374	benzoyl	pi-sulfur	− 9.3		Glu 357	phenyl	pi-anion	
	HEM 600	1,2,4-triazole	pi-cation			Tyr 361	phenyl	pi-pi	
	Phe 221, Val 370, Val 369, Ala 306, Ile 133	1,2,4-triazole & benzoyl	alkyl & pi-alkyl			Tyr 424, Lys 440, Tyr 441, Phe 430, Pro 429	benzoyl	alkyl & pi-alkyl	
	Thr 310, Asp 309, Ser 478, Trp 224, Leu 477, Phe 134, Arg 115, Val 373, Leu 372	–	Vander waals			Met 444, Lys 354, Phe 418, Gln 428, Phe 427, Val 422,	–	Vander waals	

Compound ***10a*** through its carbonyl group interacted with Ser 478 via hydrogen bond and also, formed π-cation and π-π interactions with HEM. The phenyl ring of ***10a*** formed π-sigma bond with Leu 477 and also, some π-alkyl interactions with Val 373, Ile 133, Val 370 and Ala 306 residues were observed. In addition, hydrophobic interactions with Thr 310, Trp 224, Arg 115, Met 374, Phe 221, Leu 372, and Phe 134 residues were existence which led to desire affinity to aromatase enzyme. On the other hand, compound ***10d*** had same similarity interactions with ***10a*** as one of the best compound such as, π-π, π-sigma and π-alkyl with Leu 477, Val 370, Val 369, Phe 221, Leu 372, Met 374 and Ile 133 residues. Compound ***10d*** also, formed halogen bond and π-cation interaction with Ala 306 amino acid residue and HEM group, respectively. These interactions confirmed that compounds ***10a*** and ***10d*** were successfully bound to the aromatase enzyme via most of the previously binding interactions of co-crystal ligand of aromatase enzyme (3EQM) (natural substrate androstenedione (AD)), such as Phe134, Trp224, Val370, Val373, Met374 and HEM [49, 50].

Moreover, compound ***7c*** involved π-π and π-cation interactions with Tyr 424 and Lys 440 residues through its phenyl moiety. In addition, some π-alkyl interactions with Pro 429, Tyr 361, Val 422 and Phe 418 residues were observed. The groups of Glu 357, Phe 427, Phe 430, Met 444, Gln 428, Phe 432, Asn 421, Lys 354 and Tyr 441 residues involved to create a pocket around ***7c*** by van der Waals forces. As it was shown in Fig. 7, the most important residues in binding of ***8c*** was π-π and π-cation interactions between 1,2,4-triazole moiety and Tyr 424 and Lys 440 residues. Also, some π-π and π-alkyl interactions with Tyr 441, Val 422 and Met 444 residues were seen. A pocket of Phe 430, Phe 418, Phe 427, Asn 421, Gln 428 and Phe 432 residue were observed a round of ***8c*** with van der Waals forces. 

### In silico ADME modeling study

ADME properties of the synthesized compounds were determined using SwissADME online software [51]. As depicted in Table 3, all of the compounds represented admissible molecular weight (MW < 500). They had desire lipophilicity (logP) values. In addition, the hydrogen bond properties including hydrogen bond donor (HBD), hydrogen bond acceptor (HBA) and rotatable bond (RB) are reasonable. According to rule of five, total polar surface area of all compounds are in accepted range ≤ 140 Å. Based on our results, all of the compounds indicated desire potential for oral bioavailability [52].

**Table 3:** Physiochemical properties of the synthesized compounds ***7a-e***, ***8a-c*** and ***10a-k***

Entry	MW^a^	LogP^b^	HBD^c^	HBA^d^	TPSA (Å)^e^	RB^f^	Lipinski/Veber violation
*7a*	277.32	2.39	0	3	47.78	5	0
*7b*	346.21	3.40	0	3	47.78	5	0
*7c*	387.86	4.01	0	3	47.78	6	0
*7d*	432.31	4.11	0	3	47.78	6	0
*7e*	422.32	4.49	0	3	47.78	6	1
*8a*	263.34	3.32	0	2	30.71	5	0
*8b*	373.88	4.93	0	2	30.71	6	1
*8c*	418.33	5.04	0	2	30.71	6	1
*10a*	305.53	1.73	0	4	64.85	6	0
*10b*	339.78	2.23	0	4	64.85	6	0
*10c*	384.23	2.34	0	4	64.85	6	0
*10d*	418.67	2.84	0	4	64.85	6	0
*10e*	374.22	2.72	0	4	64.85	6	0
*10f*	381.43	2.84	0	4	64.85	7	0
*10 g*	399.42	3.22	0	5	64.85	7	0
*10 h*	415.87	3.32	0	4	64.85	7	0
*10i*	460.32	3.42	0	4	64.85	7	0
*10j*	417.41	3.59	0	6	64.85	7	0
*10 k*	450.32	3.79	0	4	64.85	7	0
Lipinski/Veber’s Rules	≤ 500	≤ 5	≤ 5	≤ 10	≤ 140	≤ 10	≤ 1

^a^Molecular weight (MW). ^b^ Logarithm of partition coefficient between n-octanol and water (LogP). ^c^ Number of hydrogen bond donors (HBD). ^d^ Number of hydrogen bond acceptors (HBA). ^e^ Topological polar surface area (TPSA). ^f^ Number of rotatable bonds (RB)

## Experimental section

### Chemistry

All reagents and solvents with analytical grade were purchased from commercial sources (Merck & Sigma Aldrich) and used without further purification. IR spectra (KBr, cm^−1^) were recorded using a Perkin Elmer IR instrument. ^1^H-NMR spectra were recorded in CDCl_3_ on Bruker 500 MHz spectrophotometer using tetramethylsilane (TMS) as an internal standard. Chemical shift values were expressed in ppm scale (δ) and coupling constants were reported in hertz (Hz). An Agilent spectrometer was used for Mass spectra recordation. All melting points were measured with an Electro-thermal IA 9100 apparatus and were uncorrected. The progress of reactions was monitored using thin layer chromatography (TLC) sheets pre-coated with UV fluorescent silica gel Merck 60F_254_ and the spots were visualized using UV lamp. The purification of the synthesized compounds was performed by column chromatography.

#### General procedure for the synthesis of intermediates *3a-f*

The appropriate AlCl_3_ (60 mmol) was added to a stirred solution of phenyl halides (50 mmol) in dichloromethane (30 mL). After stirring for 30 min at room temperature, the mixture was cooled to 0 ^ο^C and a solution of chloroacetyl chloride (54 mmol) in dichloromethane (20 mL) was added dropwise to it. The resulting mixture was stirred at room temperature for 24 h. After this time, 50 mL of HCl solution (5%) was slowly added and the reaction mixture was extracted with dichloromethane (3 × 30 mL) and then washed with NaHCO_3_ (20 mL), water (2 × 20 mL) and brine (20 mL), respectively. The organic layer was dried over anhydrous Na_2_SO_4_ and concentrated under vacuum. Finally, the obtained precipitate was recrystallized from *n*-hexane to give intermediates **3a-f**.

#### General procedure for the synthesis of intermediates *5a-f*

1,2,4-triazole (48 mmol) and NaHCO_3_ (48 mmol) were added to a stirred solution of intermediates ***3a-f*** (40 mmol) in toluene. The reaction mixture was refluxed for 20 h. After this time, the reaction mixture was quenched with an ice bath and extracted with ethyl acetate (3 × 30 mL). Then, the organic phase was washed with water (2 × 20 mL) and brine (10 mL), dried over anhydrous Na_2_SO_4_ and concentrated in vacuum. Finally, the residue was recrystallized from diethyl ether to afford the desired compounds ***5a*-*f***.

#### General procedure for the synthesis of compounds *7a-e*

A solution of intermediates ***5a-f*** (6 mmol) in acetonitrile (10 mL) was added to a suspension of NaH (8 mmol) in acetonitrile (30 mL) and the resulting mixture was stirred at room temperature for 1 h. Then, a solution of benzhydryl bromide or benzyle bromide (6 mmol) in 10 mL acetonitrile was added dropwise and the mixture was heated under reflux for 24 h. After cooling to room temperature, the solvent was evaporated in vacuum, 50 mL water was added and the mixture was extracted with dichloromethane (3 × 30 mL). In the following, the organic layers were dried over Na_2_SO_4_ and purified by column chromatography on silica gel eluting with ethyl acetate and petroleum ether (3:1) to afford desired products ***7a-e*.**

##### 1,3-diphenyl-2-(1H-1,2,4-triazol-1-yl) propan-1-one (***7a***)

Yield: 46%. M. P.: 145–148 °C; IR (KBr, cm^−1^): 3101 (C-H, aromatic), 2928.8 (C-H, aliphatic), 1690.0 (C = O, ketones), 1594.2 (C = N), 1283.7 (C–N stretch, aromatic).^1^H-NMR (500 MHz, CDCl_3_) δ (ppm): 8.28 (s, 1H, triazole), 7.92 (d, *J* = 7.5 Hz, 2H, Ar–H-CO), 7.91 (s, 1H, triazole), 7.59 (t, *J* = 7.5 Hz, 1H, Ar–H-CO), 7.46 (t, *J* = 7.5 Hz, 2H, Ar–H-CO), 7.18–7.24 (m, 3H, Ar–H), 7.01 (d, *J* = 6.8 Hz, 2H, Ar–H), 6.24 (dd, *J* = 8.8, 5.8 Hz, 1H, CH), 3.55 (dd, *J* = 14.3, 5.8 Hz, 1H, CH_2_), 3.41 (dd, *J* = 14.3, 9.0 Hz, 1H, CH_2_). ^13^C-NMR (75 MHz, CDCl_3_) δ: 193.8, 151.4, 143.6, 135.6, 134.8, 134.7, 129.5, 129.4, 129.3, 129.1, 127.9, 65.5, 39.0. MS m/z (%): 277.2 (5) [M^+^], 208.1 (45), 105.2 (100), 91.0 (60), 77.1 (100), 51.0 (15). Elem. anal. calcd. For C_17_H_15_N_3_O (277.2); C, 73.63; H, 5.45; N, 15.15. Found: C, 73.60; H, 5.42; N, 15.12.

##### 1-(2,4-dichlorophenyl)-3-phenyl-2-(1H-1,2,4-triazol-1-yl) propan-1-one (*7b*)

Yield: 52%. M.P.: 121–123 °C; IR (KBr, cm^−1^): 3133.7 (C–H stretch, aromatic), 2926.1 (C-H, aliphatic), 1723.8 (C = O, ketone), 1587.1 (C = N), 1294.1, 1248.1 (C–N stretch, aromatic), 1149.0 (Ar-Cl). ^1^H-NMR (500 MHz, CDCl_3_) δ (ppm): 8.18 (s, 1H, triazole), 7.79 (s, 1H, triazole), 7.73 (d, *J* = 8.6 Hz, 1H, 2,4-diCl-Ar–H, H-6), 7.58 (s, 1H, 2,4-diCl-Ar–H, H-3), 7.38 (d, *J* = 8.6 Hz, 1H, 2,4-diCl-Ar–H, H-5), 7.18–7.23 (m, 3H, Ar–H), 6.98 (d, *J* = 6.7 Hz, 2H, Ar–H), 6.18 (dd, *J* = 8.5, 6.0 Hz, 1H, CH), 3.73 (dd, *J* = 14.2, 6.0 Hz, 1H, CH_2_), 3.60 (dd, *J* = 14.2, 8.6 Hz, 1H, CH_2_). MS m/z (%): 345.3 (4) [M^+^], 279.9 (8), 189.9 (30), 144.9 (23), 173.0 (70), 109.0 (22), 91.1 (100), 74.0 (27), 63.1 (18), 50.1 (8). Elem. anal. calcd. For C_17_H_13_Cl_2_N_3_O (345.3); C, 58.98; H, 3.78; N, 12.14. Found: C, 58.89; H, 3.72; N, 12.10.

##### 1-(4-chlorophenyl)-3,3-diphenyl-2-(1H-1,2,4-triazol-1-yl) propan-1-one (*7c*)

Yield: 63%. M.P.: 140–142 °C; IR (KBr, cm^−1^): 3101.6 (C-H, aromatic), 2926.1 (C-H, aliphatic), 1691.4 (C = O, ketones), 1588.7 (C = N stretch, aromatic), 1275.4 (C–N stretch, aromatic), 1093.1 (Ar-Cl). ^1^H-NMR (500 MHz, CDCl_3_) δ (ppm): 8.33 (s, 1H, triazole), 7.81 (d, *J* = 8.2 Hz, 2H, Ar–H-CO, H-2 and H-6), 7.81 (s, 1H, triazole), 7.34 (d, *J* = 8.3 Hz, 2H, Ar–H-CO, H-3 and H-5), 7.27 (d, *J* = 7.5 Hz, 2H, Ar–H), 7.23–7.13 (m, 7H, Ar–H), 7.10 (t, *J* = 7.3 Hz, 1H, Ar–H), 6.79 (d, *J* = 11.6 Hz, 1H, CH–N), 5.09 (d, *J* = 11.6 Hz, 1H, CH-(Ph)_2_). MS m/z (%): 387.1 (12) [M^+^], 317.1 (45), 242.1 (100), 178.1 (70), 167.3 (100), 152.0 (70), 141.0 (55), 111.0 (100), 91.0 (38), 75.1 (35), 51.1 (7). Elem. anal. calcd. For C_23_H_18_ClN_3_O (387.87); C, 71.22; H, 4.68; N, 10.83. Found: C, 71.20; H, 4.58; N, 10.80.

##### 1-(4-bromophenyl)-3,3-diphenyl-2-(1H-1,2,4-triazol-1-yl) propan-1-one (*7d*)

Yield: 58%. M.P.: 110–113 °C; IR (KBr, cm^−1^): 3103.3 (C-H, aromatic), 2973.0 (C-H, aliphatic), 1692.8 (C = O, ketones), 1583.1 (C = N stretch, aromatic), 1287.8 (C–N stretch, aromatic), 1012.4 (Ar-Br). ^1^H-NMR (500 MHz, CDCl_3_) δ (ppm): 8.13 (s, 1H, triazole), 7.77 (s, 1H, triazole), 7.71 (d, *J* = 8.4 Hz, 2H, Ar–H-CO, H-2 and H-6), 7.51 (d, *J* = 8.4 Hz, 2H, Ar–H-CO, H-3 and H-5), 7.26–7.11 (m, 10 H, Ar–H), 6.73 (d, *J* = 11.6 Hz, 1H, CH–N), 5.07 (d, *J* = 11.6 Hz, 1H, CH-(Ph)_2_). MS m/z (%): 431.1 (5) [M^+^], 363.0 (25), 248.1 (25), 207.0 (22), 184.9 (75), 167.3 (100), 152.0 (100), 128.0 (8), 91.0 (28), 76.1 (33), 51.0 (7). Elem. anal. calcd. For C_23_H_18_BrN_3_O (433.1); C, 63.90; H, 4.20; N, 9.72. Found: C, 63.85; H, 4.16; N, 9.68.

##### 1-(2,4-dichlorophenyl)-3,3-diphenyl-2-(1H-1,2,4-triazol-1-yl) propan-1-one (*7e*)

Yield: 48%. M.P.: 118–121˚C. IR (KBr, cm^−1^): 3130.3 (C–H stretch, aromatic), 2923.9 (C-H, aliphatic), 1700.1 (C = O, ketone), 1578.6 (C = N), 1276.3 (C–N stretch, aromatic), 1137.6 (Ar-Cl). ^1^H-NMR (500 MHz, CDCl_3_) δ (ppm): 8.12 (s, 1H, triazole), 7.79 (s, 1H, triazole), 7.31 (d, *J* = 1.8 Hz, 1H, 2,4-diCl-Ar–H, H-6), 7.30 (s, 1H, 2,4-diCl-Ar–H, H-3), 7.23–7.15 (m, 8H, Ar–H), 7.14 (d, *J* = 1.8 Hz, 1H, 2,4-diCl-Ar–H, H-5), 7.13–7.10 (m, 2H, Ar–H), 6.74 (d, *J* = 11.6 Hz, 1H, CH–N), 4.94 (d, *J* = 11.6 Hz, 1H, CH-(Ph)_2_). MS m/z (%): 421.2 (3) [M^+^], 351.0 (15), 276.0 (68), 248.1 (45), 165.1 (100), 152.0 (52), 109.0 (20), 91.0 (33), 77.1 (12), 51.1 (5). Elem. anal. calcd. For C_23_H_17_Cl_2_N_3_O (421.2); C, 65.41; H, 4.06; N, 9.95. Found: C, 65.38; H, 4.02; N, 9.92.

#### General procedure for the synthesis of compounds *8a-c*

A mixture of compound ***7a*** or ***7c-d*** (1.6 mmol), hydrazine monohydrate (8 mmol) and potassium hydroxide (8 mmol) in ethylene glycol (50 mL) was heated at 170 ˚C for 4 h. Then, the reaction mixture was cooled to room temperature, quenched with water (500 mL) and acidified to pH = 1 with concentrated hydrochloric acid and extracted with chloroform (3 × 30 mL). Afterwards, the organic layer was washed with brine and dried over Na_2_SO_4_. The crude product was purified by column chromatography on silica gel eluting with ethyl acetate and petroleum ether (1:1) to give pure compounds ***8a-c***.

##### 1-(1,3-diphenylpropan-2-yl)-1*H*-1,2,4-triazole (*8a*)

Yield: 39%. M.P.: 81–83 °C, IR (KBr, cm^−1^): 3103.7 (C-H, aromatic), 2931.8 (C-H, aliphatic), 1598.8 (C = N), 1285.8 (C–N stretch, aromatic). ^1^H-NMR (500 MHz, CDCl_3_) δ (ppm): 8.17 (s, 1H, triazole), 7.85 (s, 1H, triazole), 7.29–7.27 (m, 6H, Ar–H, H-2, H-4 and H-6), 7.13–7.11 (m, 4H, Ar–H, H-3 and H-5), 4.64 (m, 1H, CH), 3.70 (d, *J* = 8.8 Hz, 4H, CH_2_). MS m/z (%): 263.1 (10) [M^+^], 201.1 (17), 172.0 (9) 105.2 (100), 91.0 (73), 77.1 (100), 63.1 (12), 51.1 (15). Elem. anal. calcd. For C_17_H_17_N_3_ (263.1); C, 77.54; H, 6.51; N, 15.96. Found: C, 77.35; H, 6.49; N, 15.85.

##### 1-(3-(4-chlorophenyl)-1,1-diphenylpropan-2-yl)-1*H*-1,2,4-triazole (*8b*)

Yield: 41%. M.P.: 93–96 °C; IR (KBr, cm^−1^): 3103.4 (C-H, aromatic), 2959.5 (C-H, aliphatic), 1591.1 (C = N), 1278.6 (C–N stretch, aromatic). ^1^H-NMR (500 MHz, CDCl_3_) δ (ppm): 8.29 (s, 1H, triazole), 7.78 (s, 1H, triazole), 7.46 (d, *c* = 8.4 Hz, 2H, 4-Cl-Ar–H, H-2 and H-6), 7.28–7.26 (d, *J* = 7.1 Hz, 2H, Ar–H), 7.24–7.08 (m, 8H, Ar–H), 7.05 (d, *J* = 8.4 Hz, 2H, 4-Cl-Ar–H, H-3 and H-5), 4.67 (m, 1H, 4-Cl-Ar-CH_2_-CH), 3.89 (d, *J* = 11.2 Hz, 1H, Ar–CH), 3.42 (dd, *J* = 14.2, 6.1 Hz, 1H, CH_2_), 3.27 (dd, *J* = 14.3, 8.5 Hz, 1H, CH_2_). MS m/z (%): 373.1 (5) [M^+^], 306.1 (8), 245.1 (15) 173.1 (35), 155.1 (30), 126.0 (100), 111.0 (45), 103.1 (33), 77.0 (25), 63.0 (4), 51.1 (7). Elem. anal. calcd. For C_23_H_20_ClN_3_ (373.13); C, 73.89; H, 5.39; N, 11.24. Found: C, 73.80; H, 5.28; N, 11.19.

##### 1-(3-(4-bromophenyl)-1,1-diphenylpropan-2-yl)-1*H*-1,2,4-triazole (*8c*)

Yield: 45%. M.P.: 88–91 °C; IR (KBr, cm^−1^): 3105.1 (C-H, aromatic), 2970.5 (C-H, aliphatic), 1585.2 (C = N), 1288.1 (C–N stretch, aromatic). ^1^H-NMR (500 MHz, CDCl_3_) δ (ppm): 8.18 (s, 1H, triazole), 7.81 (s, 1H, triazole), 7.57 (d, *J* = 8.4 Hz, 2H, 4-Br-Ar–H, H-2 and H-6), 7.28–7.26 (d, *J* = 7.0 Hz, 2H, Ar–H), 7.27–7.10 (m, 8H, Ar–H), 7.08 (d, *J* = 8.5 Hz, 2H, 4-Br-Ar–H, H-3 and H-5), 4.59 (m, 1H, 4-Cl-Ar-CH_2_-CH), 3.75 (d, *J* = 11.5 Hz, 1H, Ar–CH), 3.50 (dd, *J* = 14.2, 6.1 Hz, 1H, CH_2_), 3.37 (dd, *J* = 14.2, 8.5 Hz, 1H, CH_2_). MS m/z (%): 417.0 (8) [M^+^], 363.0 (10), 286.0 (15) 248.1 (25), 184.9 (55), 169.3 (100), 155.0 (48), 128.1 (15), 104.0 (15), 91.0 (23), 76.0 (43), 51.0 (7). Elem. anal. calcd. For C_23_H_20_BrN_3_ (417.0); C, 66.04; H, 4.82; N, 10.04. Found: C, 66.01; H, 4.79; N, 10.02.

#### General procedure for the synthesis compounds *10a-k*

A solution of intermediates ***5a-f*** (6 mmol) in acetonitrile (10 mL) was added to a suspension of NaH (8 mmol) in acetonitrile (30 mL) and the resulting mixture was stirred at room temperature for 1 h. Then, a solution of 2-chloro-2-phenyl acetophenone or halogenated phenacyl chloride (6 mmol) in 10 mL acetonitrile was added dropwise and the mixture was heated under reflux for 24 h. After cooling to room temperature, the solvent was evaporated in vacuum, 50 mL water was added and the mixture was extracted with dichloromethane (3 × 30 mL). In the following, the organic layers were dried over Na_2_SO_4_ and purified by column chromatography on silica gel eluting with ethyl acetate and petroleum ether (3:1) to afford desired products ***10a-k***.

##### 4-diphenyl-2-(1*H*-1,2,4-triazol-1-yl) butane-1,4-dione (*10a*)

Yield: 55%. M.P.: 115–118 °C, IR (KBr, cm^−1^): 3093.5 (C–H stretch, aromatic), 2911.0 (C-H, aliphatic), 1691.0, 1668.8 (C = O, ketone), 1595.7 (C = N), 1271.3 (C–N stretch, aromatic). ^1^H-NMR (500 MHz, CDCl_3_) δ (ppm): 8.29 (s, 1H, triazole), 7.96 (d, *J* = 7.2 Hz, 4H, Ar–H-CO and Ar′-H–CO, H-2 and H-6), 7.90 (s, 1H, triazole), 7.58 (t, *J* = 7.1 Hz, 2H, Ar–H-CO and Ar′-H–CO, H-4), 7.48 (d, 4H, Ar–H-CO and Ar′-H–CO, H-3 and H-5), 6.69 (t, *J* = 6.0 Hz, 1H, CH), 4.15 (dd, *J* = 18.0, 6.6 Hz, 1H, CH_2_), 3.83 (dd, *J* = 17.8, 5.9 Hz, 1H, CH_2_). MS m/z (%): 305.3 (3) [M^+^], 236.1 (10), 276.1 (100), 223.1 (80), 200.1 (35), 178.1 (10), 131.0 (8), 105.2 (98), 77.0 (100), 63.0 (5), 51.1 (20). Elem. anal. calcd. For C_18_H_15_N_3_O_2_ (305.12); C, 70.81; H, 4.95; N, 13.76. Found: C, 70.75; H, 4.92; N, 13.72.

##### 1-(4-chlorophenyl)-4-phenyl-2-(1H-1,2,4-triazol-1-yl) butane-1,4-dione (*10b*)

Yield: 67%. M.P.: 131–133 °C; IR (KBr, cm^−1^): 3092.8 (C–H stretch, aromatic), 2920.3 (C-H, aliphatic), 1692.6, 1655.5 (C = O, ketone), 1586.3 (C = N), 1272.1 (C–N stretch, aromatic), 1090.4 (Ar-Cl). ^1^H-NMR (500 MHz, CDCl_3_) δ (ppm): 8.25 (s, 1H, triazole), 7.96 (d, *J* = 7.2 Hz, 2H, Ar–H-CO, H-2 and H-6), 7.90 (s, 1H, triazole), 7.83 (d, *J* = 8.5 Hz, 2H, 4-Cl-Ar–H-CO, H-2 and H-6), 7.44 (t, *J* = 7.1 Hz, 1H, Ar–H-CO, H-4), 7.39 (d, *J* = 8.5 Hz, 2H, 4-Cl-Ar–H-CO, H-3 and H-5), 7.30 (d, *J* = 7.1 Hz, 2H, Ar–H-CO, H-3 and H-5), 6.65 (t, *J* = 6.2 Hz, 1H, CH), 4.13 (dd, *J* = 18.0, 6.6 Hz, 1H, CH_2_), 3.80 (dd, *J* = 17.9, 5.9 Hz, 1H, CH_2_). MS m/z (%): 339.1 (8) [M^+^], 287.1 (13), 263.0 (25), 178.1 (10), 170.0 (18), 139.1 (100), 105.1 (100), 77.0 (83), 51.1 (13). Elem. anal. calcd. For C_10_H_14_ClN_3_O_2_ (339.1); C, 63.63; H, 4.15; N, 12.37. Found: C, 63.59; H, 4.10; N, 12.35.

##### 1-(4-bromophenyl)-4-phenyl-2-(1H-1,2,4-triazol-1-yl) butane-1,4-dione (*10c*)

Yield: 44%. M.P.: 149–151 °C. IR (KBr, cm^−1^): 3073.7 (C–H stretch, aromatic), 2925.4 (C-H, aliphatic), 1697.1, 1659.5 (C = O, ketone), 1589.5 (C = N), 1279.1 (C–N stretch, aromatic), 1009.6 (Ar-Br).^1^H-NMR (500 MHz, CDCl_3_) δ (ppm): 8.34 (s, 1H, triazole), 7.96 (d, *J* = 7.1 Hz, 2H, Ar–H-CO, H-2 and H-6), 7.92 (s, 1H, triazole), 7.74 (d, *J* = 8.3 Hz, 2H, 4-Br-Ar–H-CO, H-2 and H-6), 7.56 (d, *J* = 8.3 Hz, 2H, 4-Br-Ar–H-CO, H-3 and H-5), 7.43 (t, *J* = 7.1 Hz, 1H, Ar–H-CO, H-4), 7.30 (d, *J* = 7.1 Hz, 2H, Ar–H-CO, H-3 and H-5), 6.69 (t, *J* = 6.4 Hz, 1H, CH), 4.15 (dd, *J* = 18.0, 6.7 Hz, 1H, CH_2_), 3.82 (dd, *J* = 18.0, 6.1 Hz, 1H, CH_2_). MS m/z (%): 383.0 (3) [M^+^], 303.0 (12), 236.1 (15), 200.1 (17), 183.0 (17), 154.9 (10), 105.1 (98), 77.1 (100), 51.1 (18). Elem. anal. calcd. For C_18_H_14_BrN_3_O_2_ (383.0); C, 56.27; H, 3.67; N, 10.94. Found: C, 56.20; H, 6.60; N, 10.92.

##### 1-(4-bromophenyl)-4-(4-chlorophenyl)-2-(1H-1,2,4-triazol-1-yl) butane-1,4-dione (*10d*)

Yield: 46%. M.P.: 162–165 °C, IR (KBr, cm^−1^): 3069.2 (C–H stretch, aromatic), 2922.3 (C-H, aliphatic), 1689.7 (C = O, ketone), 1590.7 (C = N), 1279.3 (C–N stretch, aromatic), 1093.4 (Ar-Cl), 1012.4 (Ar-Br). ^1^H-NMR (500 MHz, CDCl_3_) δ (ppm): 8.35 (s, 1H, triazole), 8.04 (d, *J* = 8.4 Hz, 2H, 4-Cl-Ar–H-CO, H-2 and H-6), 7.94 (s, 1H, triazole), 7.88 (d, *J* = 8.4 Hz, 2H, 4-Br-Ar–H-CO, H-2 and H-6), 7.40 (d, *J* = 8.4 Hz, 2H, 4-Br-Ar–H-CO, H-3 and H-5), 7.30 (d, *J* = 8.4 Hz, 2H, 4-Cl-Ar–H-CO, H-3 and H-5), 6.68 (t, *J* = 6.0 Hz, 1H, CH), 4.15 (dd, *J* = 17.9, 6.8 Hz, 1H, CH_2_), 3.82 (dd, *J* = 18.0, 6.2 Hz, 1H, CH_2_). MS m/z (%): 416.9 (3) [M^+^], 335.0 (100), 291.0 (23), 212.0 (30), 182.9 (50), 156.9 (18), 139.10 (100), 111.0 (100), 75.1 (47), 63.0 (10). Elem. anal. calcd. For C_18_H_13_BrClN_3_O_2_ (416.9); C, 51.64; H, 3.13; N, 10.04. Found: C, 51.60; H, 3.09; N, 10.01.

##### 1-(2,4-dichlorophenyl)-4-phenyl-2-(1H-1,2,4-triazol-1-yl) butane-1,4-dione (*10e*)

Yield: 52%. M.P.: 153–156 °C, IR (KBr, cm^−1^): 3098.2 (C–H stretch, aromatic), 2929.0 (C-H, aliphatic), 1710.5, 1673.5 (C = O, ketone), 1581.3 (C = N), 1272.1 (C–N stretch, aromatic), 1137.6 (Ar-Cl). ^1^H-NMR (500 MHz, CDCl_3_) δ (ppm): 8.31 (s, 1H, triazole), 7.94 (s, 1H, triazole), 7.92 (d, *J* = 7.2 Hz, 2H, Ar–H-CO, H-2 and H-6), 7.65 (d, *J* = 8.3 Hz, 1H, 2,4-diCl-Ar–H-CO, H-2), 7.46 (t, *J* = 7.2 Hz, 1H, Ar–H-CO, H-4), 7.22 (s, 1H, 2,4-diCl-Ar–H, H-5), 7.14 (t, *J* = 7.1 Hz, 2H, Ar–H-CO, H-3 and H-5), 7.06 (d, *J* = 8.4 Hz, 1H, 2,4-diCl-Ar–H-CO, H-3), 6.52 (t, *J* = 6.0 Hz, 1H, CH), 4.12 (dd, *J* = 18.2, 5.1 Hz, 1H, CH_2_), 3.79 (dd, *J* = 18.2, 7.0 Hz, 1H, CH_2_. MS m/z (%): 373.2 (4) [M^+^], 338.1 (25), 268.0 (18), 240.0 (5), 172.9 (75), 145.0 (28), 105.2 (100), 91.0 (20), 77.0 (73), 51.0 (13). Elem. anal. calcd. For C_18_H_13_Cl_2_N_3_O_2_ (373.2); C, 57.77; H, 3.50; N, 18.95. Found: C, 57.75; H, 3.48; N, 18.91.

##### 4-triphenyl-3-(1H-1,2,4-triazol-1-yl) butane-1,4-dione (*10f*)

Yield: 64%. M.P.: 185–188 °C; IR (KBr, cm^−1^): 3068.6 (C–H stretch, aromatic), 2924.0 (C-H, aliphatic), 1698.1, 1663.7 (C = O, ketone), 1596.3 (C = N), 1276.8 (C–N stretch, aromatic). ^1^H-NMR (500 MHz, CDCl_3_) δ (ppm): 8.15 (s, 1H, triazole), 7.99 (d, *J* = 7.7 Hz, 2H, Ar–H-CO-CHN, H-2 and H-6), 7.90 (d, *J* = 7.7 Hz, 2H, Ar–H-CO–CH-Ar, H-2 and H-6), 7.87 (s, 1H, triazole), 7.55–7.46 (m, 2H, 2Ar-H–CO, H-4), 7.40 (t, *J* = 7.4 Hz, 4H, 2Ar-H–CO, H-3 and H-5), 7.23–7.18 (m, 3H, Ar–H, H-3, H-4 and H-5), 7.13 (d, *J* = 6.5 Hz, 2H, Ar–H, H-2 and H-6), 6.66 (d, *J* = 10.5 Hz, 1H, CH–N), 5.69 (d, *J* = 10.5 Hz, 1H, CH-Ph). MS m/z (%): 381.2 (8) [M^+^], 312.1 (6), 276.1 (100), 209.1 (35), 170.1 (45), 131.0 (18), 104.8 (100), 90.0 (40), 76.9 (100), 63.1 (7), 51.1 (40). Elem. anal. calcd. For C_24_H_19_N_3_O_2_ (381.4); C, 75.57; H, 5.02; N, 11.02. Found: C, 75.52; H, 5.01; N, 11.02.

##### 1-(4-fluorophenyl)-3,4-diphenyl-2-(1H-1,2,4-triazol-1-yl) butane-1,4-dione (*10 g*)

Yield: 34%. M.P.: 154–157 °C; IR (KBr, cm^−1^): 3094.9 (C–H stretch, aromatic), 2918.0 (C-H, aliphatic), 1695.9, 1658.5 (C = O, ketone), 1598.2 (C = N), 1240.9 (C–N stretch, aromatic), 1275.5 (Ar-F). ^1^H-NMR (500 MHz, CDCl_3_) δ (ppm): 8.28 (s, 1H, triazole), 7.92 (dd, *J* = 8.7, 5.3 Hz, 2H, 4-F-Ar–H-CO, H-3 and H-5), 7.79 (s, 1H, triazole), 7.55 (d, *J* = 7.5 Hz, 2H, Ar–H-CO, H-2 and H-6), 7.23–7.13 (m, 7H, Ar–H and Ar–H-CO, H-3 and H-5), 7.09 (t, *J* = 7.5 Hz, 1H, Ar–H-CO, H-4), 7.04 (t, *J* = 8.6 Hz, 2H, 4-F-Ar–H-CO, H-2 and H-6), 6.81 (d, *J* = 11.6 Hz, 1H, CH–N), 5.09 (d, *J* = 11.6 Hz, 1H, CH-Ph). MS m/z (%): 399.2 (5) [M^+^], 365.4 (4), 332.4 (10), 305.3 (22), 295.1 (10), 226.1 (75), 197.2 (10), 172.0 (18), 149.0 (25), 123.2 (100), 95.1 (100), 75.0 (38), 63.1 (22), 51 (9). Elem. anal. calcd. For C_24_H_18_FN_3_O_2_ (399.2); C, 72.17; H, 4.54; N, 4.76. Found: C, 72.15; H, 4.51; N, 4.75.

##### 1-(4-chlorophenyl)-3,4-diphenyl-2-(1H-1,2,4-triazol-1-yl) butane-1,4-dione (*10 h*)

Yield: 40%. M.P.: 174–176 °C; IR (KBr, cm^−1^): 3100.4 (C–H stretch, aromatic), 2979.5 (C-H, aliphatic), 1696.4, 1659.6 (C = O, ketone), 1588.8 (C = N), 1275.8 (C–N stretch, aromatic), 1092.2 (Ar-Cl). ^1^H-NMR (500 MHz, CDCl_3_) δ (ppm): 8.11 (s, 1H, triazole), 7.94 (d, *J* = 7.4 Hz, 2H, Ar–H-CO, H-2 and H-6), 7.89 (d, *J* = 8.4 Hz, 2H 4-Cl-Ar–H-CO, H-2 and H-6), 7.84 (s, 1H, triazole), 7.57 (d, *J* = 8.5 Hz, 2H 4-Cl-Ar–H-CO, H-3 and H-5), 7.53 (t, *J* = 7.4 Hz, 1H, Ar–H-CO, H-4), 7.541 (t, *J* = 7.4 Hz, 2H, Ar–H-CO, H-3 and H-5), 7.22–21 (m, 3H, Ar–H), 7.12–10 (m, 2H, Ar–H), 6.60 (d, *J* = 10.1 Hz, 1H, CH–N), 5.66 (d, *J* = 10.0 Hz, 1H, CH-Ph). MS m/z (%): 415.1 (4) [M^+^], 397.1 (5), 346.1 (5), 310.1 (15), 276.1 (30), 242.0 (60), 207.0 (25), 197.2 (10), 170.0 (20), 139.1 (100), 104.9 (100), 90.0 (18), 77.1 (100), 63.0 (3), 51.1 (12). Elem. anal. calcd. For C_24_H_18_ClN_3_O_2_ (415.1); C, 69.31; H, 4.36; N, 10.10. Found: C, 69.29; H, 4.32; N, 10.09.

##### 1-(4-bromophenyl)-3,4-diphenyl-2-(1H-1,2,4-triazol-1-yl) butane-1,4-dione (*10i*)

Yield: 48%. M.P.: 168–171 °C; IR (KBr, cm^−1^): 3069.2 (C–H stretch, aromatic), 2922.4 (C-H, aliphatic), 1696.7, 1657.8 (C = O, ketone), 1584.4 (C = N), 1277.1 (C–N stretch, aromatic), 1007.3 (Ar-Br). ^1^H-NMR (500 MHz, CDCl_3_) δ (ppm): 8.16 (s, 1H, triazole), 7.98 (d, *J* = 7.4 Hz, 2H, Ar–H-CO, H-2 and H-6), 7.79 (s, 1H, triazole), 7.76 (d, *J* = 8.5 Hz, 2H, 4-Br-Ar–H-CO, H-2 and H-6), 7.55 (d, *J* = 8.5 Hz, 2H, 4-Br-Ar–H-CO, H-3 and H-5), 7.52 (t, *J* = 7.4 Hz, 1H, Ar–H-CO, H-4), 7.40 (t, *J* = 7.4 Hz, 2H, Ar–H-CO, H-3 and H-5), 7.22–21 (m, 3H, Ar–H), 7.11–10 (m, 2H, Ar–H), 6.57 (d, *J* = 10.4 Hz, 1H, CH–N), 5.64 (d, *J* = 10.4 Hz, 1H, CH-Ph). MS m/z (%): 459.1 (2) [M^+^], 390.0 (3), 354.1 (7), 286.0 (35), 276.1 (25), 207.1 (18), 182.9 (60), 154.9 (30), 105.1 (100), 91.1 (18), 77.1 (85), 51.1 (10). Elem. anal. calcd. For C_24_H_18_BrN_3_O_2_ (459.1); C, 62.62; H, 3.94; N, 9.13. Found: C, 62.60; H, 3.90; N, 9.09.

###### 1-(2,4-difluorophenyl)-3,4-diphenyl-2-(1H-1,2,4-triazol-1-yl) butane-1,4-dione (*10j*)

Yield: 45%. M.P.: 114–117 °C; IR (KBr, cm^−1^): 3062.4 (C–H stretch, aromatic), 2922.7 (C-H, aliphatic), 1676.7 (C = O, ketone), 1611.8 (C = N), 1271.9 (Ar-F). ^1^H-NMR (500 MHz, CDCl_3_) δ (ppm): 8.21 (s, 1H, triazole), 8.03 (s, 1H, triazole), 7.98 (d, *J* = 7.3 Hz, 2H, Ar–H-CO, H-2 and H-6), 7.90 (dt, *J* = 17.1, 9.5 Hz, 1H, 4-F-Ar–H-CO, H-6), 7.71 (t, *J* = 7.3 Hz, 1H, Ar–H-CO, H-4), 7.46 (t, *J* = 7.4 Hz, 2H, Ar–H-CO, H-3 and H-5), 7.18–7.14 (m, 3H, Ar–H), 7.08–7.06 (m, 2H, Ar–H), 6.89 (td, *J* = 8.4, 2.1 Hz, 1H, 4-F-Ar–H-CO, H-3), 6.73 (ddd, *J* = 10.1, 10.0, 2.2 Hz, 1H, 4-F-Ar–H-CO, H-5), 6.45 (d, *J* = 10.4 Hz, 1H, CH–N), 5.61 (d, *J* = 10.3 Hz, 1H, CH-Ph). MS m/z (%): 417.2 (7) [M^+^], 342.1 (15), 310.1 (15), 276.1 (50), 244.2 (100), 214.0 (35), 191.1 (10), 176.1 (33), 141.2 (100), 105.2 (100), 90.1 (25), 76.9 (100), 63.0 (43), 51.1 (33). Elem. anal. calcd. For C_24_H_17_F_2_N_3_O_2_ (417.2); C, 69.06; H, 4.11; N, 10.07. Found: C, 69.05; H, 4.10; N, 10.02.

###### 1-(2,4-dichlorophenyl)-3,4-diphenyl-2-(1H-1,2,4-triazol-1-yl) butane-1,4-dione (*10 k*)

Yield: 39%. M.P.: 178–181 °C; IR (KBr, cm^−1^): 3102.8 (C–H stretch, aromatic), 2931.3 (C-H, aliphatic), 1712.0, 1676.4 (C = O, ketone), 1584.3 (C = N), 1276.0 (C–N stretch, aromatic), 1135.7 (Ar-Cl). ^1^H-NMR (500 MHz, CDCl_3_) δ (ppm): 7.97 (s, 1H, triazole), 7.88 (s, 1H, triazole), 7.65 (d, *J* = 8.5 Hz, 1H, 2,4-diCl-Ar–H, H-6), 7.52 (t, 1H, Ar–H-CO, H-4), 7.49 (d, 2H, Ar–H-CO, H-2 and H-6), 7.42 (t, 2H, Ar–H-CO, H-3 and H-5), 7.32 (d, *J* = 1.7 Hz, 1H, 2,4-diCl-Ar–H, H-3), 7.22 (dd, *J* = 8.4, 1.7 Hz, 1H, 2,4-diCl-Ar–H, H-5), 7.18–7.16 (m, 3H, Ar–H), 7.12–7.09 (m, 2H, Ar–H), 6.70 (d, *J* = 10.3 Hz, 1H, CH–N), 5.57 (d, *J* = 10.3 Hz, 1H, CH-Ph). MS m/z (%): 449.1 (7) [M^+^], 344.0 (10), 276.0 (85), 241.1 (5), 172.9 (80), 145.0 (35), 105.2 (100), 90.0 (20), 77.0 (100), 51.1 (12). Elem. anal. calcd. For C_24_H_17_Cl_2_N_3_O_2_ (449.1); C, 64.01; H, 3.81; N, 9.33. Found: C, 63.98; H, 3.78; N, 9.26.

### In vitro cytotoxic assay

#### Chemicals

Fetal bovine serum (FBS) and RPMI-1640 medium were purchased from Gibco Invitrogen Co. (Scotland, UK) and sigma Aldrich, respectively. Dimethyl sulfoxide (DMSO), doxorubicin, penicillin, streptomycin was also purchased from Sigma Aldrich.

#### Cell cultures

Cell cultures were obtained from the human lung cancer cell line (A549), human cervical cancer cell line (Hela), human breast cancer cell line (MCF-7) and Normal cells isolated from human lung tissue (MRC-5) taken from the National Cell Bank of Iran (NCBI, Pasteur Institute, Tehran, Iran). All cells were cultured in RPMI-1640 medium supplemented with 10% FBS, antibiotics (penicillin 100 U/mL, streptomycin 100 lg/mL), at 37 ˚C in a humidified atmosphere containing 5% CO_2_.

#### MTT assay

Cytotoxic activities of all the synthesized compounds were assessed by standard 3-(4,5-dimethylthiazol-yl)-2,5-diphenyl-tetrazolium bromide (MTT) assay [53]. The assay was performed according to a known protocol [54–56]. The cells were seeded and plated in 96-well microplates at a density of 1 × 10^4^ cells per well in 180 μL complete culture media. After 24 h incubation, the cells were treated with 100 mL of different concentrations of synthesized compounds ranging from 1 × 10^–4^ to 1 × 10^–7^ M at identical conditions in triplicates. After 72 h, media were replaced with 150 μL media containing 0.5 mg/mL of MTT solution [57]. The plates were incubated at 37 °C for additional four hours. Then, media containing MTT were discarded and 150 μL dimethylsulfoxide (DMSO, > 99%) was added to each well to dissolve the formazan crystals. The absorbance in each well was determined at 570 nm by a BioRad microplate reader (Model 680) and then the IC_50_ values were calculated and demonstrated as mean ± SEM [58].

### Molecular docking study

Molecular docking studies were performed by AutoDock 4.2 program and AutoDock Tools 1.5.4 to investigate the binding mode of derivative in the active site of receptor [59]. The X-ray crystallographic structures of aromatase (PDB: 3EQM) (39) were obtained from the protein data bank (http://www.rcsb.org). Then, all water molecules and co-crystallized ligand were removed and hydrogen atoms were added to the protein and finally saved as pdbqt format. In the following, 3D structures of ligands were drawn and minimized under Molecular Mechanics MM^+^ and semi-empirical AM1 methods, using HyperChem software and saved as pdbqt format. The box dimensions were set at 65 × 65 × 65 with 0.375 Å grid spacing. Docking validation was done by re-docking the original ligand into the active site of receptor. Finally, conformations with the lowest free energies of binding were selected for analysis.

## Conclusion

In summary, we have designed and synthesized nineteen new 1,2,4-triazole-based derivatives starting from different phenyl halide analogues through three or four different steps. Their chemical structures were fully confirmed by IR, ^1^H-NMR, Mass spectra and elemental analysis. In vitro cytotoxic activity of the synthesized compounds were evaluated against three human cancer cell lines including MCF-7, Hela and A549, using MTT assay. The obtained results indicated that the synthesized compounds possessed relatively high to moderate antiproliferative activities against MCF-7 and Hela cancer cell lines. Compounds ***7d***, ***7e***, ***10a*** and ***10d*** were the most potent ones against three tested cell lines. Based on structure activity relationship (SAR) studies, it was found that the presence of electronegative substituents on the phenyl ring, as well as the presence of one-carbonyl group, resulted in a relative increase in the cytotoxic activity of the synthesized compounds. The outcome results relived that these active derivatives can be considered as a lead compound for anticancer treatments.

## Supplementary Information

**Additional file 1: Figures S1–S58**

## Data Availability

The data sets used and analyzed during the current study are available from the corresponding author on reasonable request. We have presented all data in the form of Tables and Figure. The PDB code (3EQM) was retrieved from protein data bank (www.rcsb.org). https://www.rcsb.org/structure/3EQM. A549 cells (ATCC No. CCL-185 human lung cancer cell line), Hela cells (ATCC No. CCL-2 human cervical cancer cell line), MCF-7 cells (ATCC No. HTB-22 human breast cancer cell line), MRC-5 cells (ATCC No. CCL-171 human lung tissue),. All the cell lines were purchased from the National Cell Bank of Iran (NCBI, Pasteur Institute, Tehran, Iran).

## Abbreviations

ADME: Adsorption Distribution, Metabolism and Discretion
RMSD: Root Mean Square Deviation
TPSA: Total polar surface area
MTT: (3-(4, 5 dimethylthiazol-yl)-2,5-diphenyl-tetrazolium bromide)
