# Supplementary material for: Design, synthesis and evaluation of novel 1,2,4-triazole derivatives as promising anticancer agents
Source: BMC Chem. 2022 Nov 12;16(1):91. doi: 10.1186/s13065-022-00887-x (PMC9652805; doi:10.1186/s13065-022-00887-x)
Supplement: Supplementary file 1 — Additional file 1: Figures S1–S58 [file 13065_2022_887_MOESM1_ESM.docx]

**Additional Data**

**Design, Synthesis and Evaluation of Novel 1,2,4-Triazole Derivatives as Promising Anticancer Agents**

Leila Emami^a1^, Sara Sadeghian^b1^, Ayyub Mojaddami^c,d1^, Soghra khabnadideh^a,b^, Amirhossein Sakhteman^b^, Hossein Sadeghpour^b^, Zeinab Faghih^a^, Masood Fereidoonnezhad^c^, Zahra Rezaei^a,b ^[[1]](#footnote-1)^*^

*^a^Pharmaceutical Sciences Research center, School of Pharmacy, Shiraz University of Medical*

*Sciences, Shiraz, Iran.*

*^b^Department of Medicinal Chemistry, School of Pharmacy, Shiraz University of Medical Sciences, Shiraz, Iran*

*^c^Department of Medicinal chemistry, School of Pharmacy,*[*Ahvaz Jundishapur University of Medical Sciences, Ahvaz*](https://scholar.google.com/citations?view_op=view_org&hl=en&org=2368880481777056914)*, Iran.*

*^d^Toxicology Research Center, Medical Basic Sciences Research Institute, Ahvaz Jundishapur University of Medical Sciences, Ahvaz, Iran*


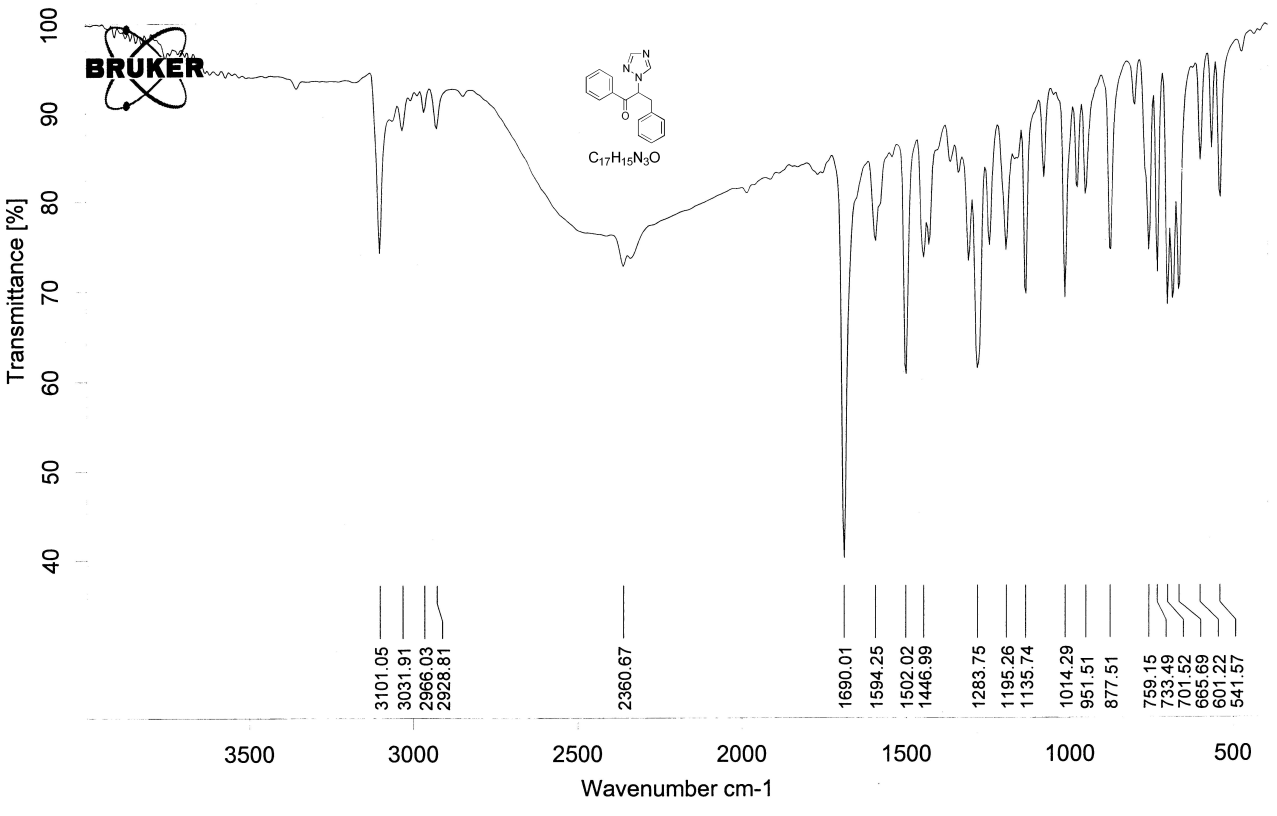


**Figure 1.** IR spectrum of compound **7a**.


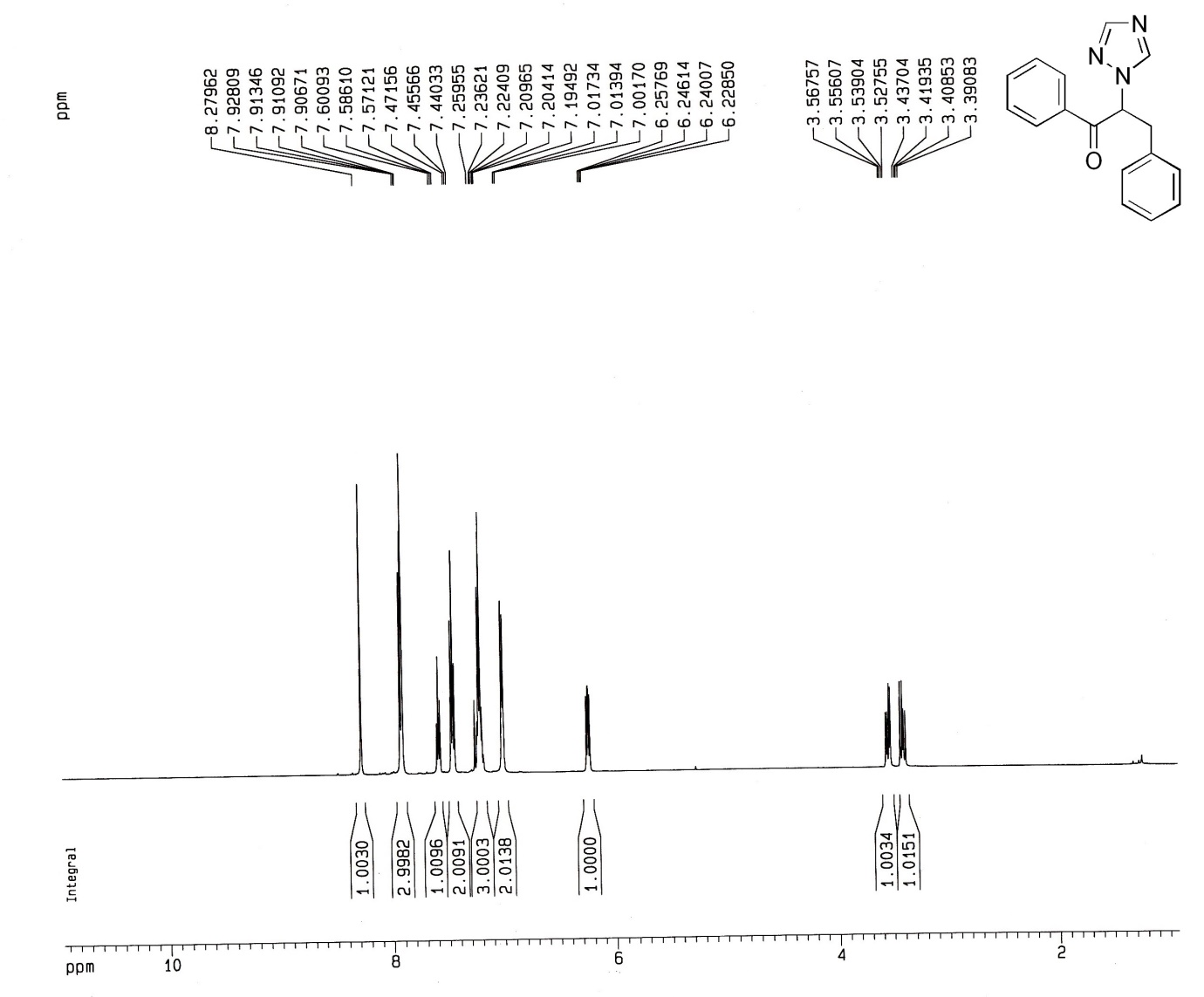


**Figure 2.** ^1^HNMR spectrum of compound **7a.**


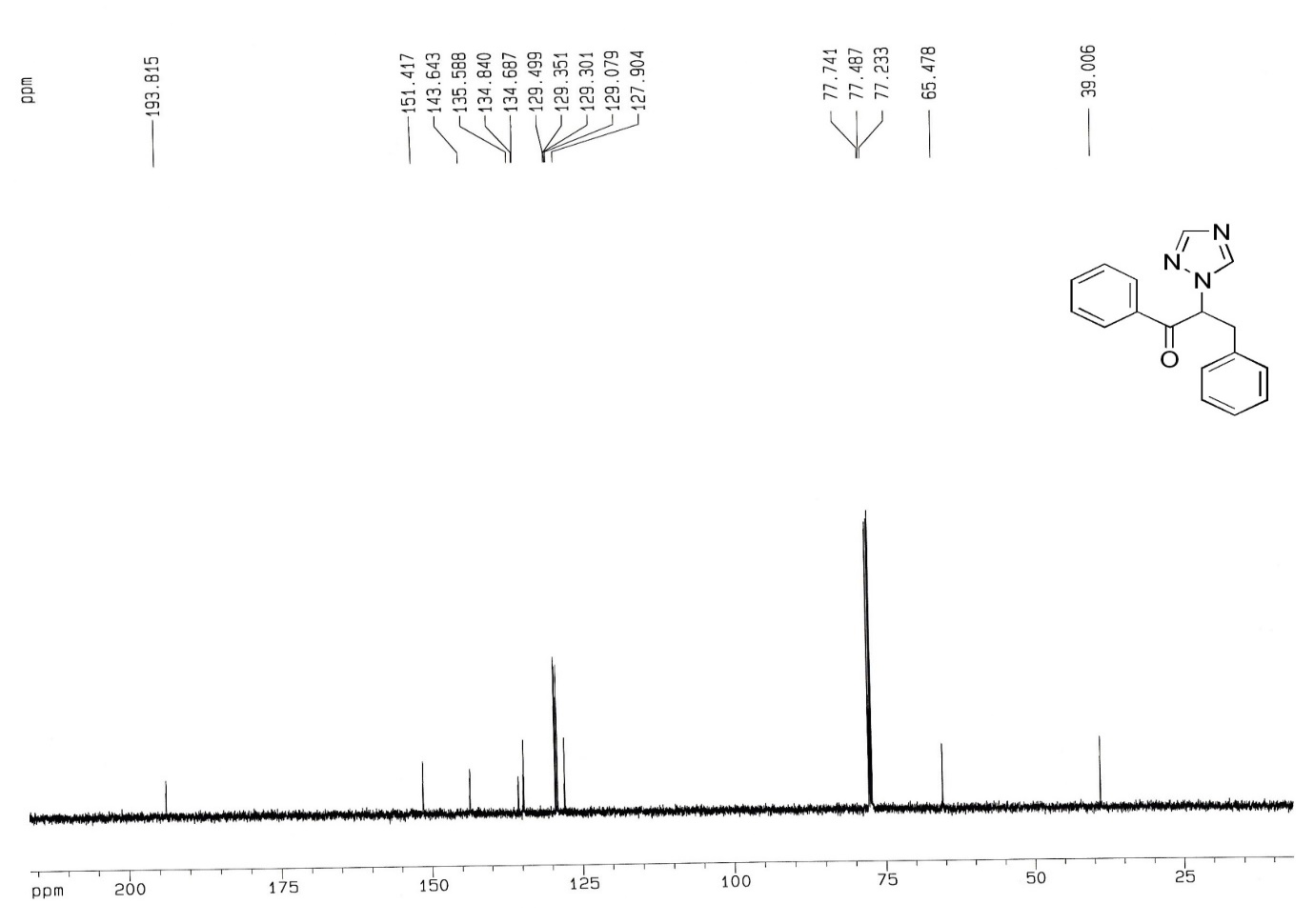


**Figure 3.** ^13^CNMR spectrum of compound **7a.**


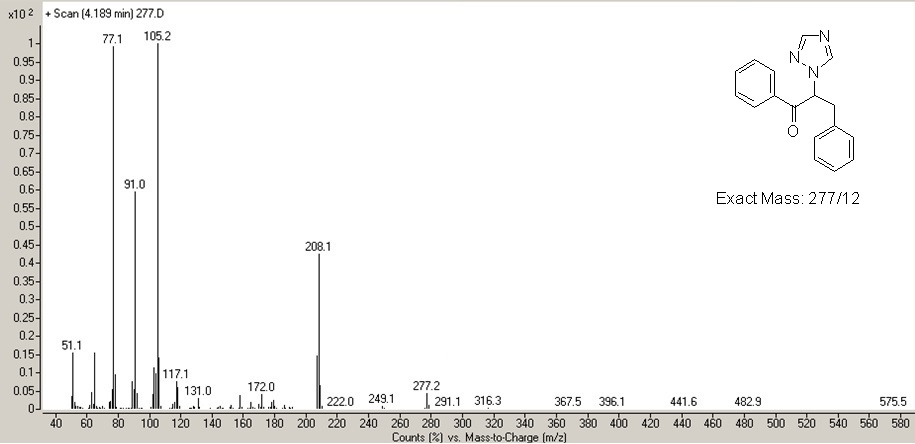


**Figure 4.** Mass spectrum of compound **7a**.


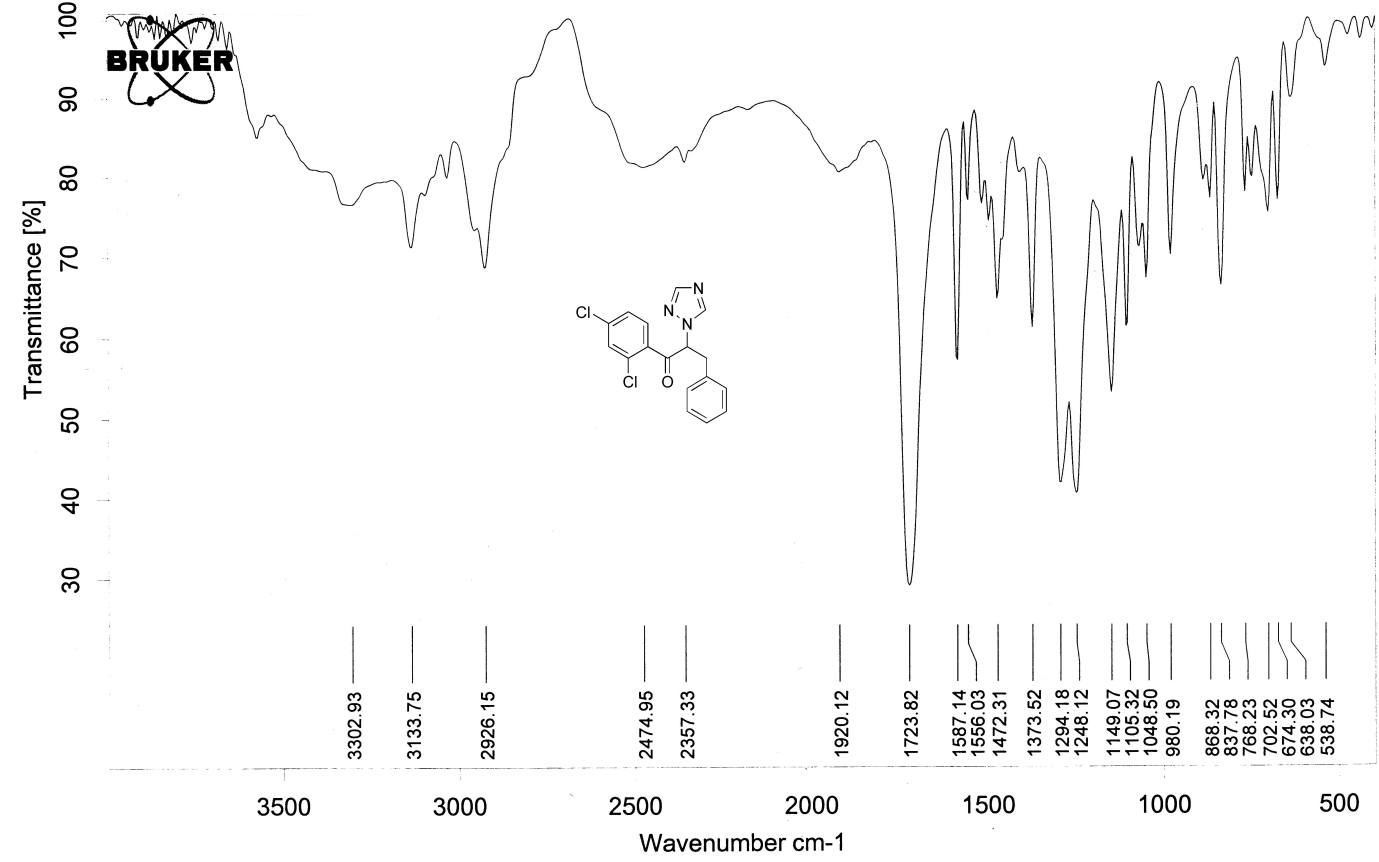


**Figure 5.** IR spectrum of compound **7b.**


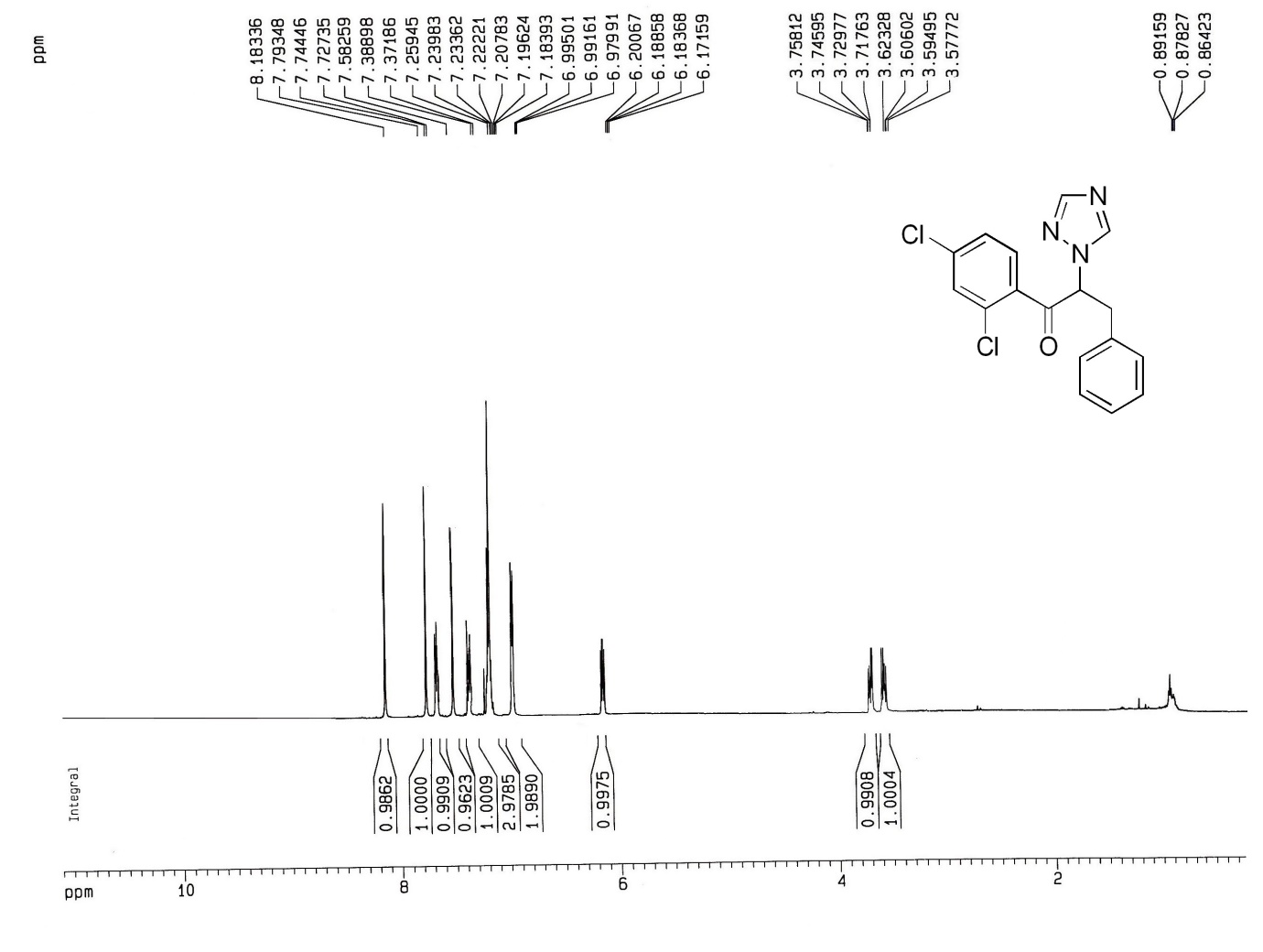


**Figure 6.** ^1^HNMR spectrum of compound **7b.**


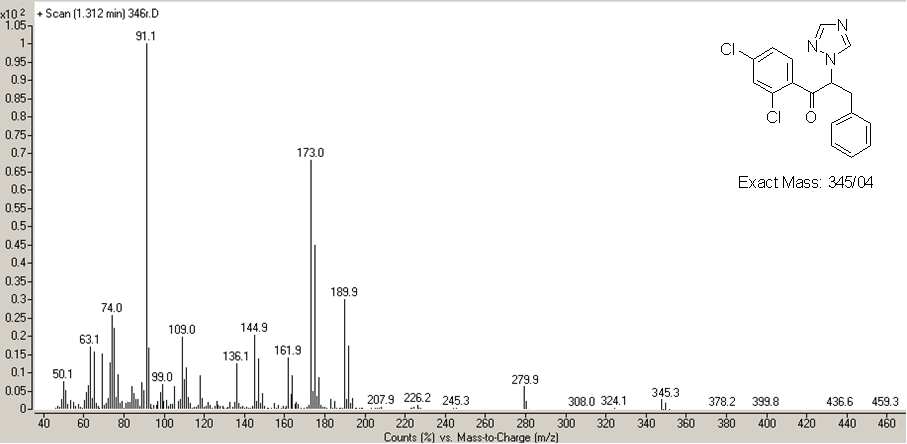


**Figure 7.** Mass spectrum of compound **7b.**


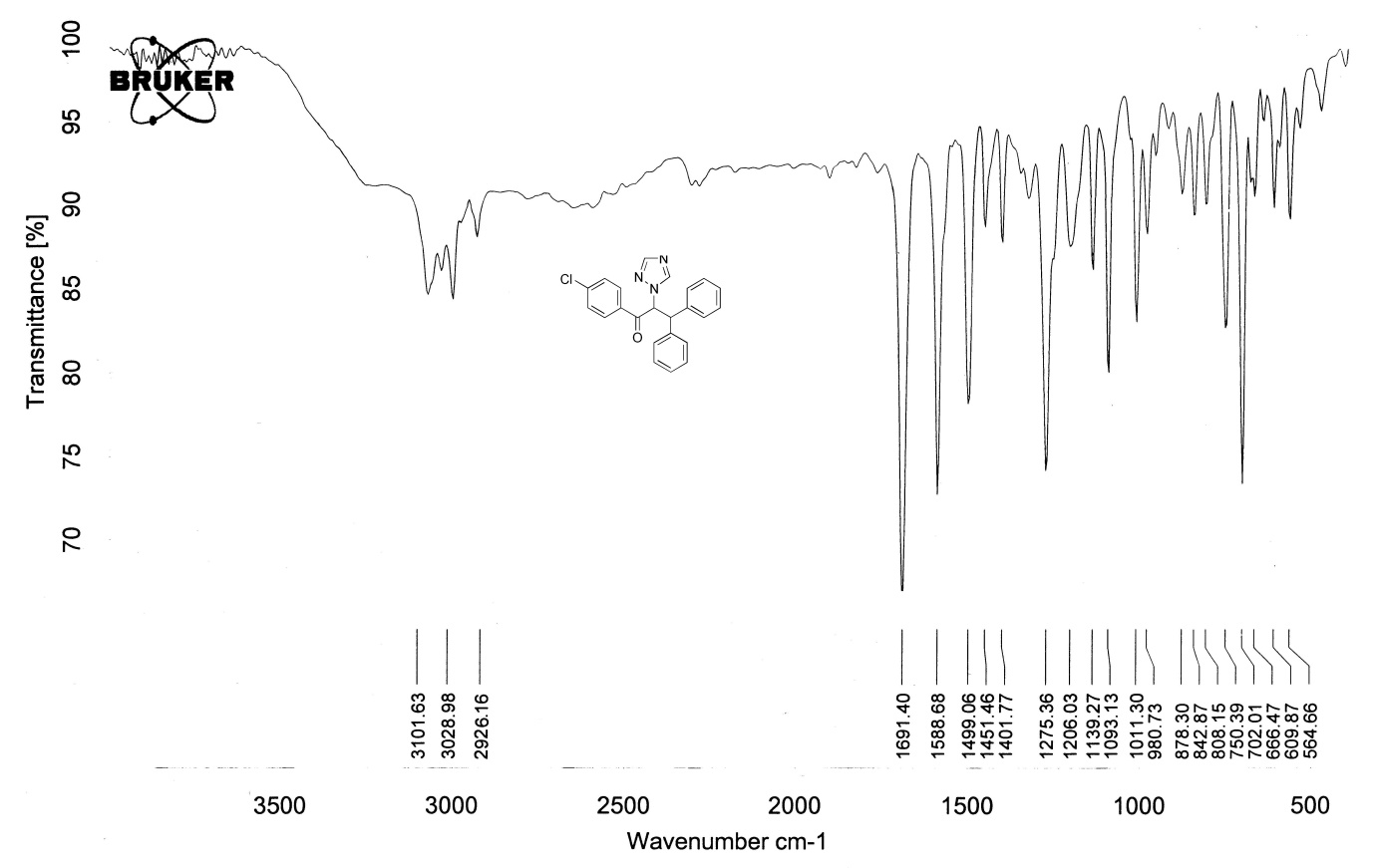


**Figure 8.** IR spectrum of compound **7c.**


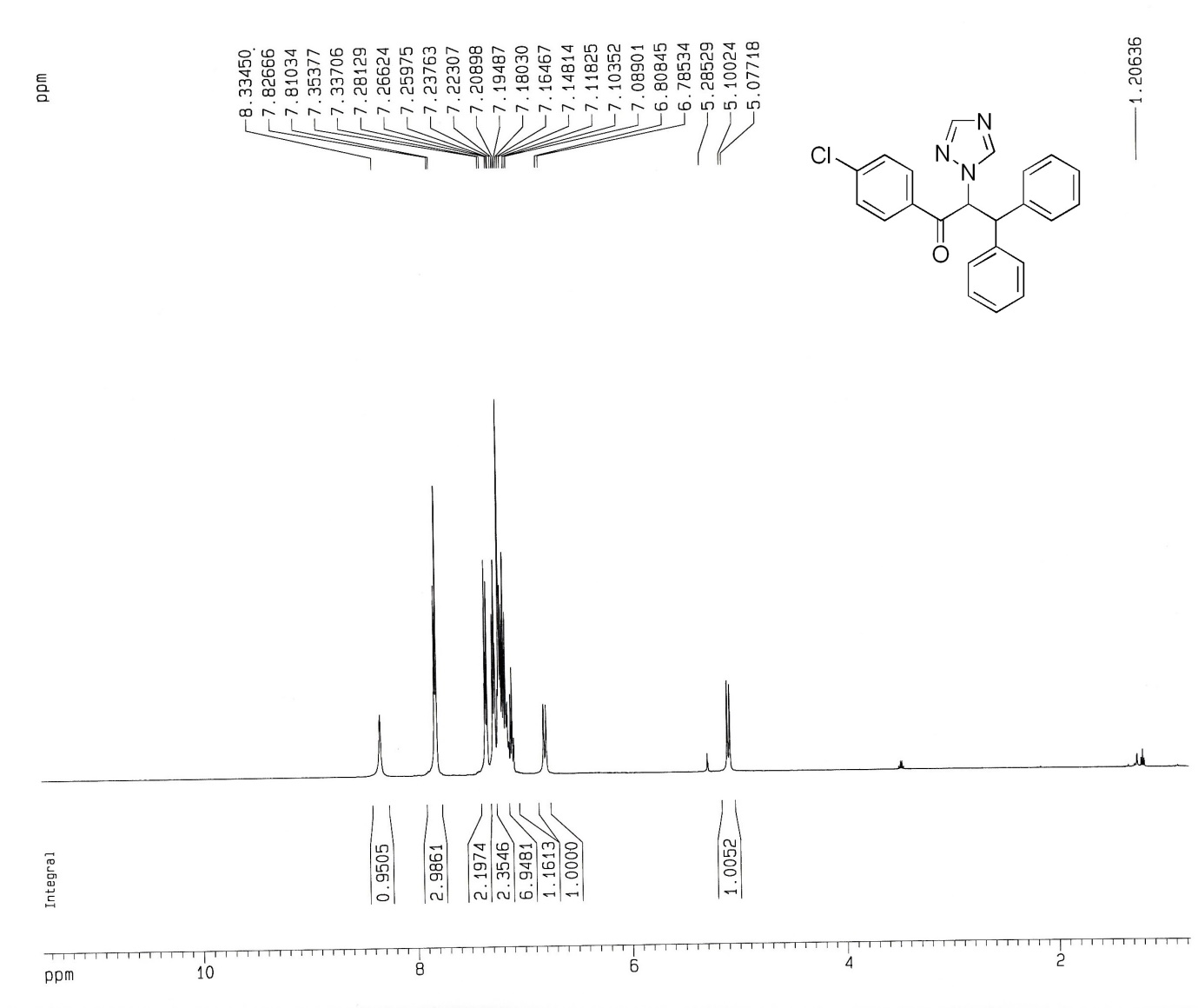


**Figure 9.** ^1^HNMR spectrum of compound **7c.**


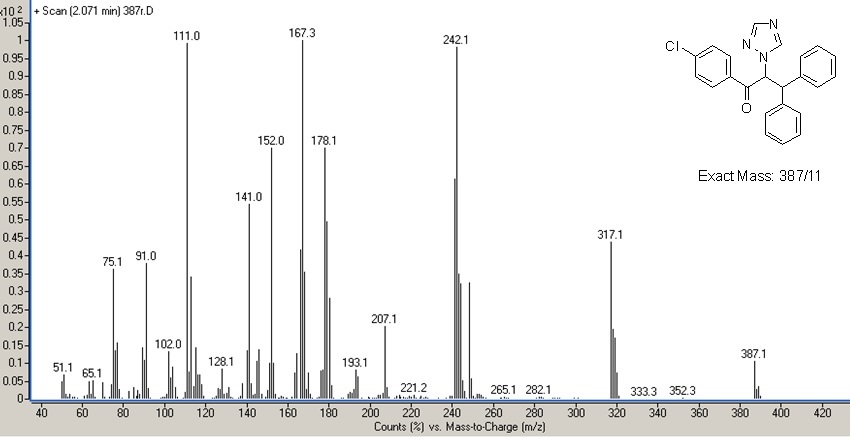


**Figure 10.** Mass spectrum of compound **7c.**


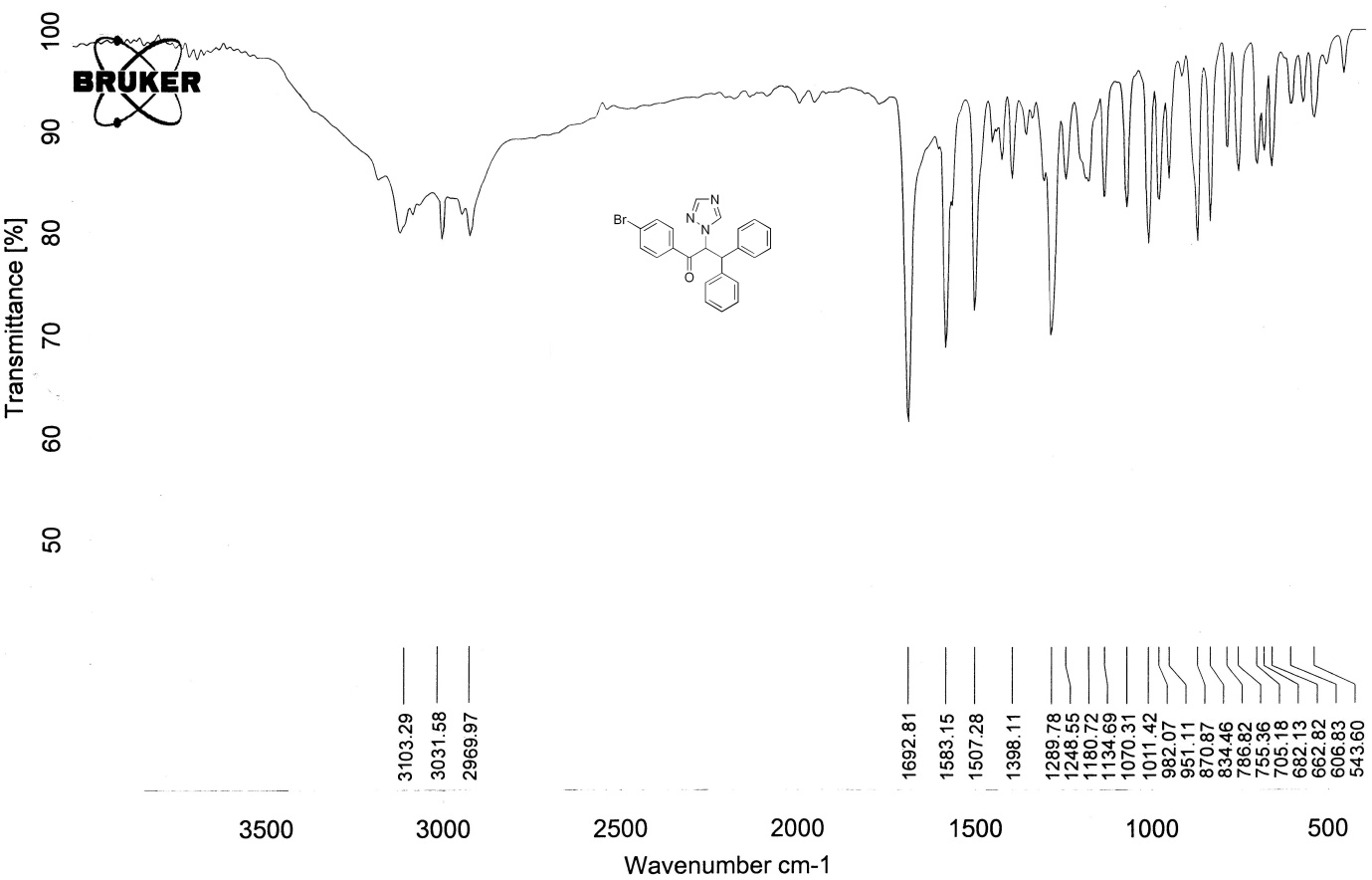


**Figure 11**. IR spectrum of compound **7d.**


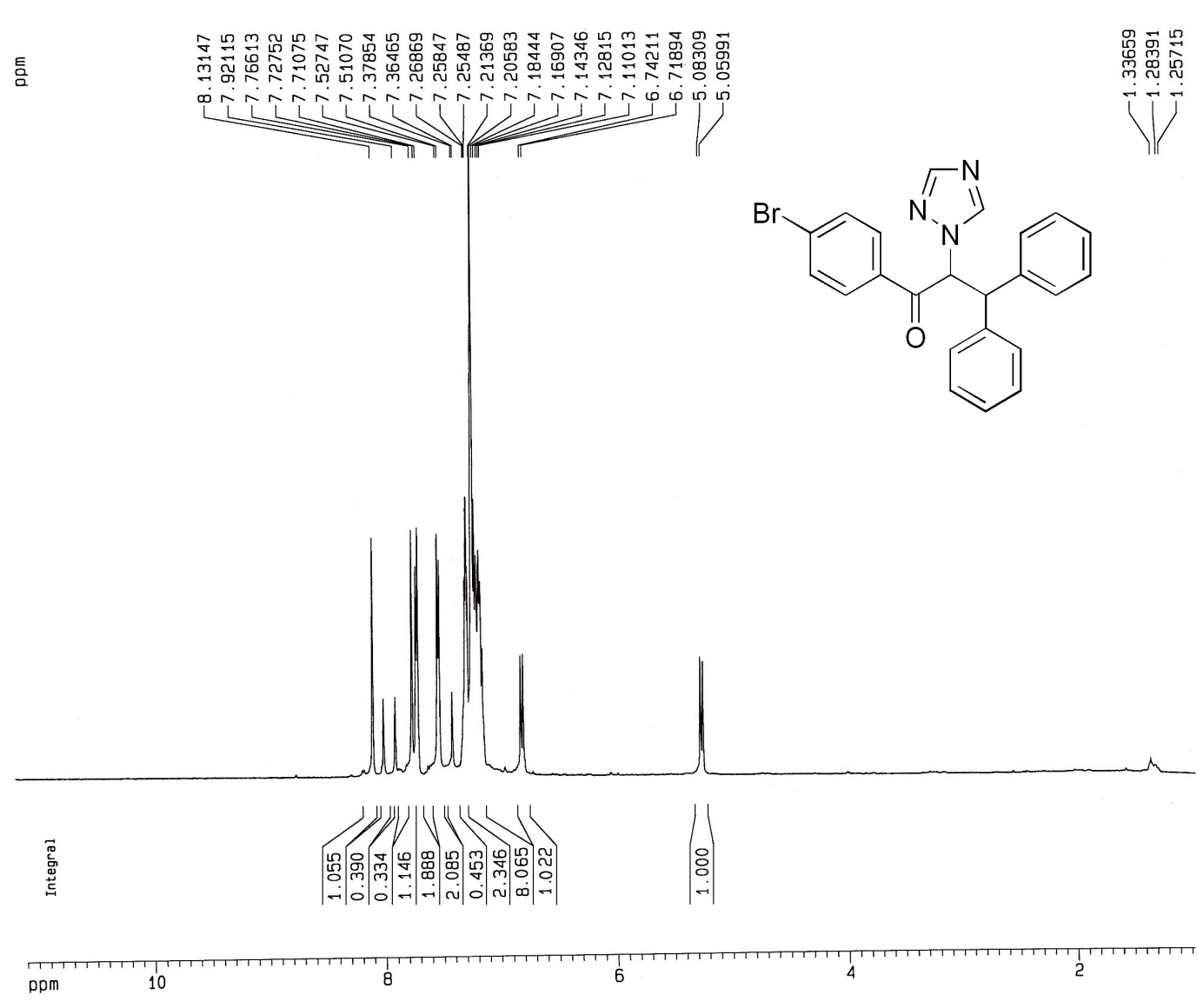


**Figure 12.** ^1^HNMR spectrum of compound **7d.**


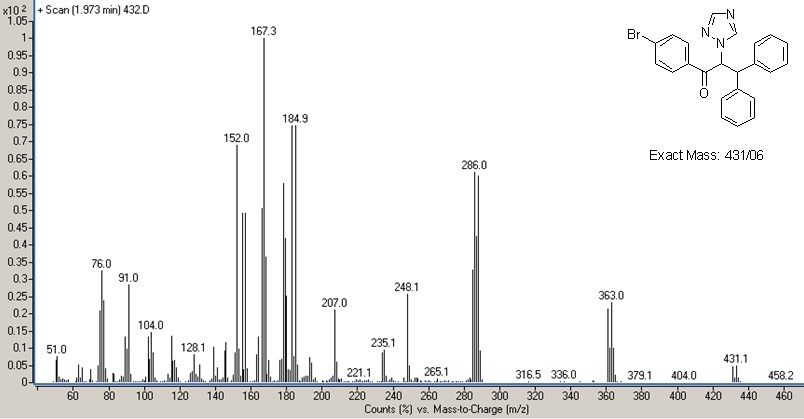


**Figure 13.** Mass spectrum of compound **7d.**


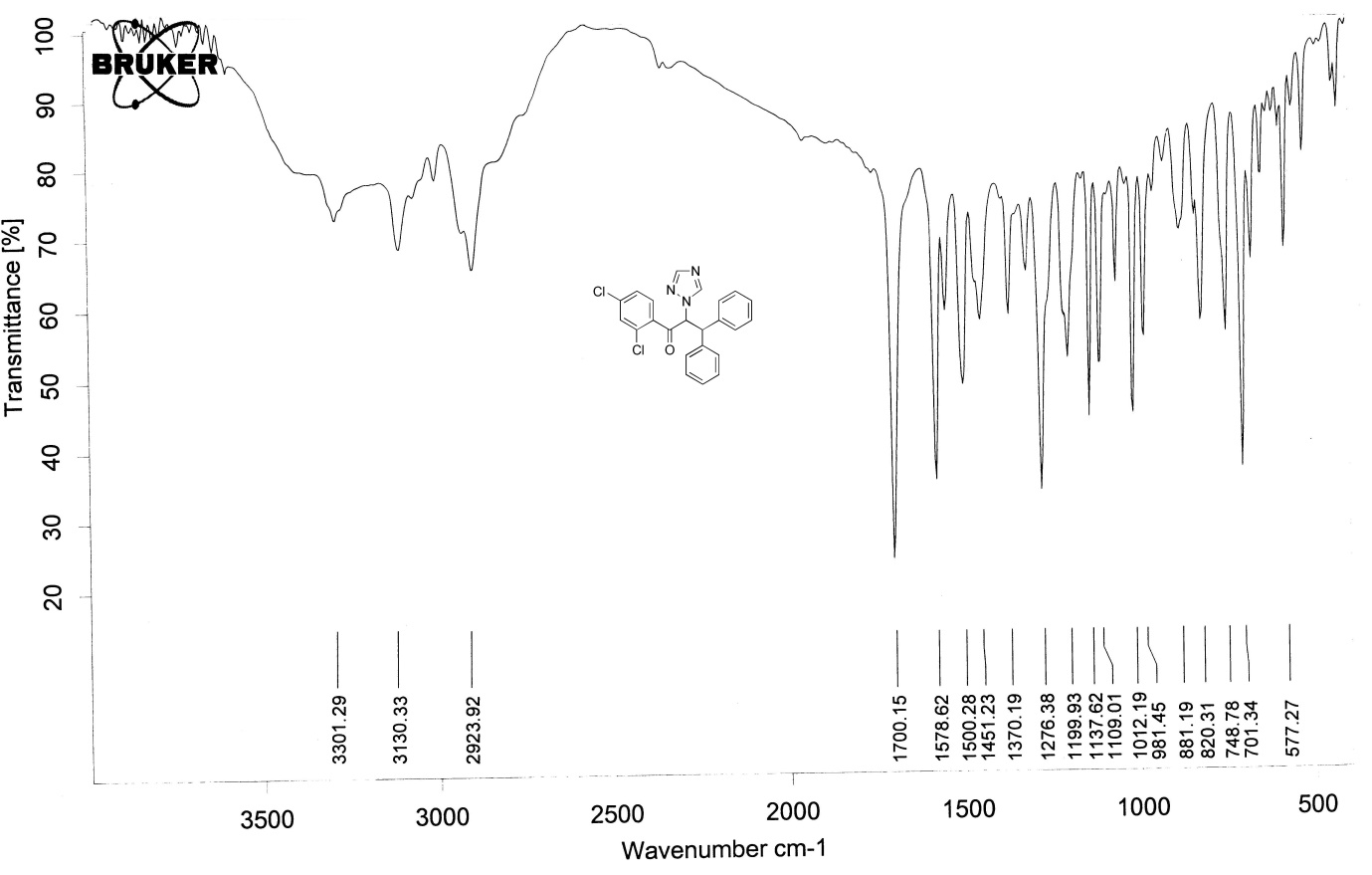


**Figure 14.** IR spectrum of compound **7e.**


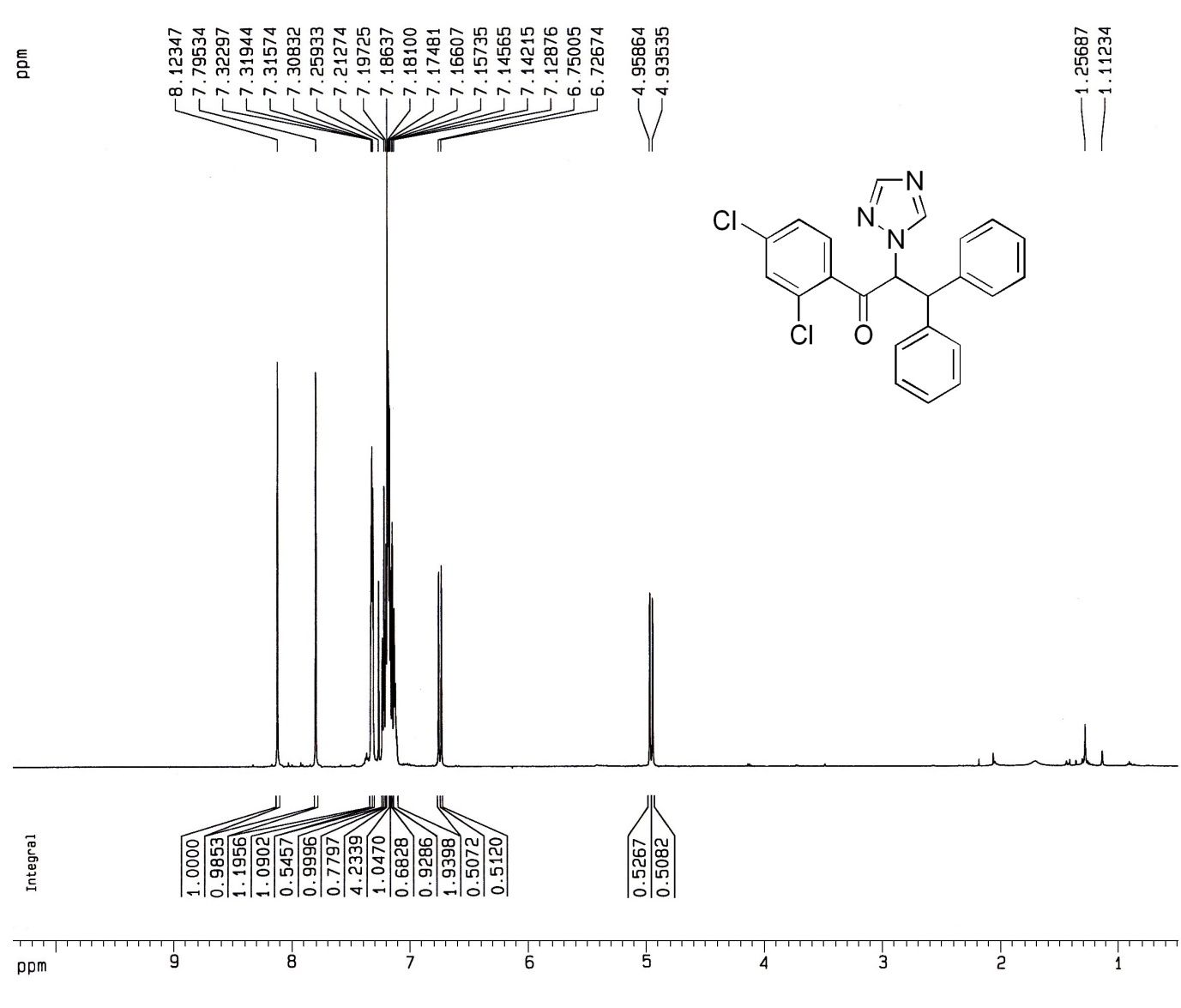


**Figure 15.** ^1^HNMR spectrum of compound **7e.**


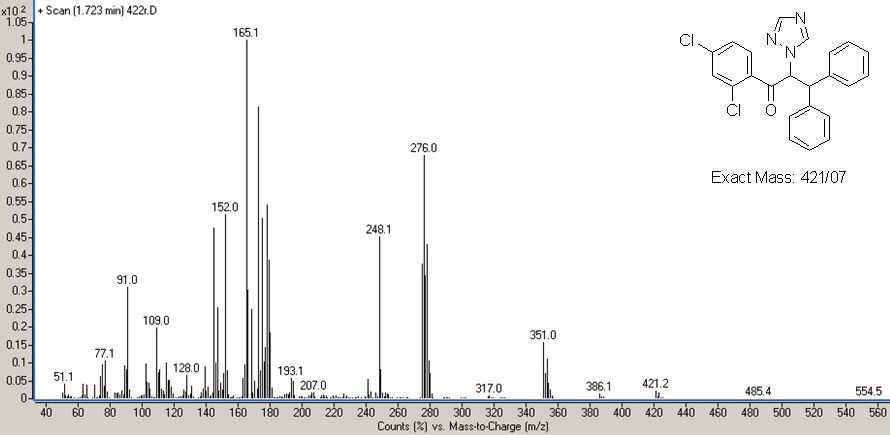


**Figure 16.** Mass spectrum of compound **7e.**


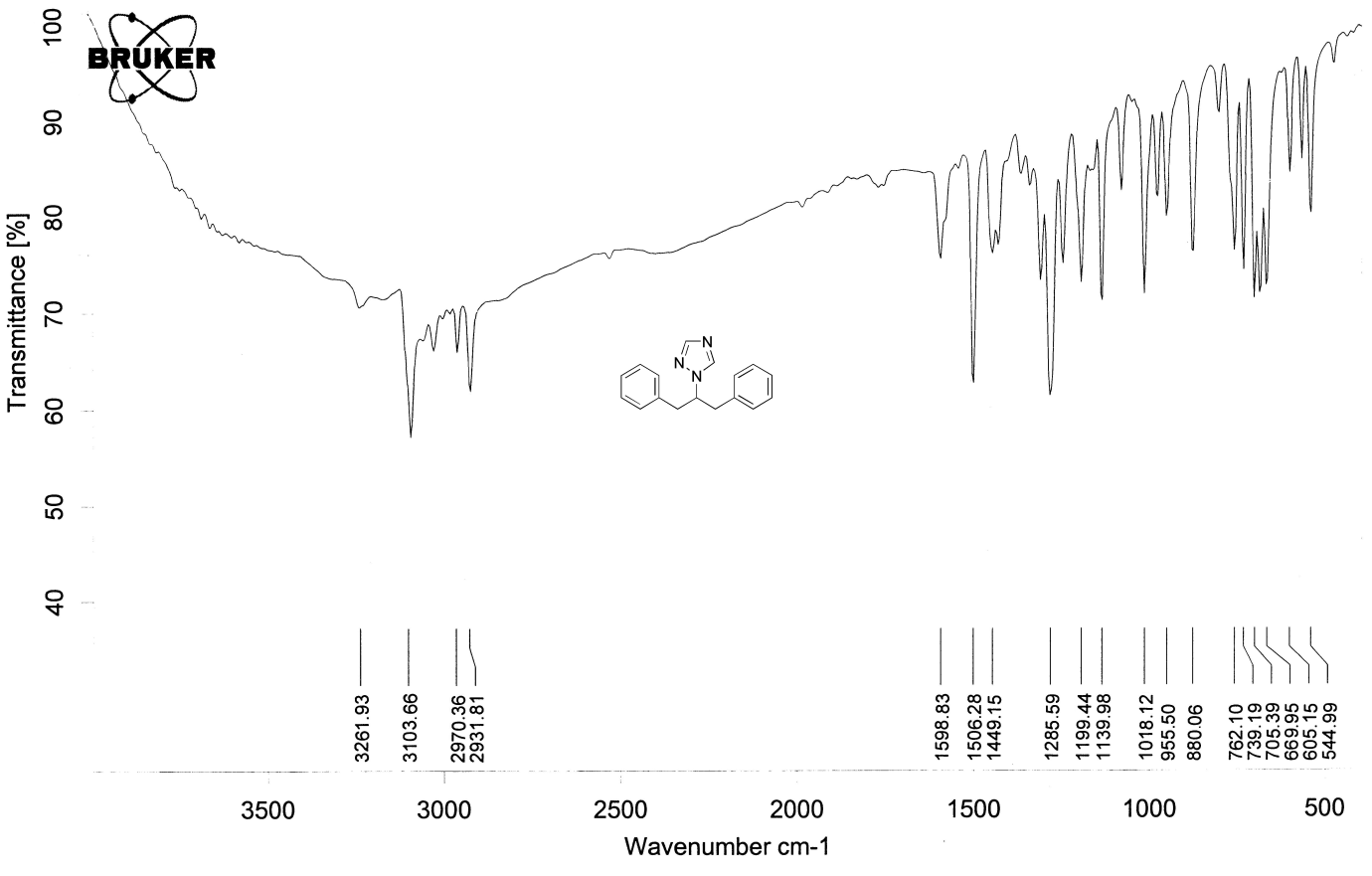


**Figure 17.** IR spectrum of compound **8a.**


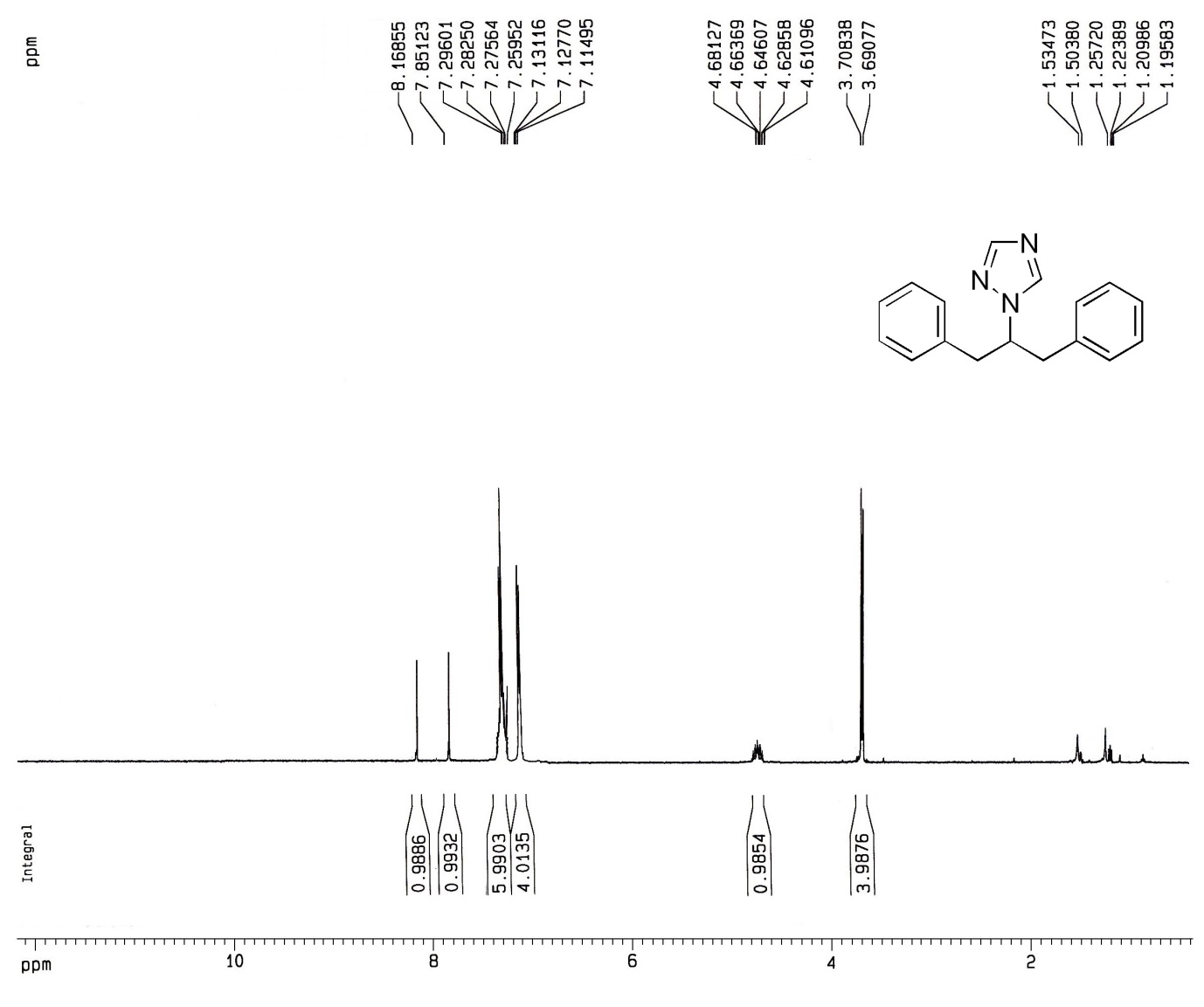


**Figure 18.** ^1^HNMR spectrum of compound **8a.**


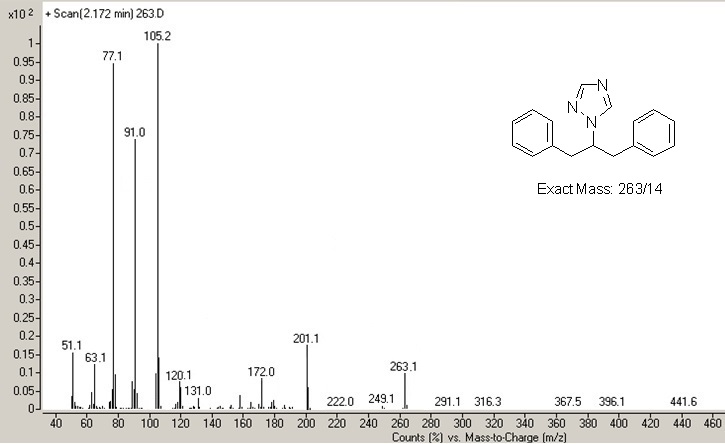


**Figure 19.** Mass spectrum of compound **8a.**


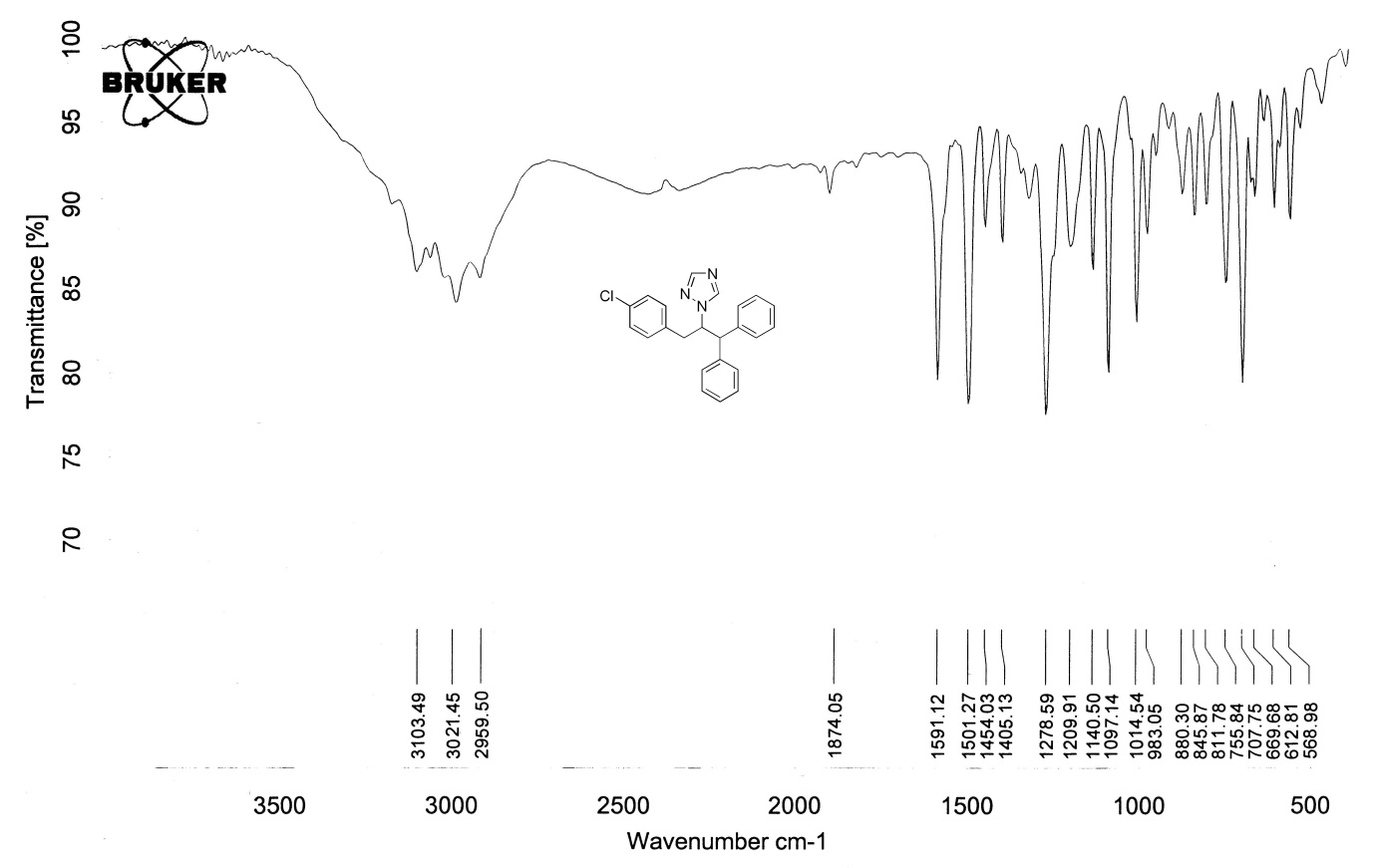


**Figure 20.** IR spectrum of compound **8b.**


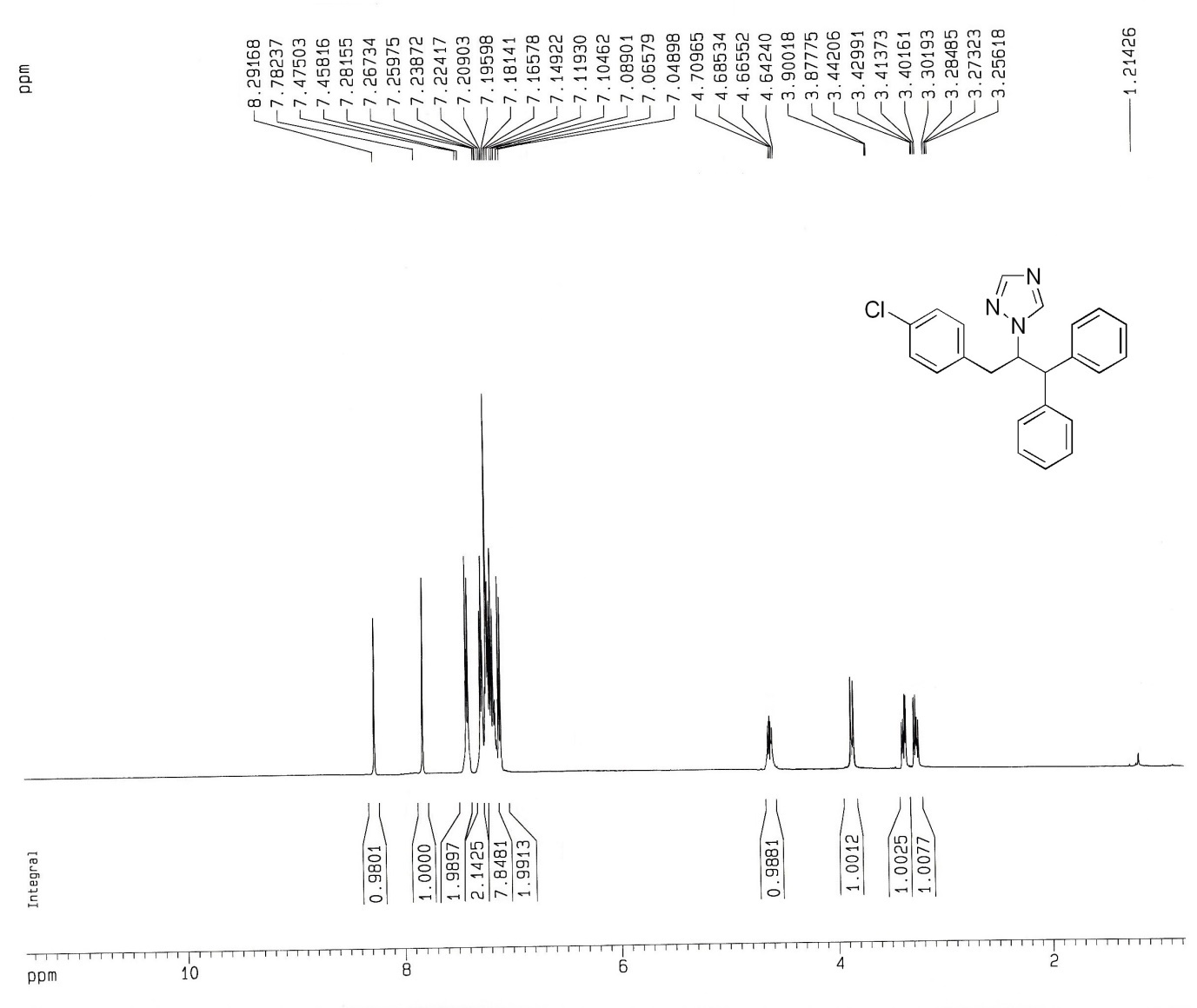


**Figure 21.** ^1^HNMR spectrum of compound **8b.**


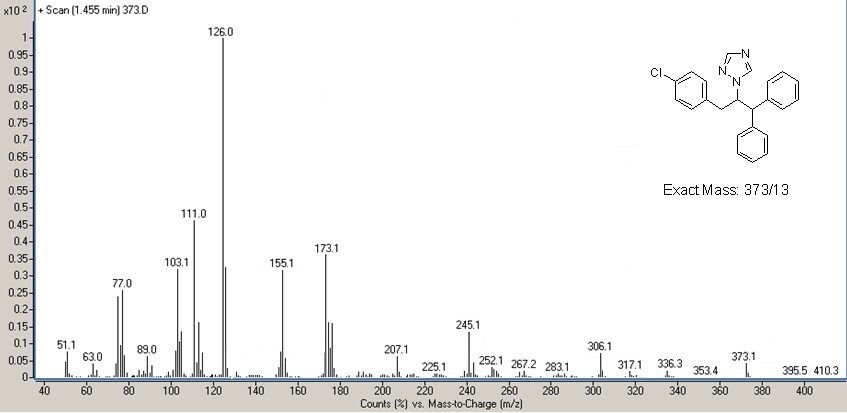


**Figure 22.** Mass spectrum of compound **8b.**


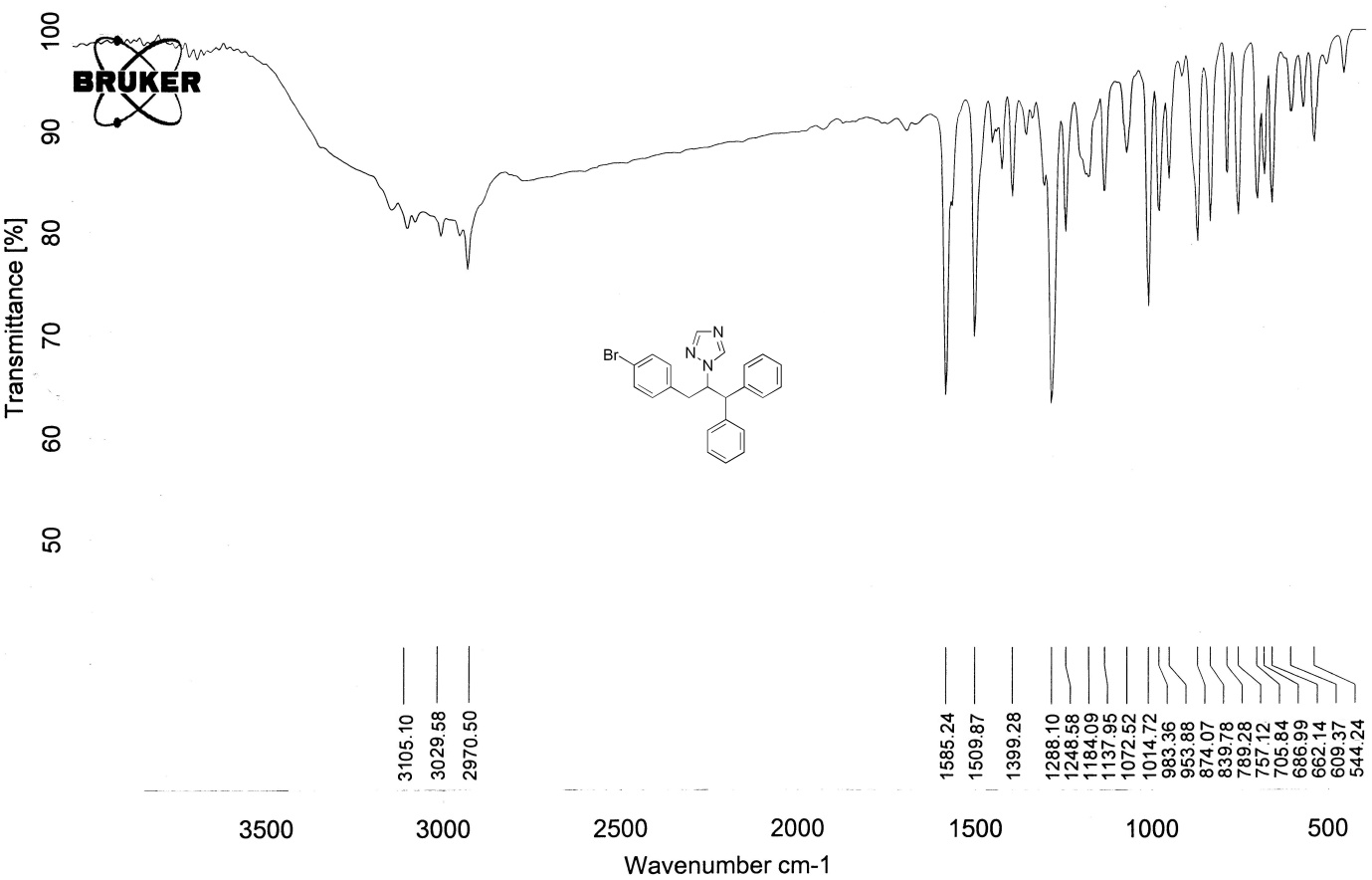


**Figure 23.** IR spectrum of compound **8c.**


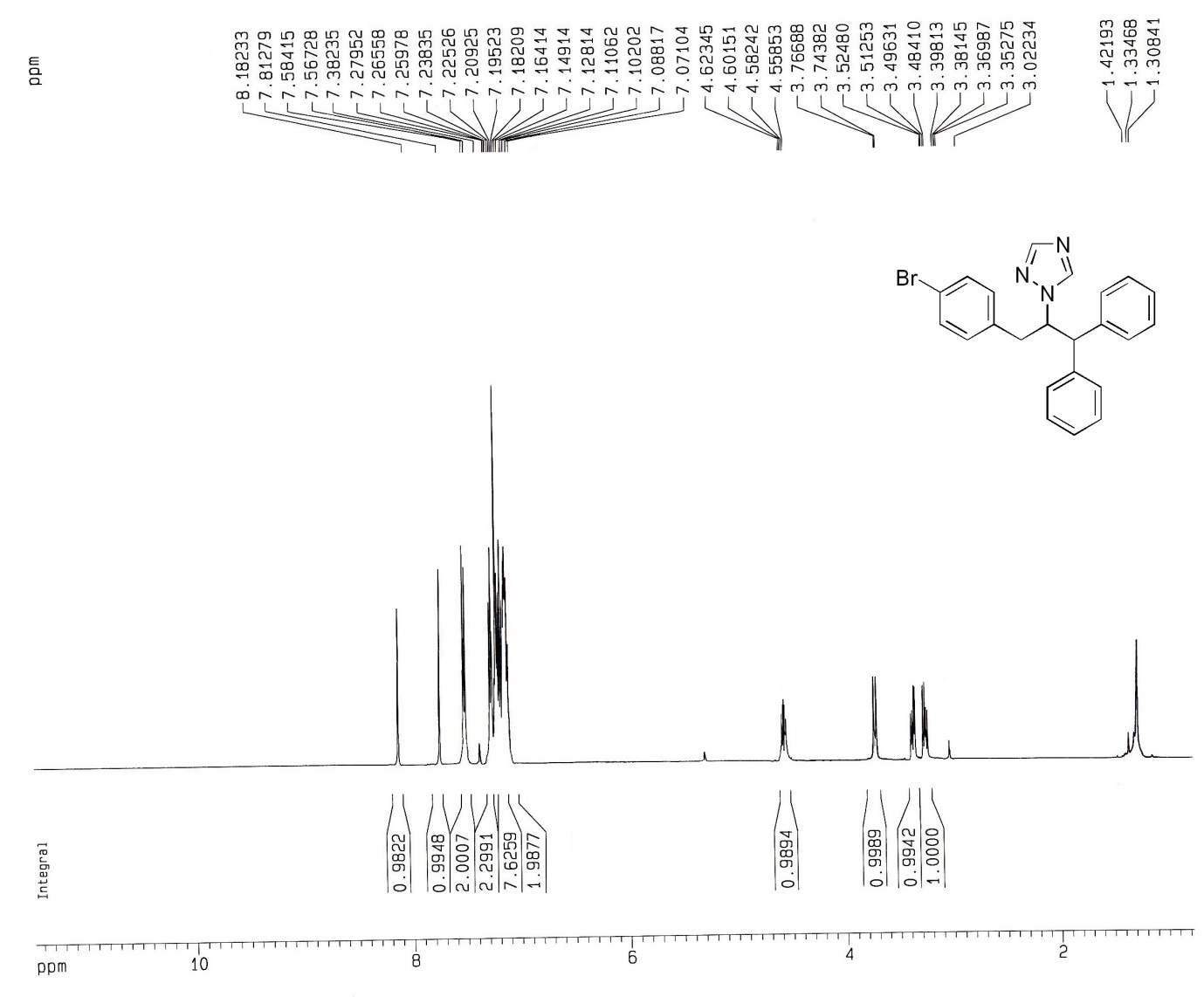


**Figure 24.** ^1^HNMR spectrum of compound **8c.**


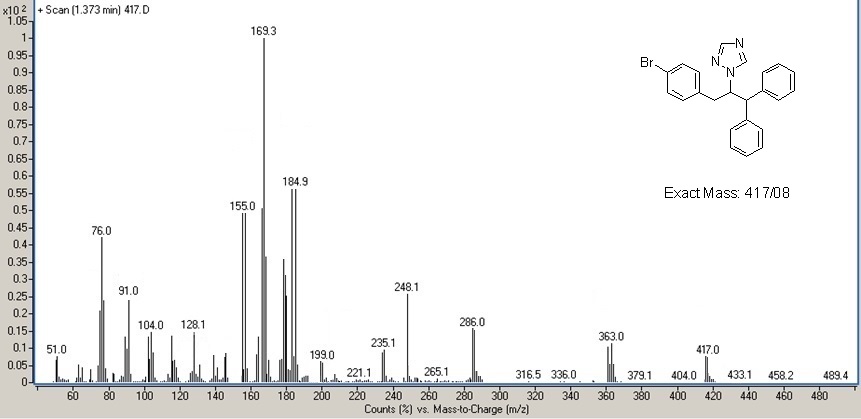


**Figure 25.** Mass spectrum of compound **8c.**


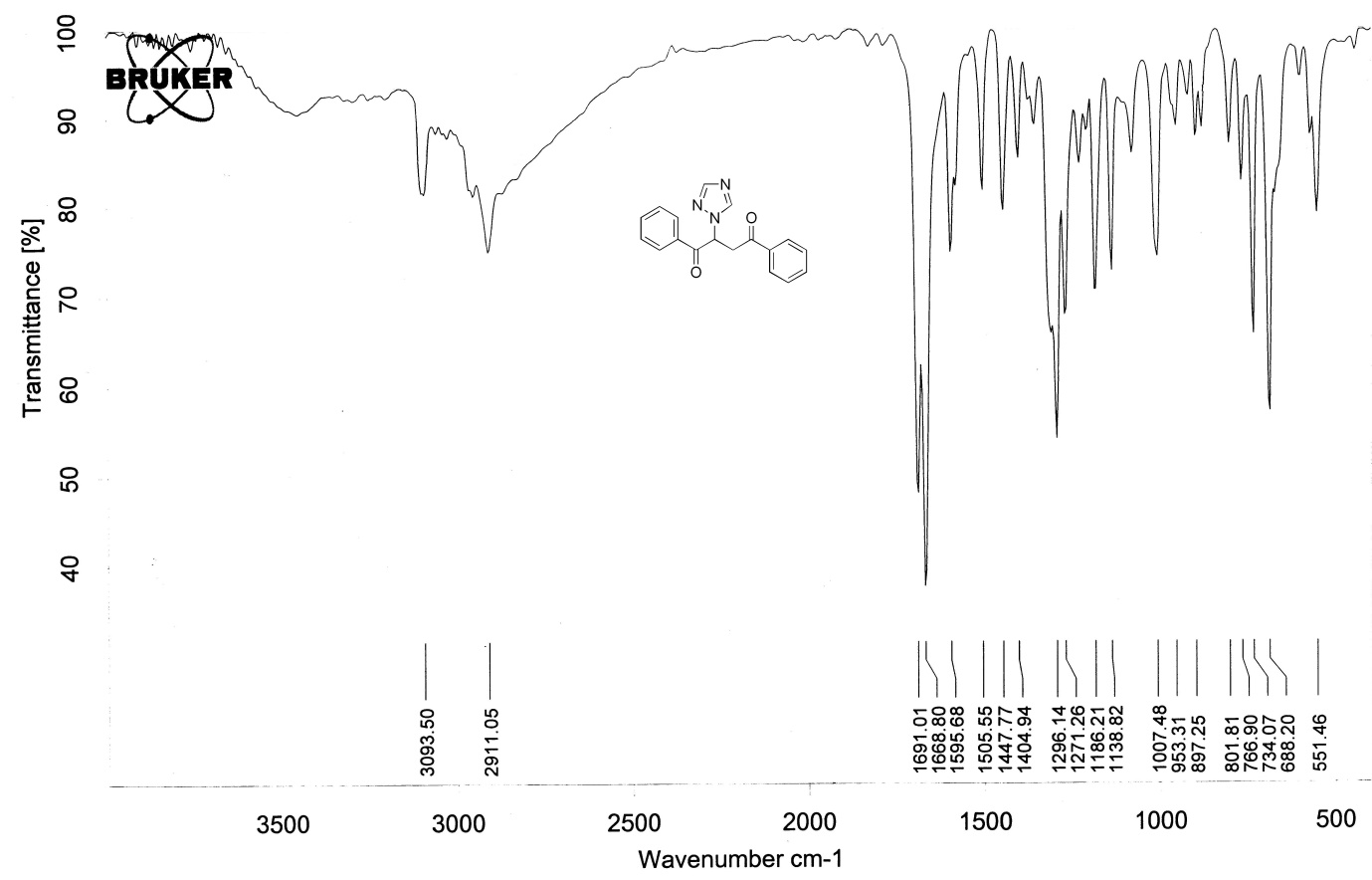


**Figure 26.** IR spectrum of compound **10a.**


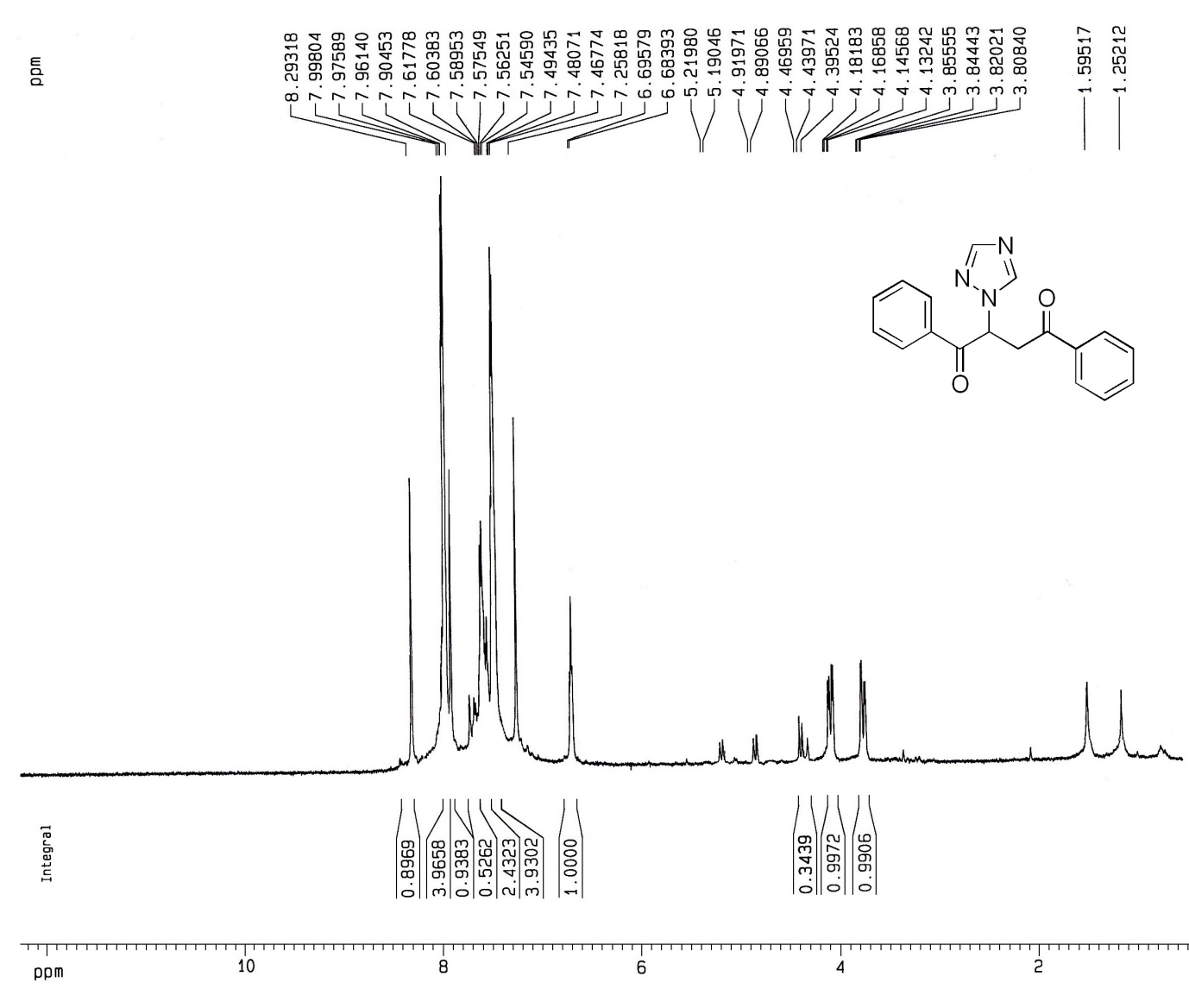


**Figure 27.** ^1^HNMR spectrum of compound **10a.**


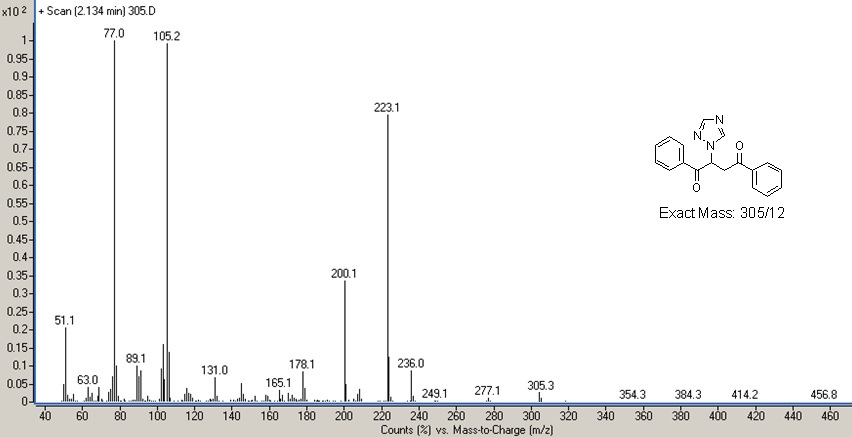


**Figure 28.** Mass spectrum of compound **10a.**


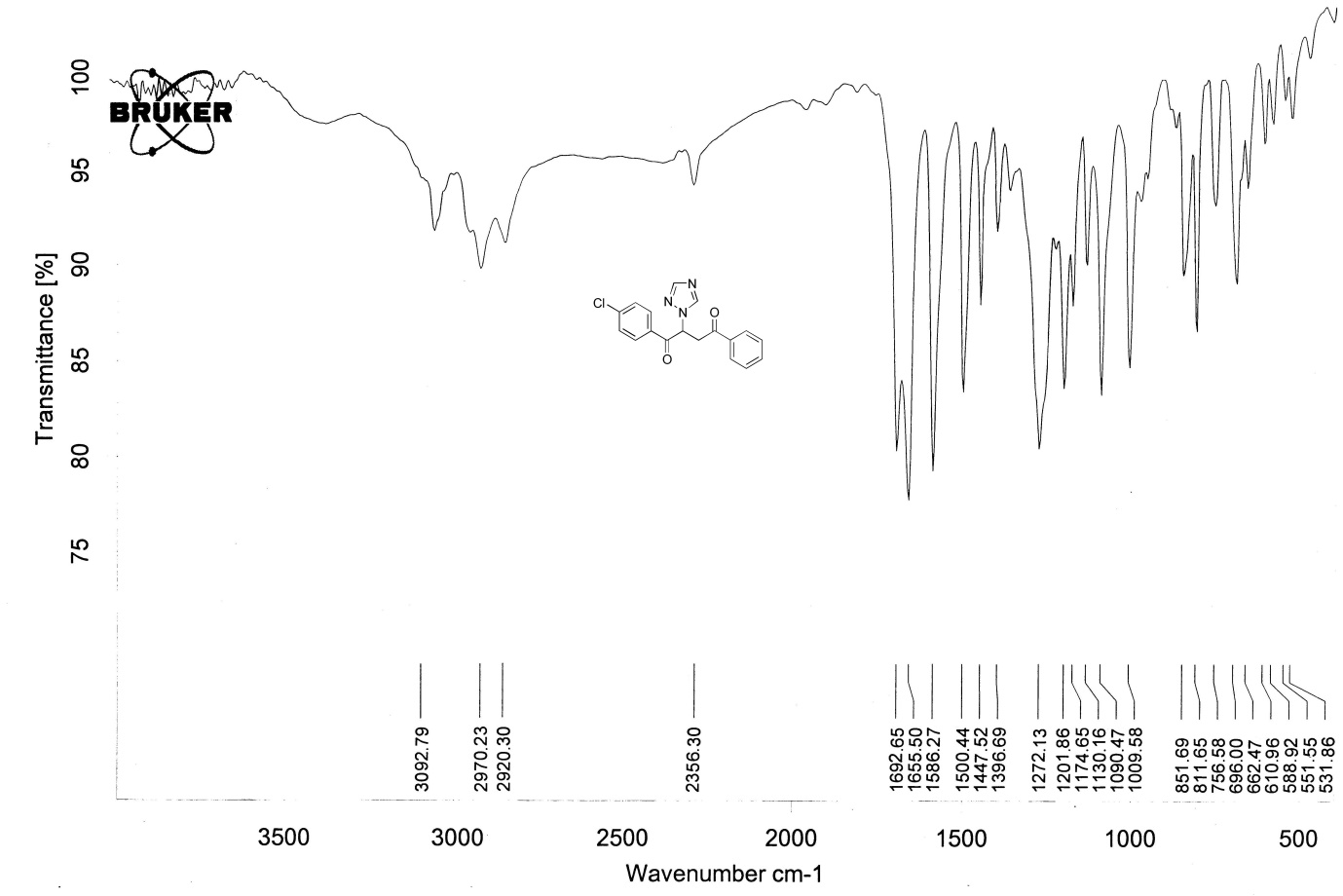


**Figure 29.** IR spectrum of compound **10b.**


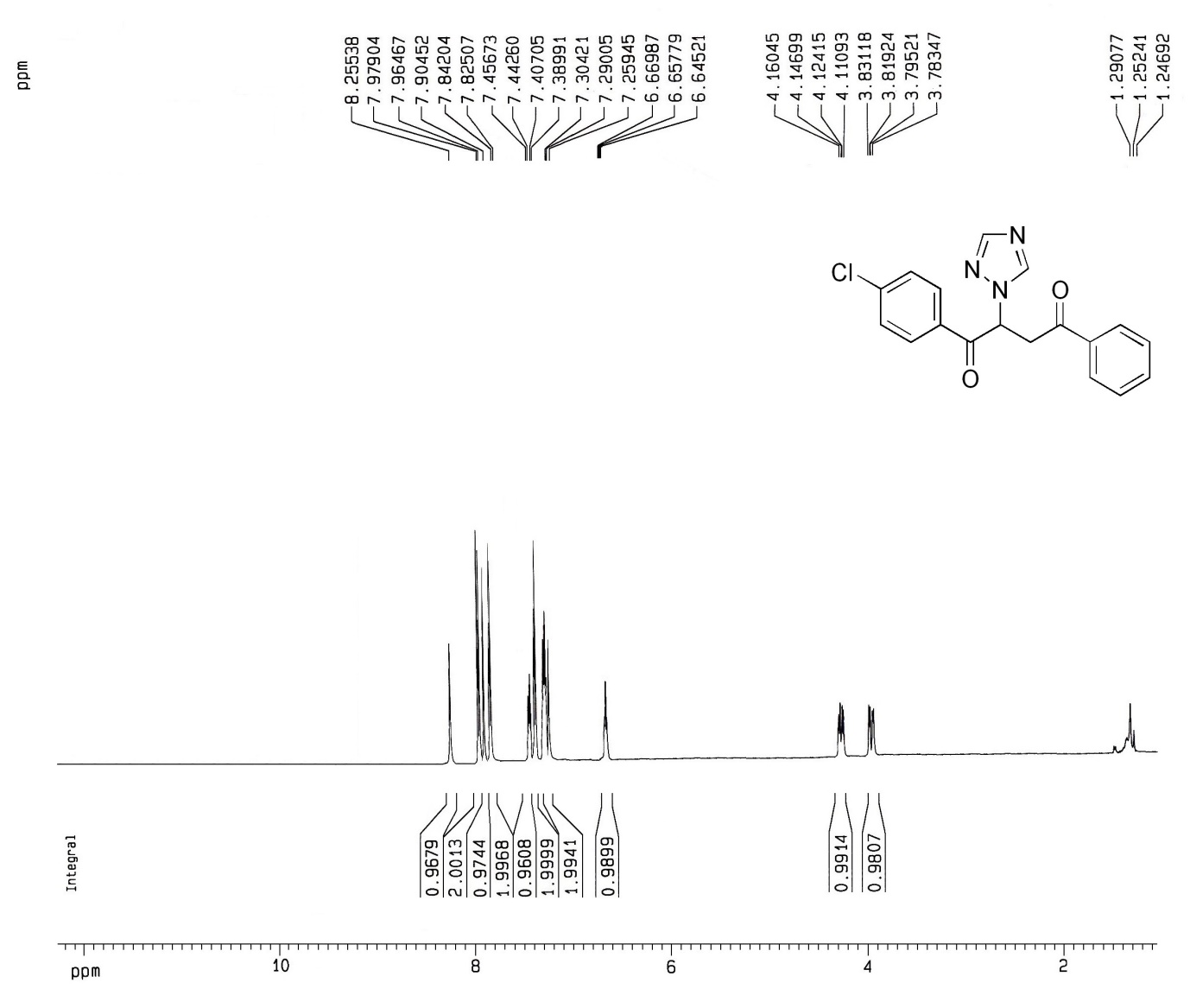


**Figure 30.** ^1^HNMR spectrum of compound **10b.**


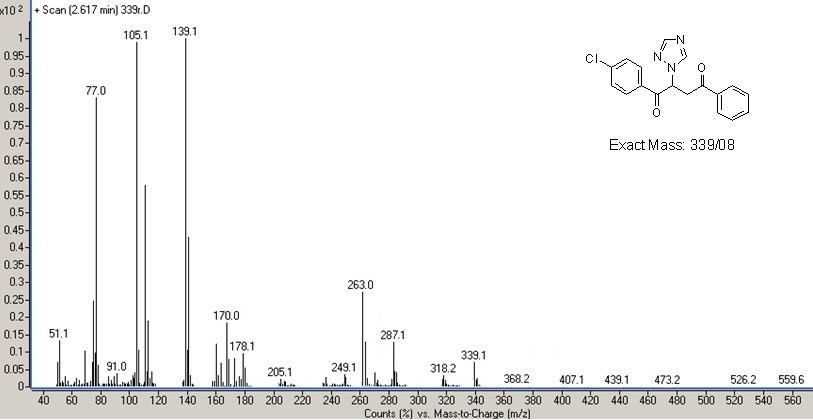


**Figure 31.** Mass spectrum of compound **10b.**


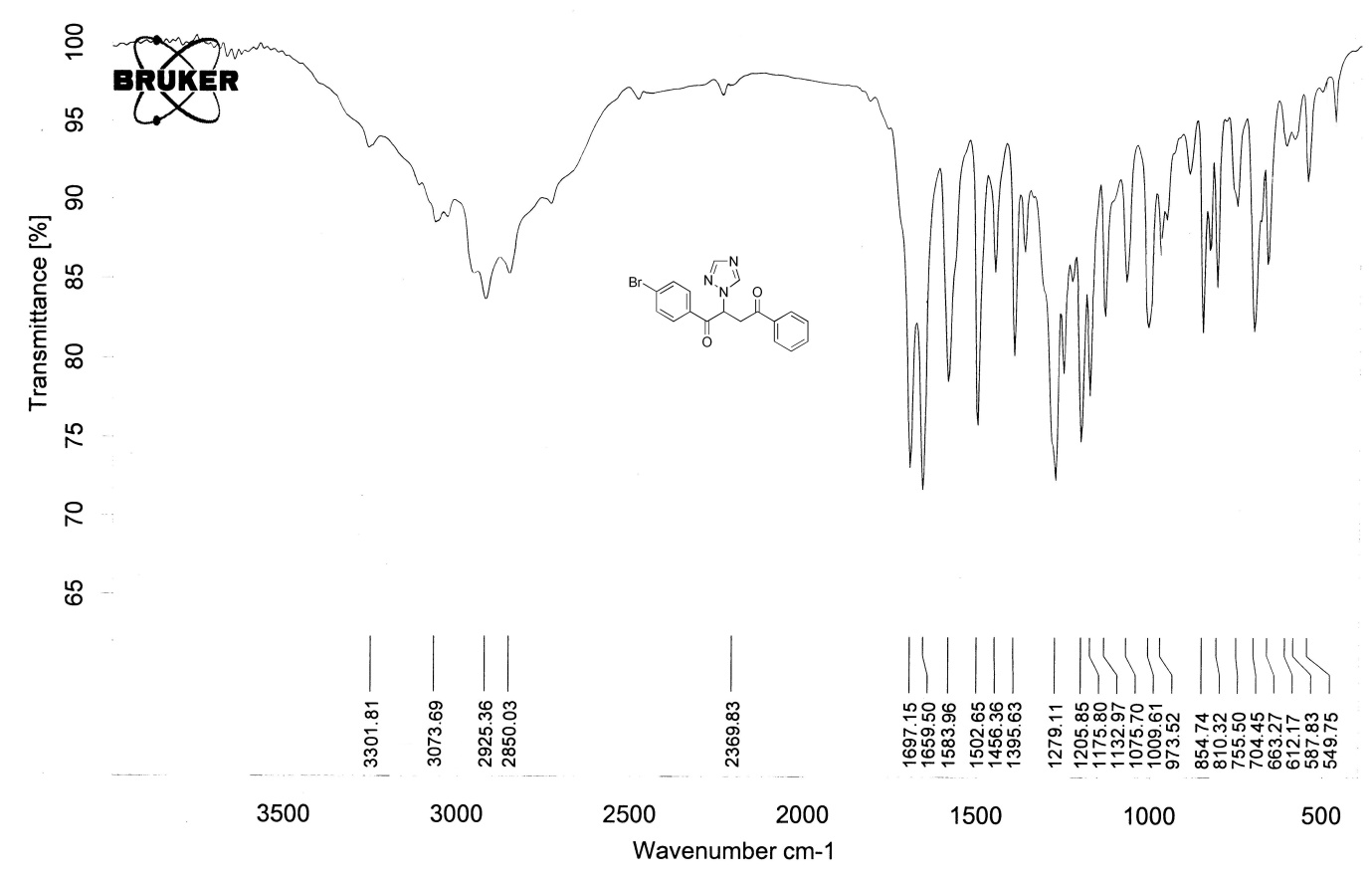


**Figure 32.** IR spectrum of compound **10c.**


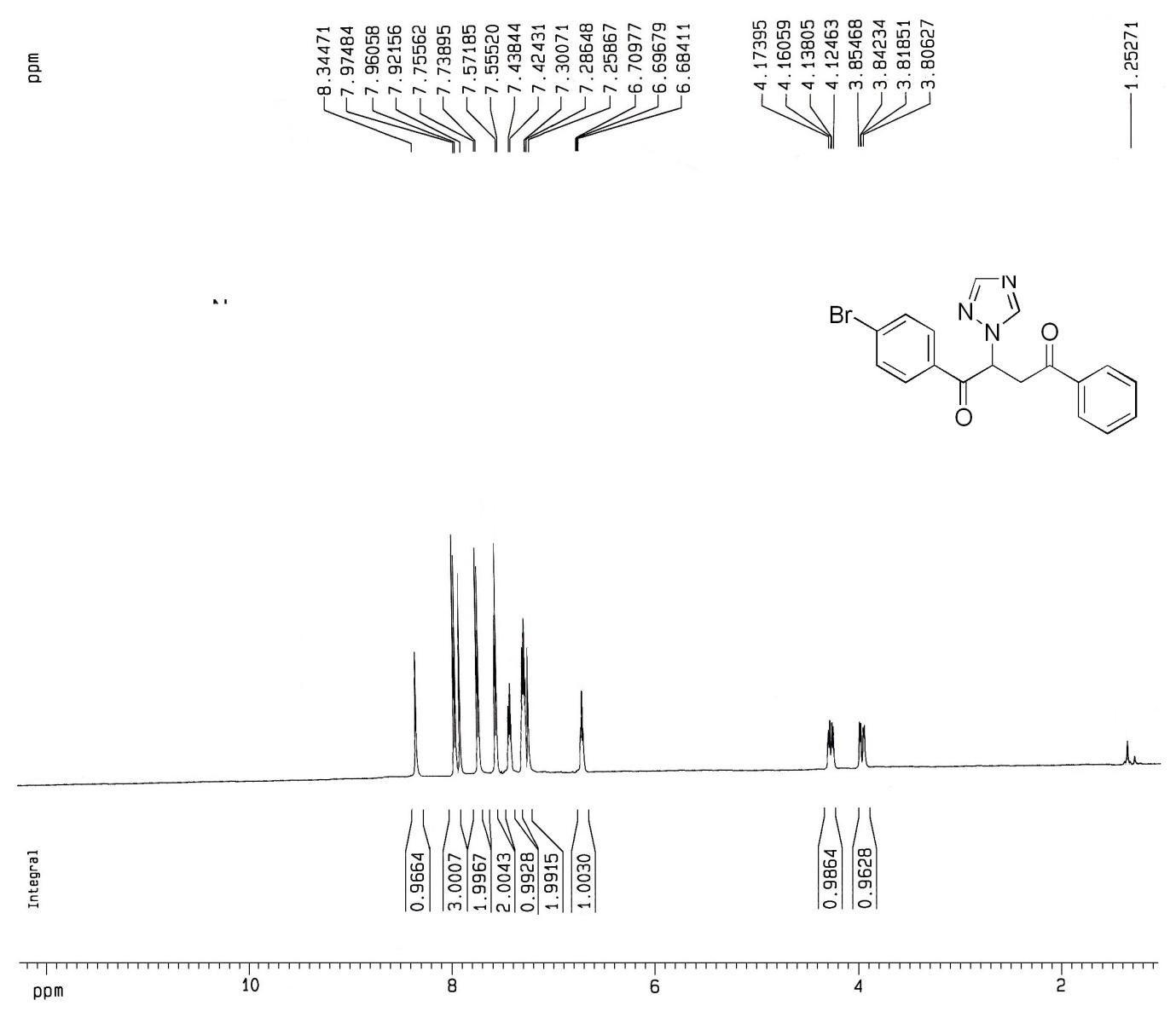


**Figure 33.** ^1^HNMR spectrum of compound **10c.**


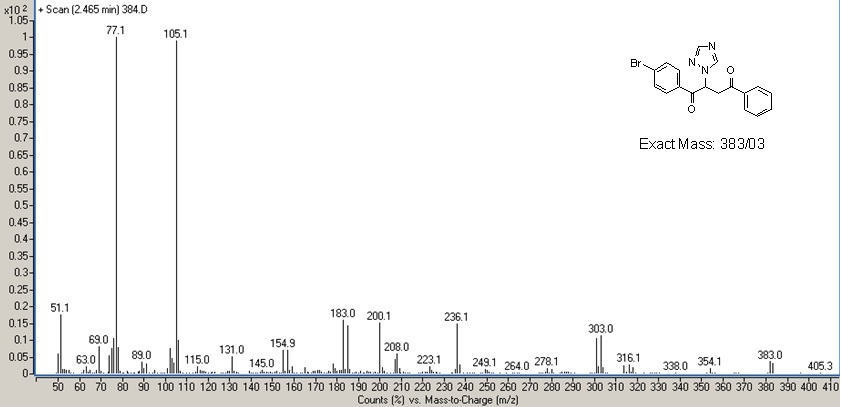


**Figure 34.** Mass spectrum of compound **10c.**


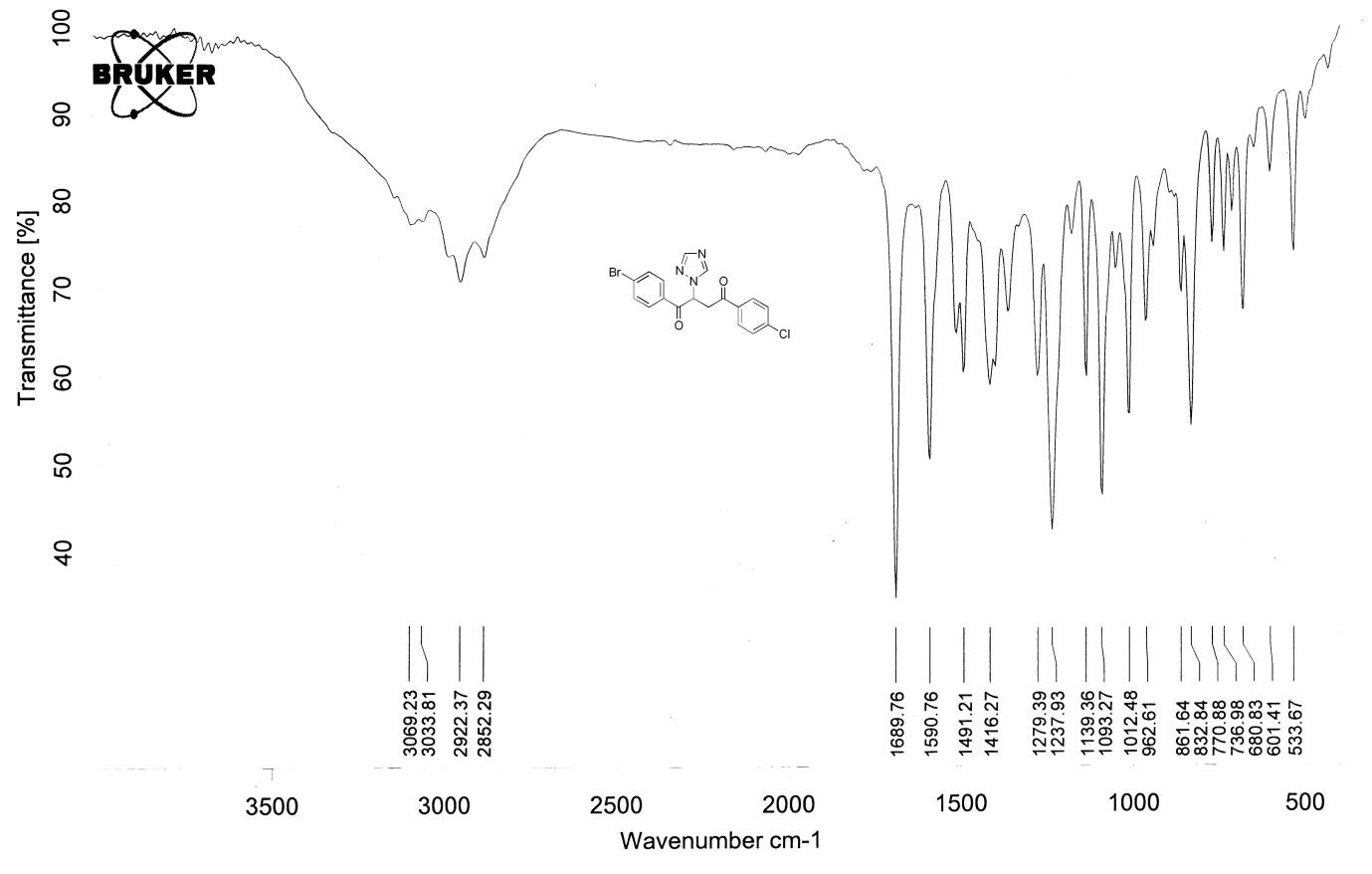


**Figure 35.** IR spectrum of compound **10d.**


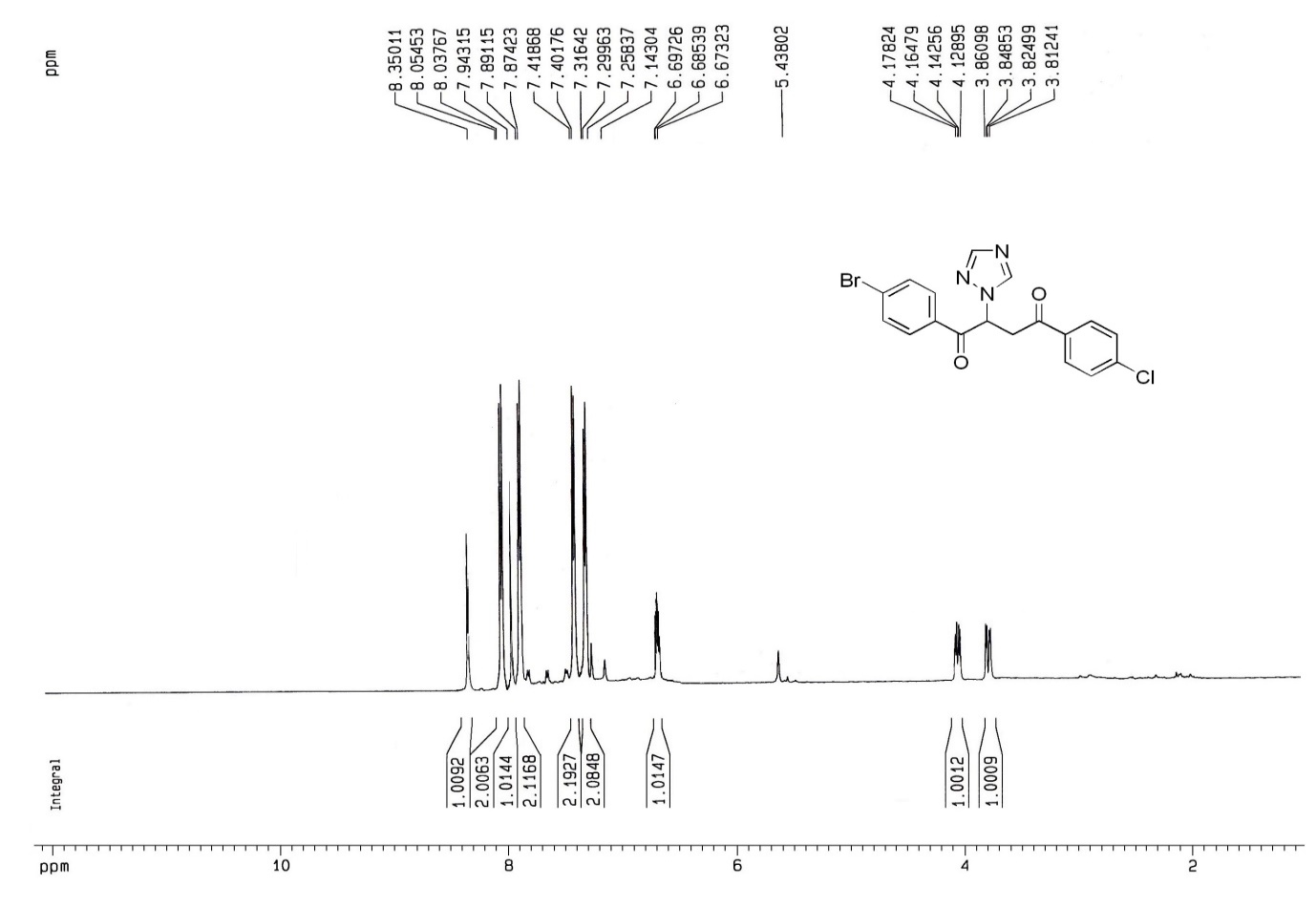


**Figure 36.** ^1^HNMR spectrum of compound **10d.**


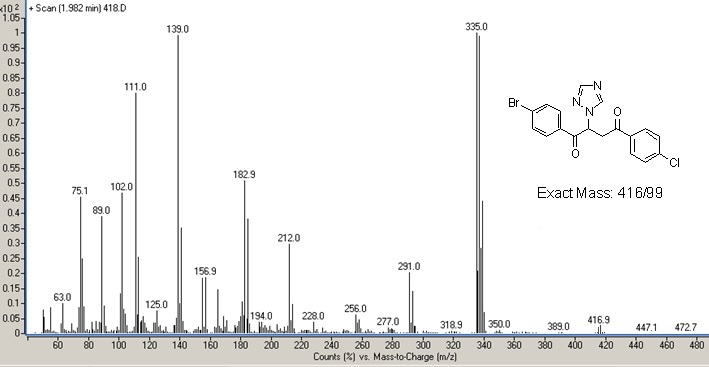


**Figure 37.** Mass spectrum of compound **10d.**


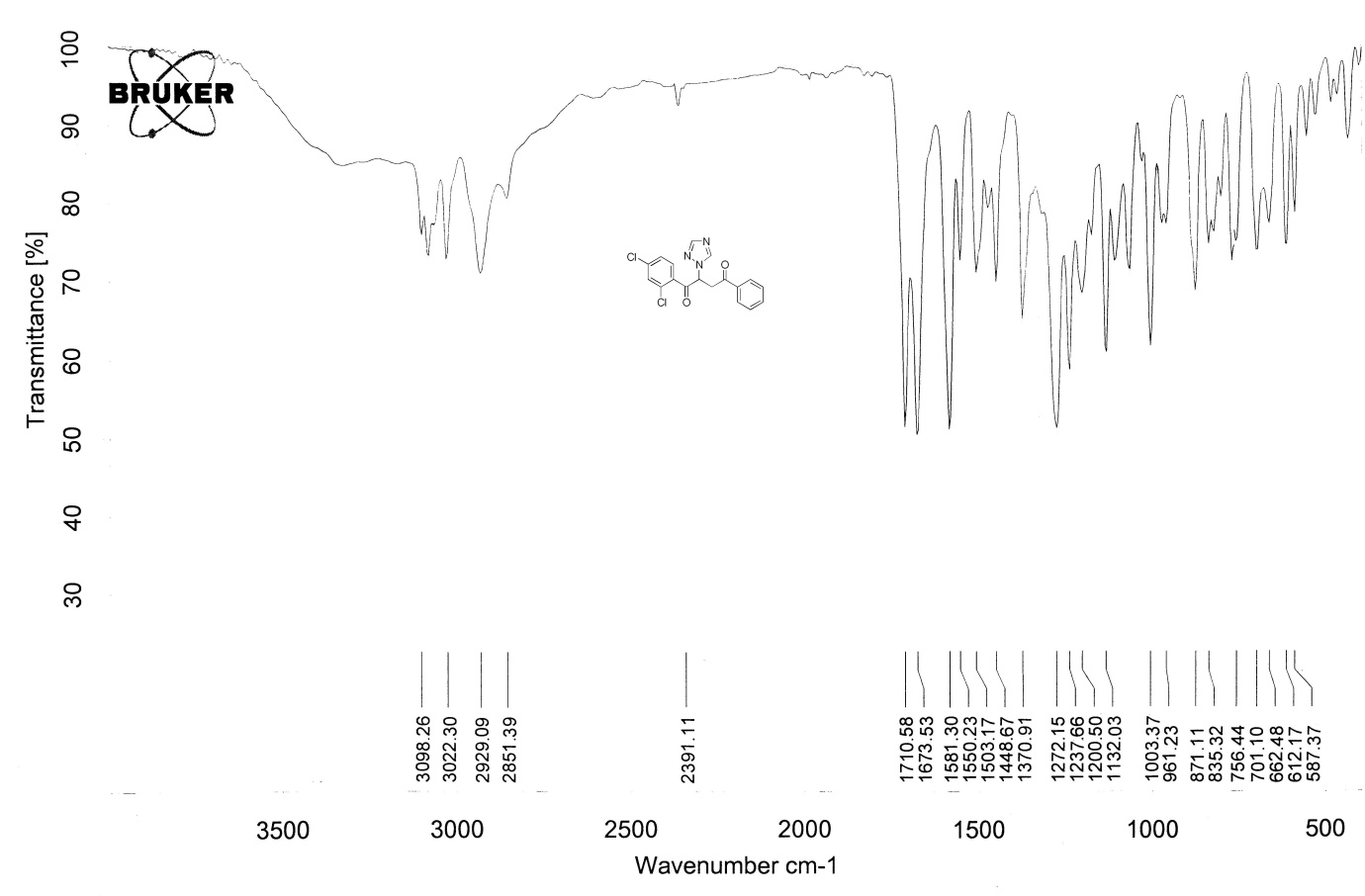


**Figure 38.** IR spectrum of compound **10e.**


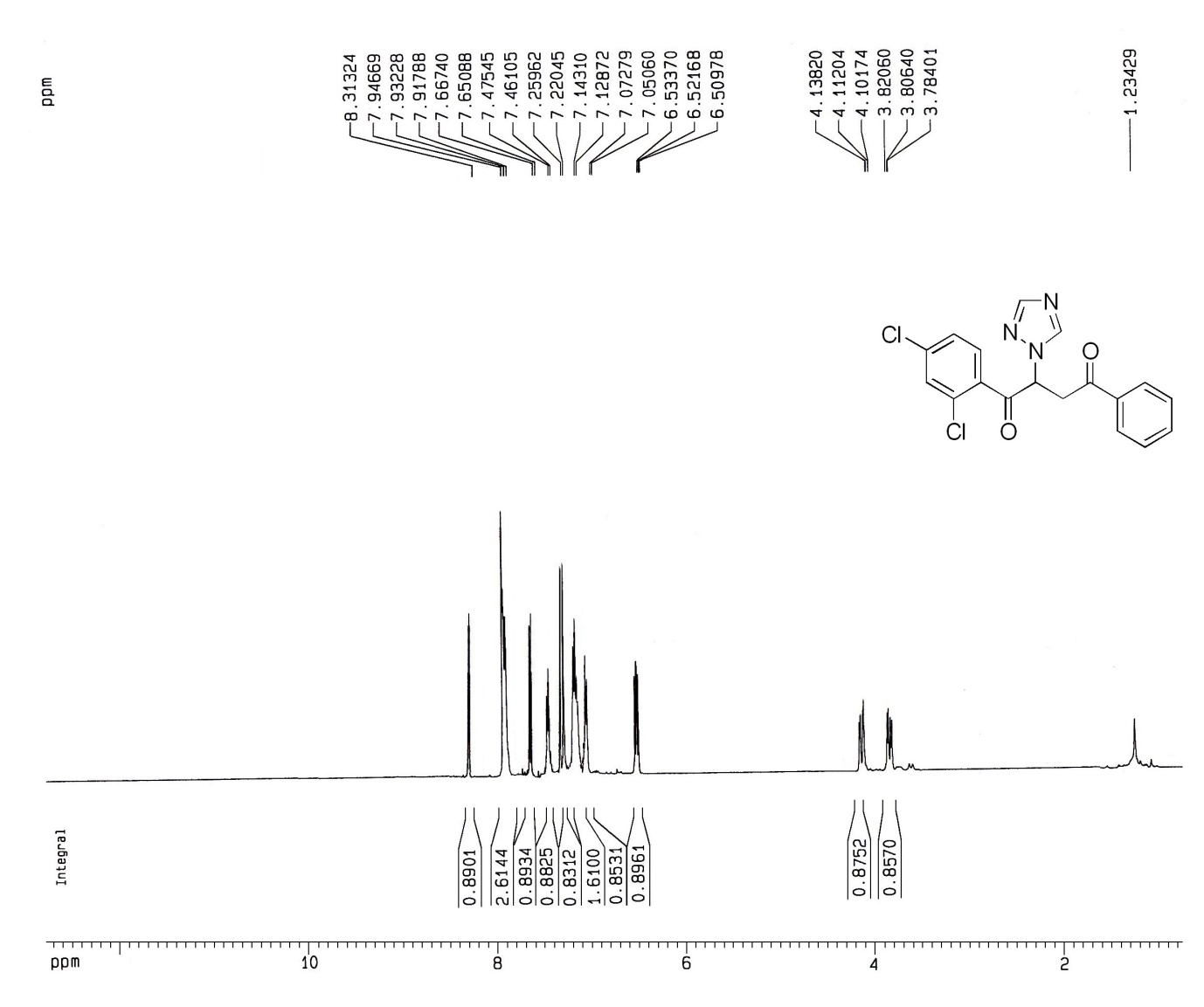


**Figure 39.** 1HNMR spectrum of compound **10e.**


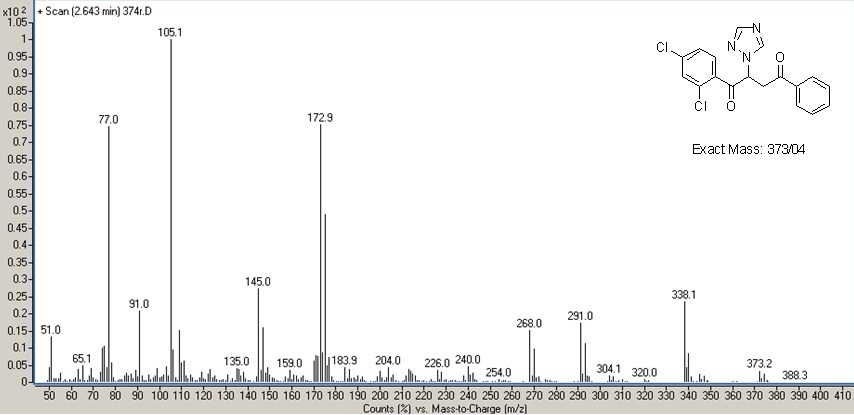


**Figure 40.** Mass spectrum of compound **10e.**


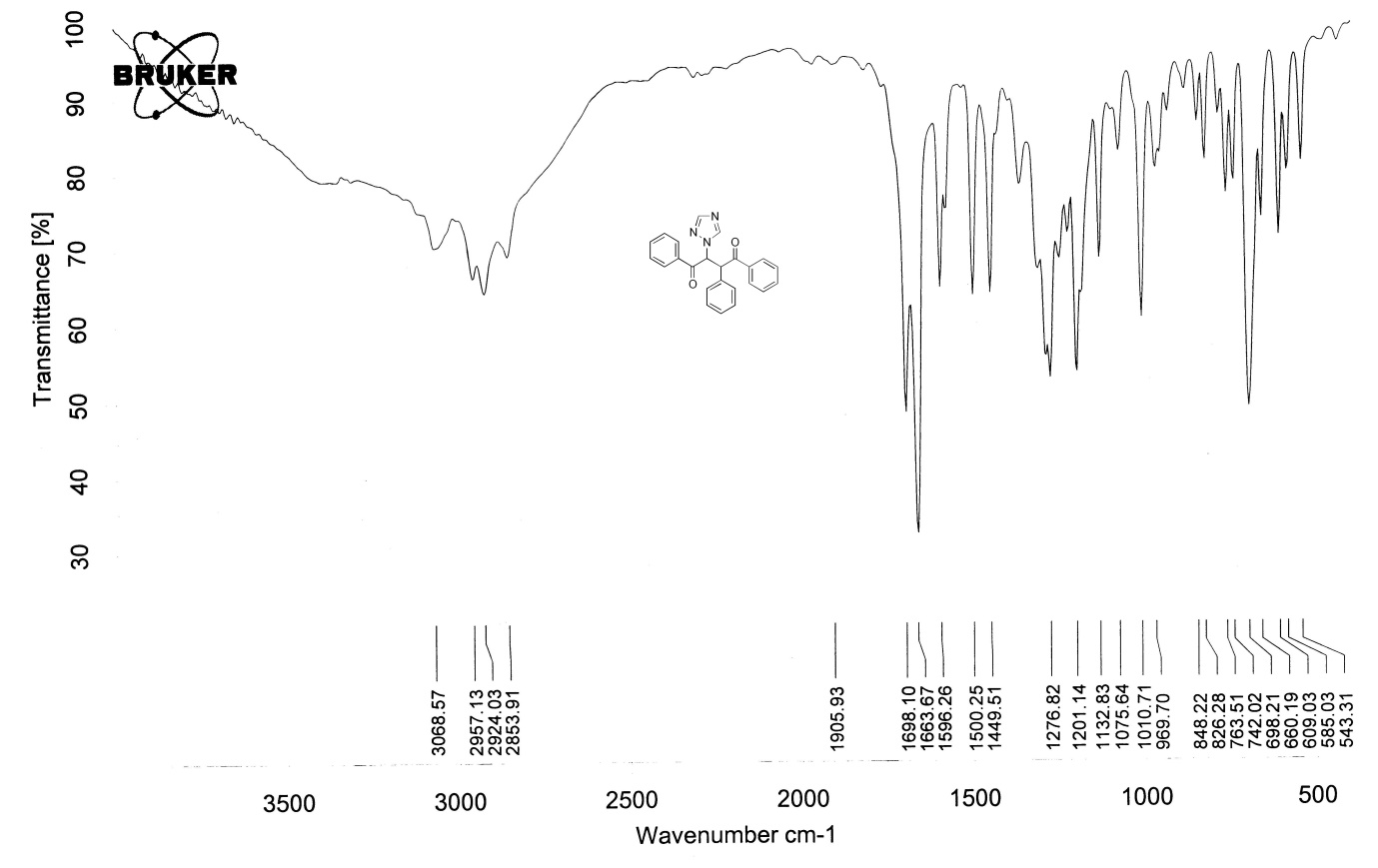


**Figure 41.** IR spectrum of compound **10f.**


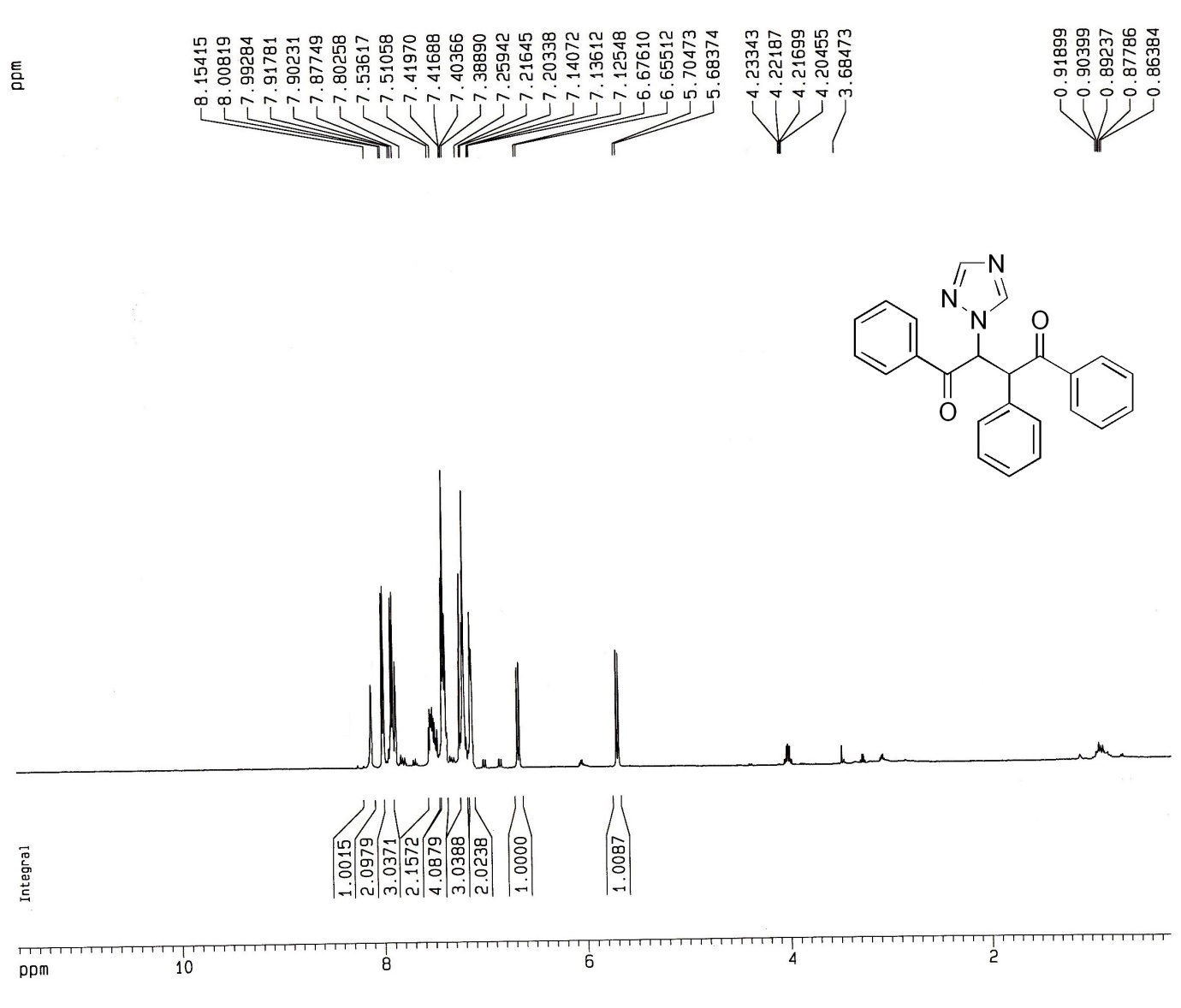


**Figure 42.** ^1^HNMR spectrum of compound **10f.**


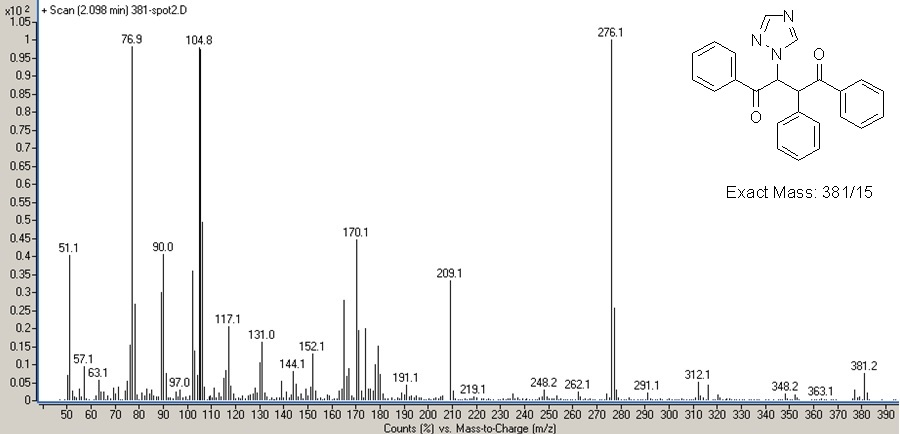


**Figure 43.** Mass spectrum of compound **10f.**


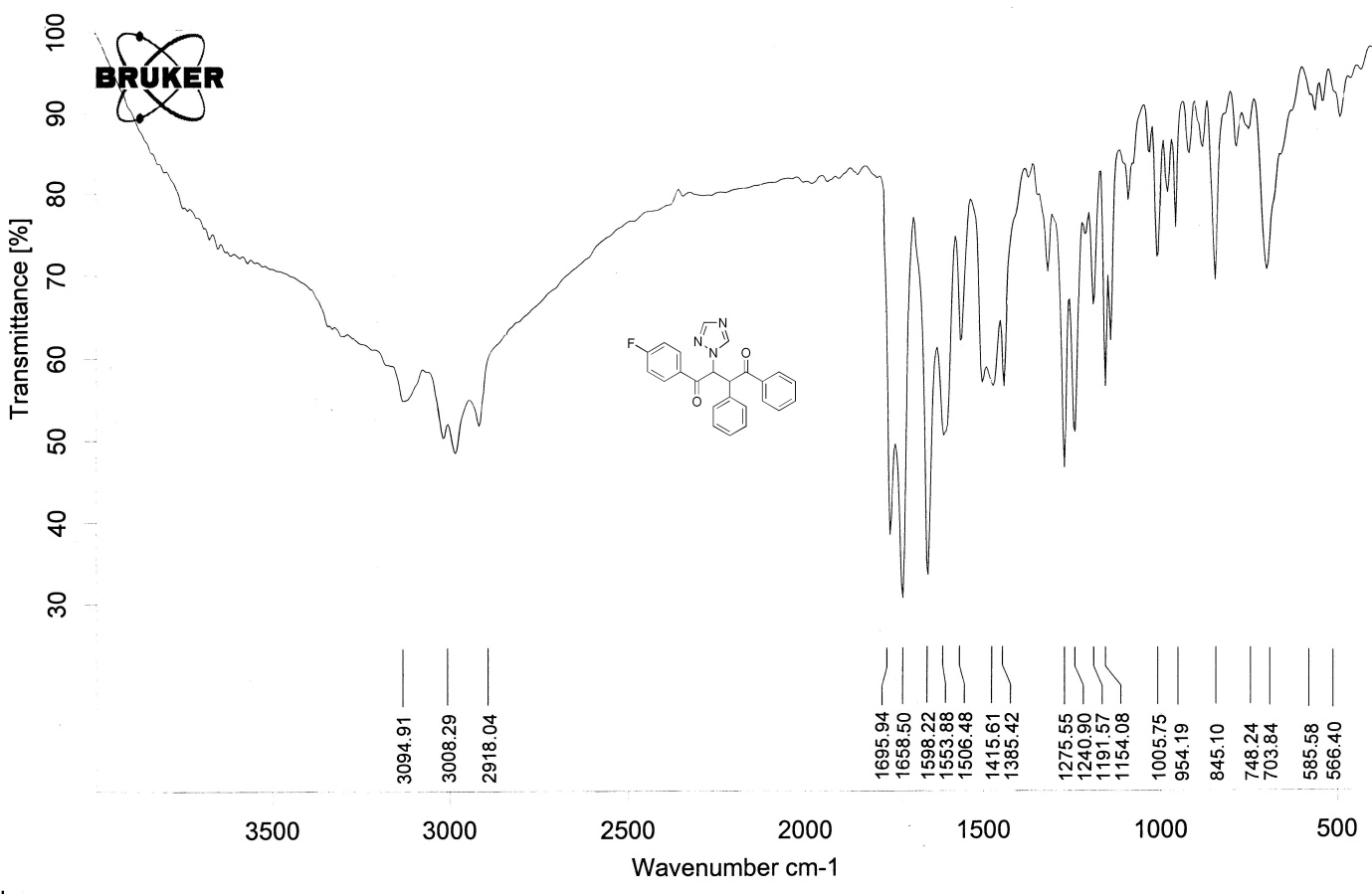


**Figure 44.** IR spectrum of compound **10g.**

**
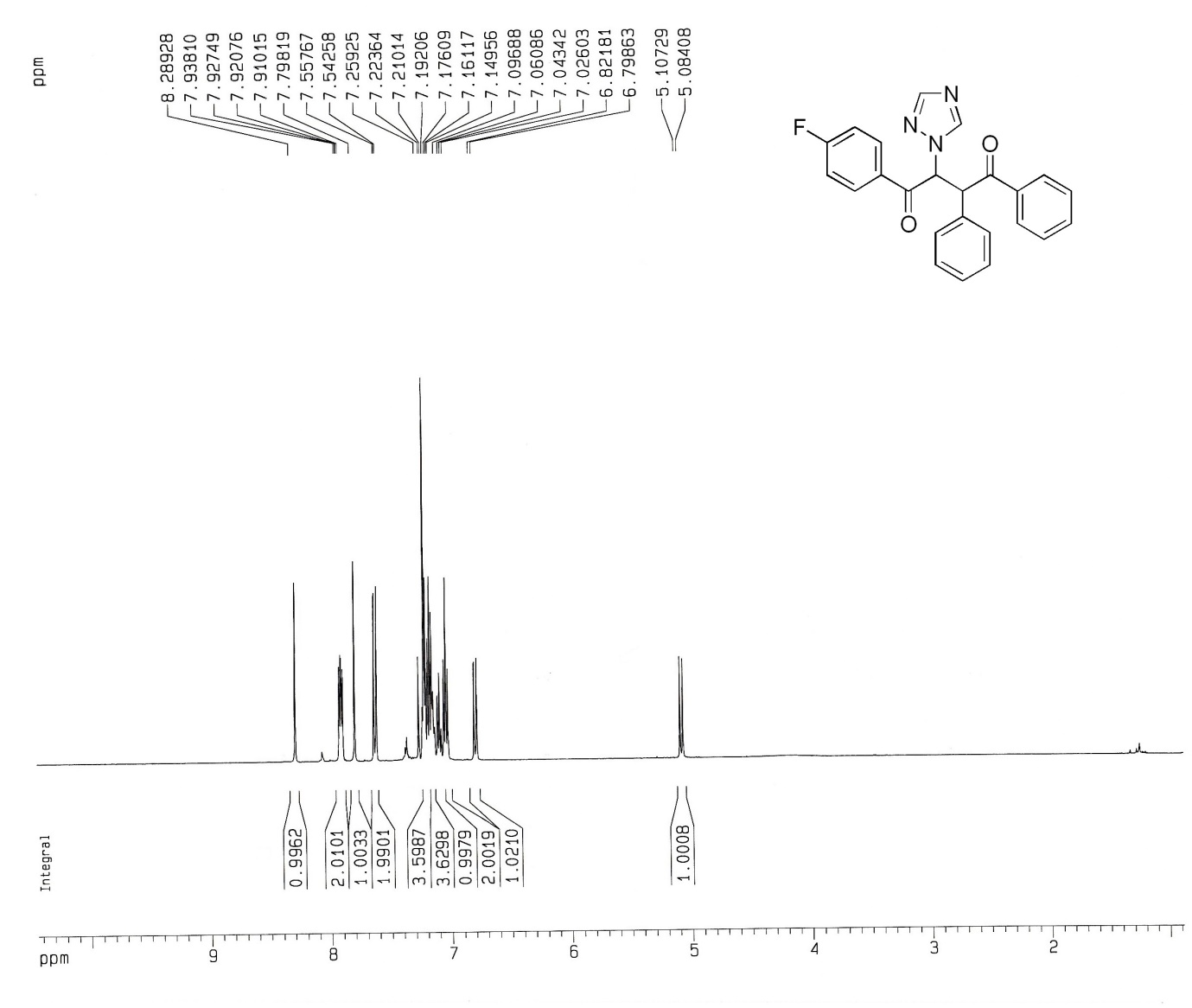
**

**Figure 45.** ^1^HNMR spectrum of compound **10g.**


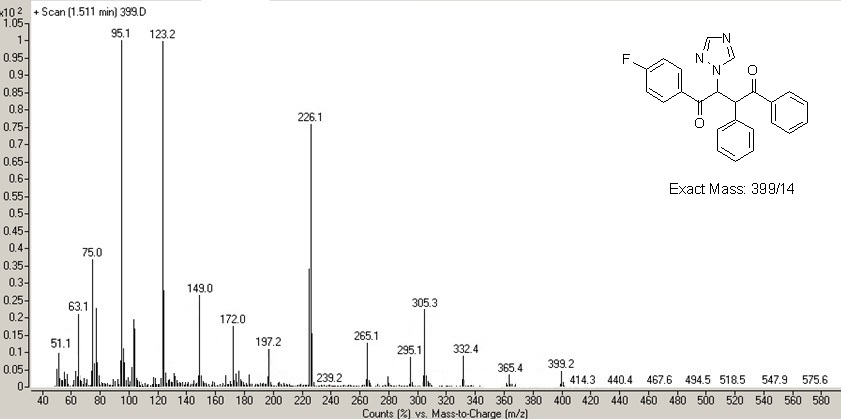


**Figure 46.** Mass spectrum of compound **10g.**


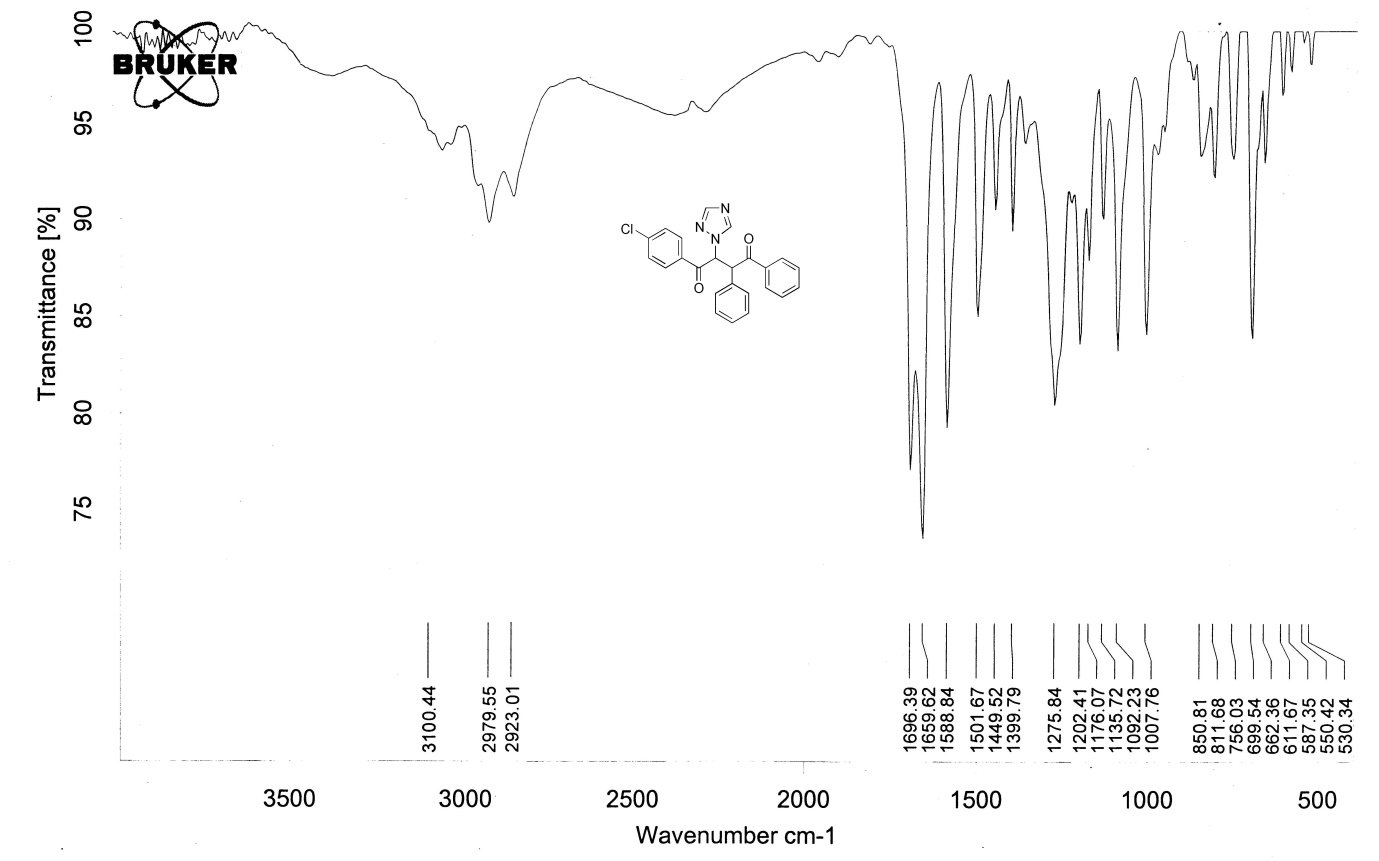


**Figure 47.** IR spectrum of compound **10h.**


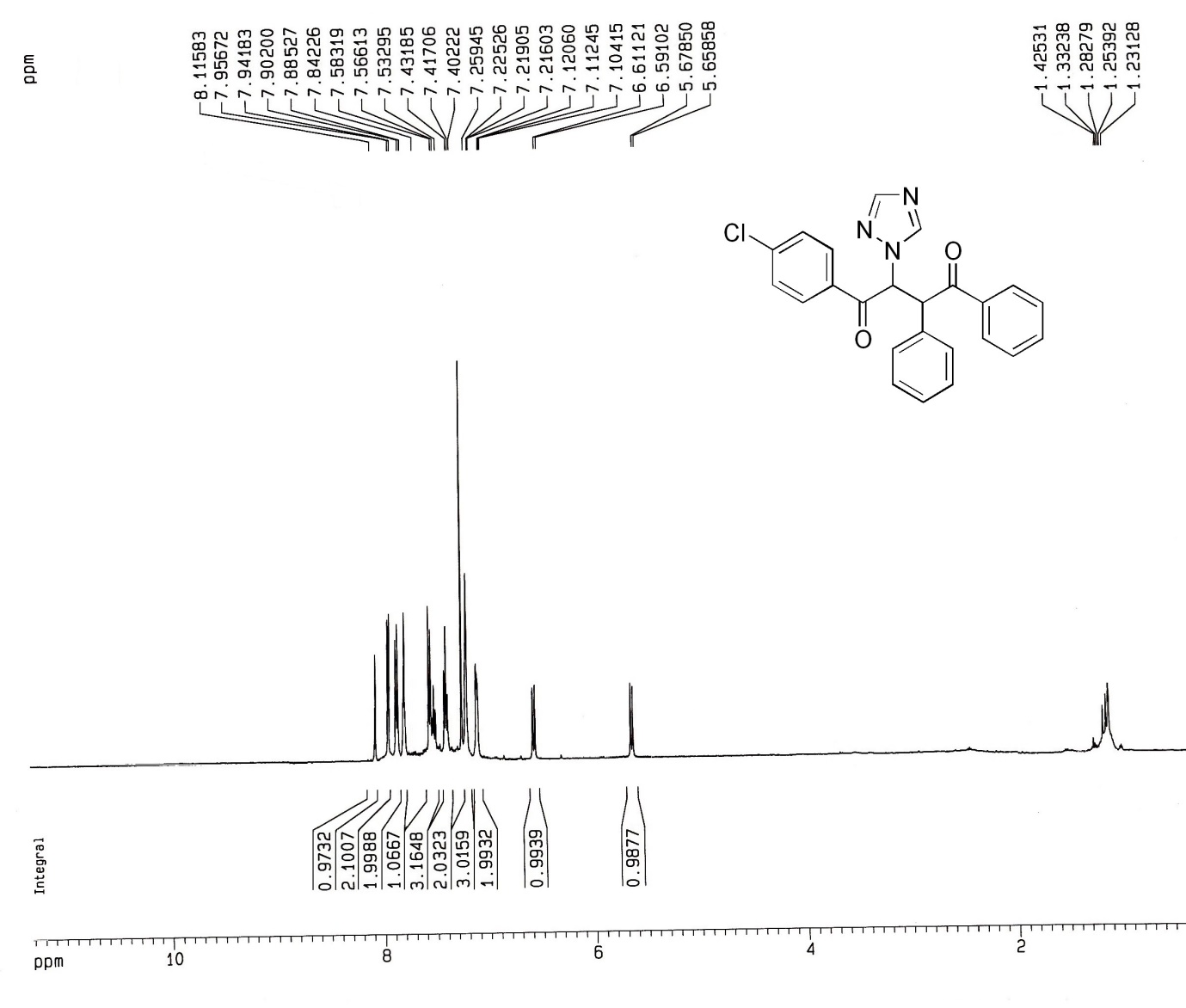


**Figure 48.** ^1^HNMR spectrum of compound **10h.**


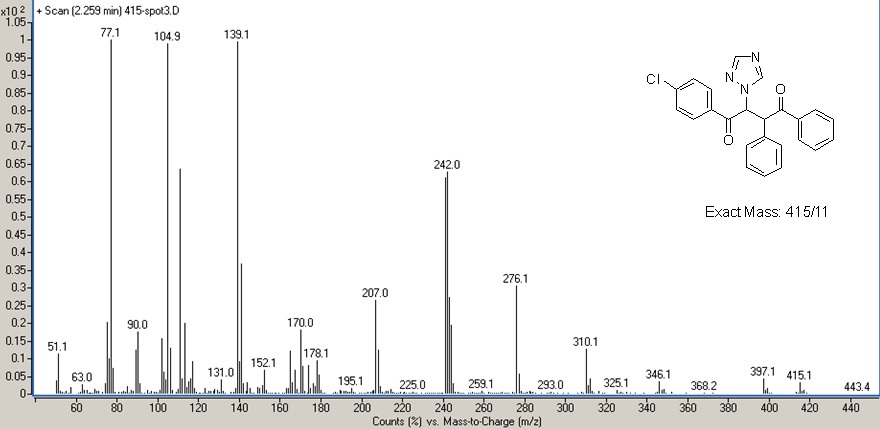


**Figure 49.** Mass spectrum of compound **10h.**


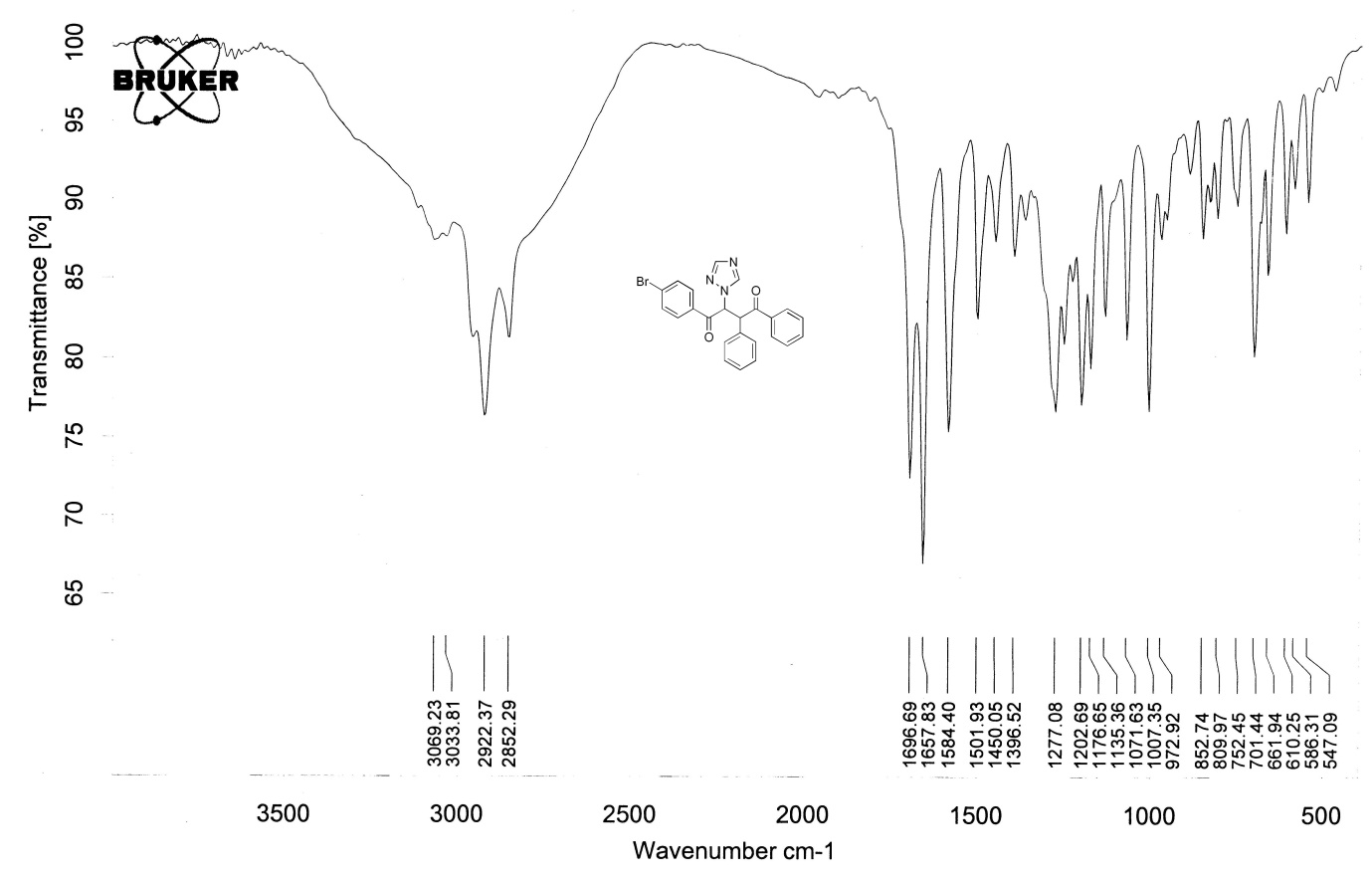


**Figure 50.** IR spectrum of compound **10i.**


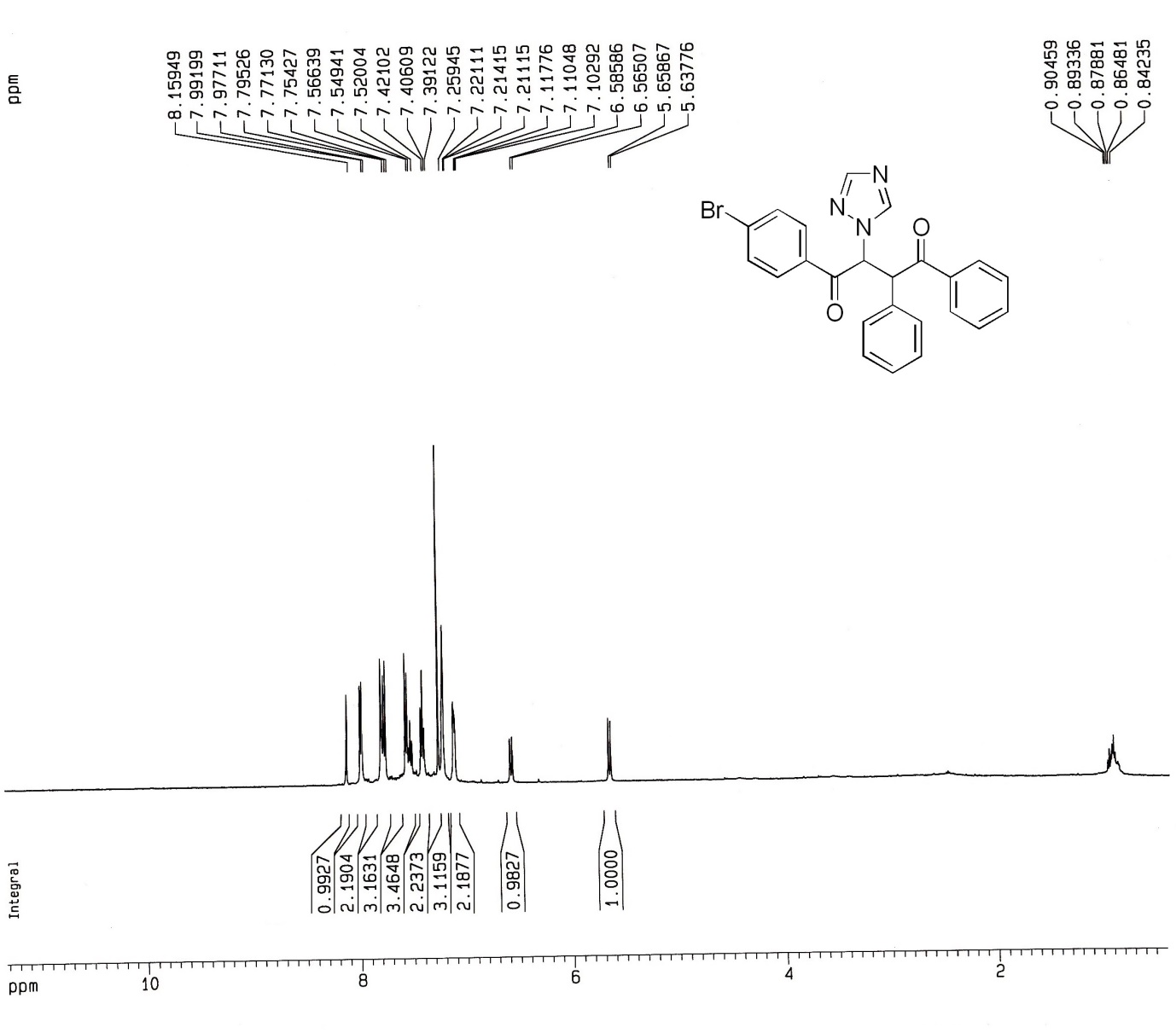


**Figure 51.** ^1^HNMR spectrum of compound **10i.**


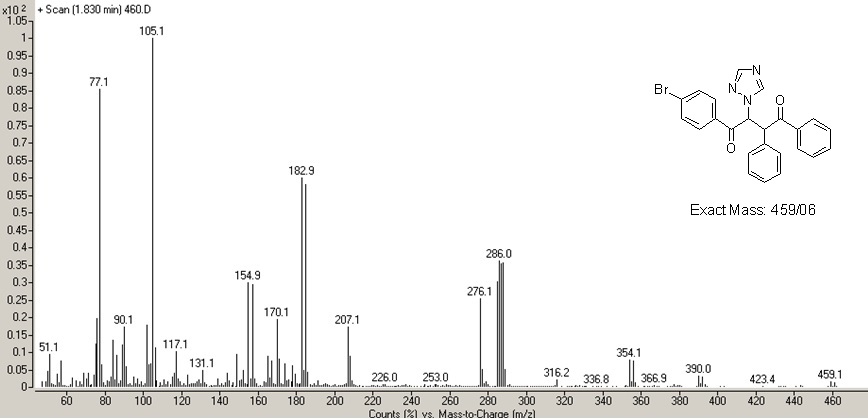


**Figure 52.** Mass spectrum of compound **10i.**


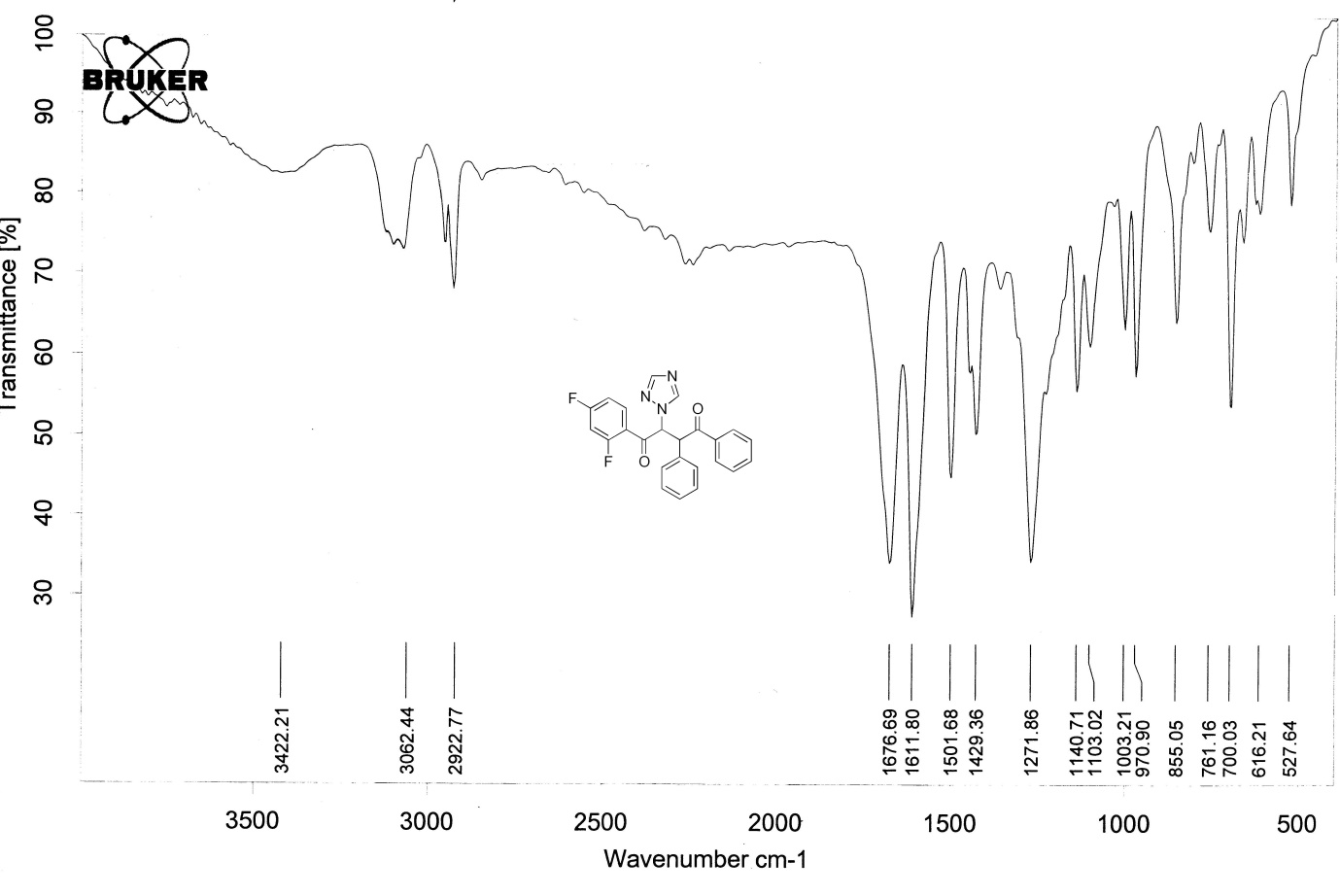


**Figure 53.** IR spectrum of compound **10j.**


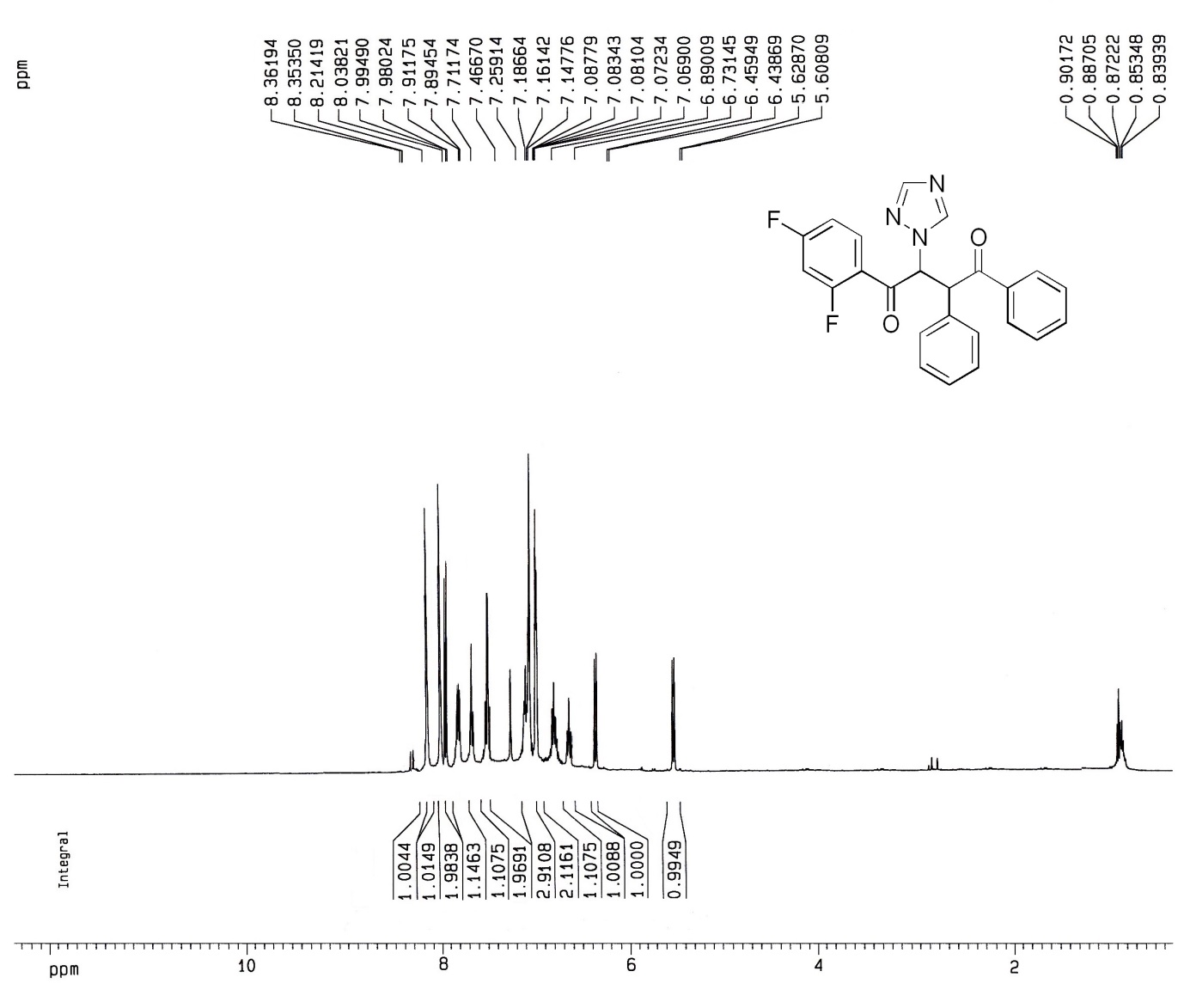


**Figure 54.** ^1^HNMR spectrum of compound **10j.**


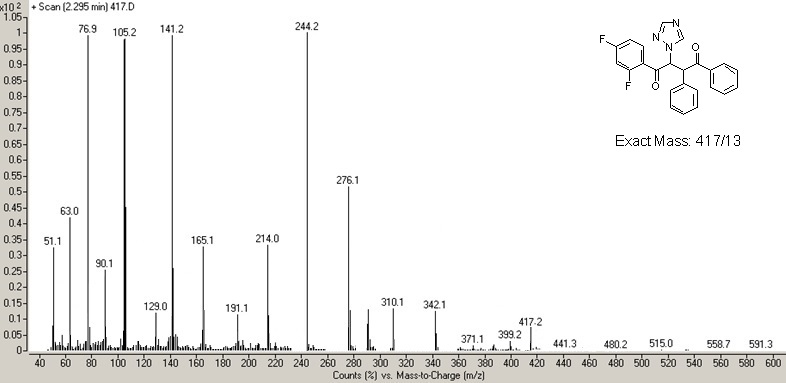


**Figure 55.** Mass spectrum of compound **10j.**


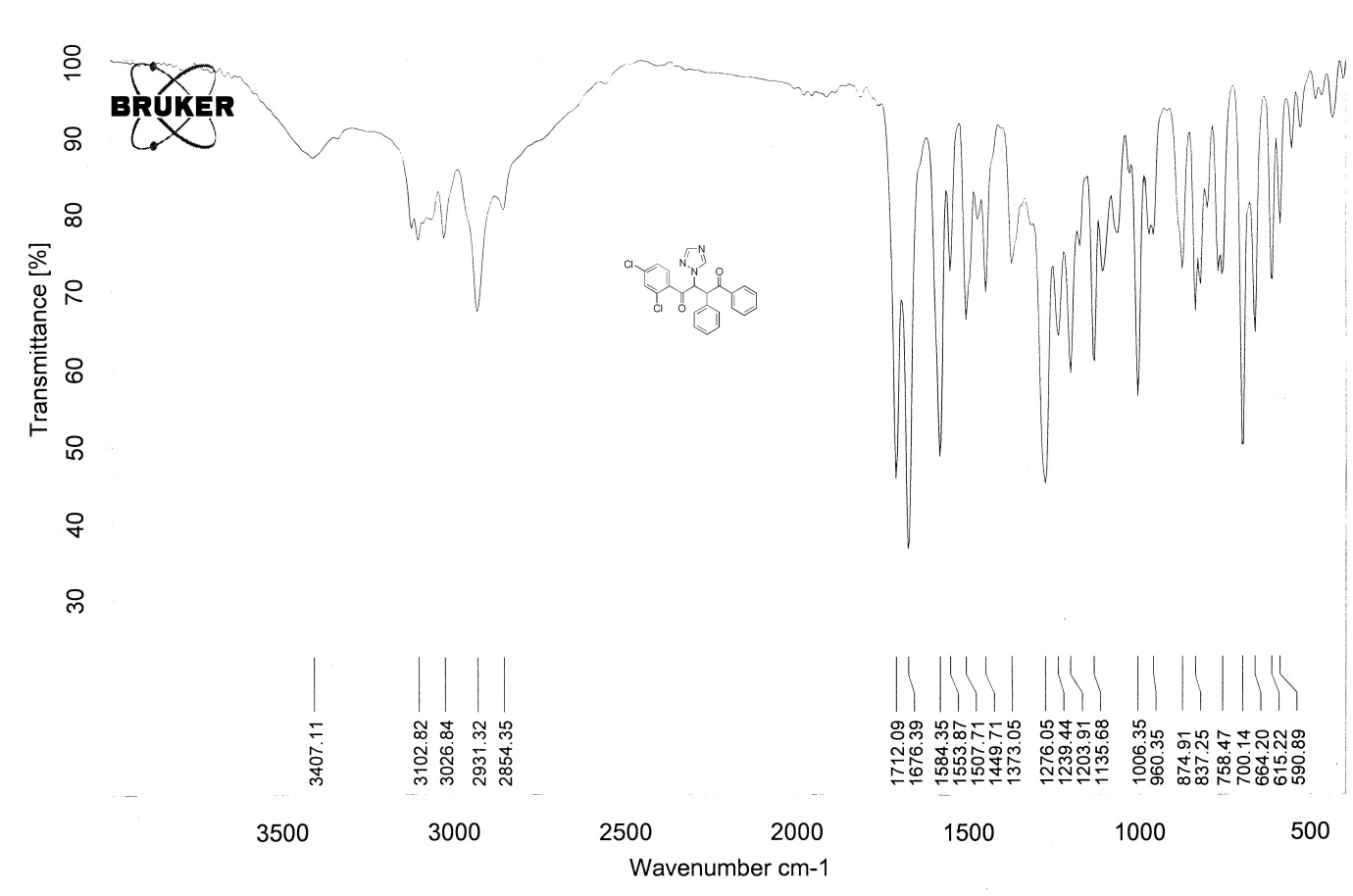


**Figure 56.** IR spectrum of compound **10k.**


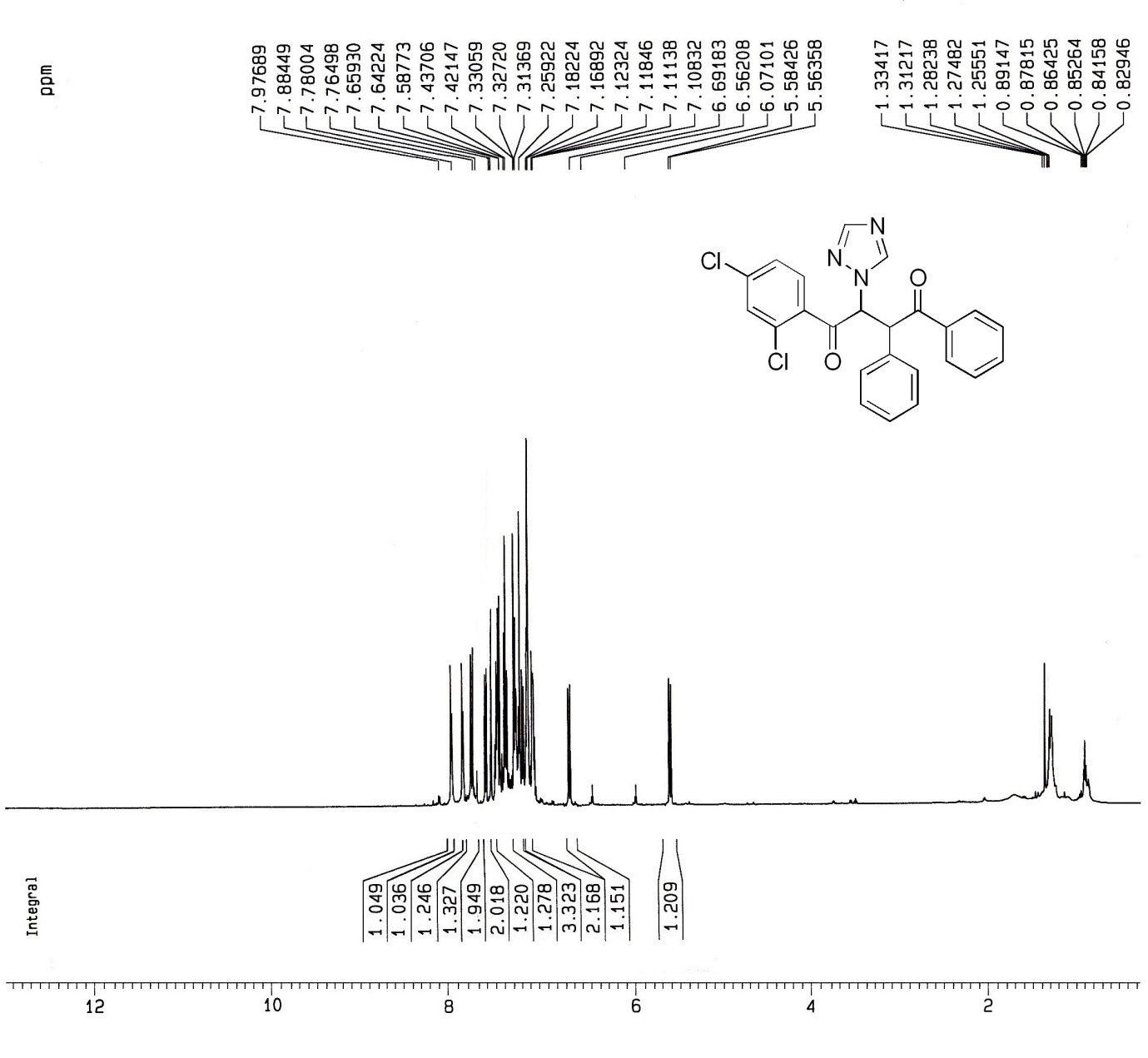


**Figure 57.** ^1^HNMR spectrum of compound **10k.**


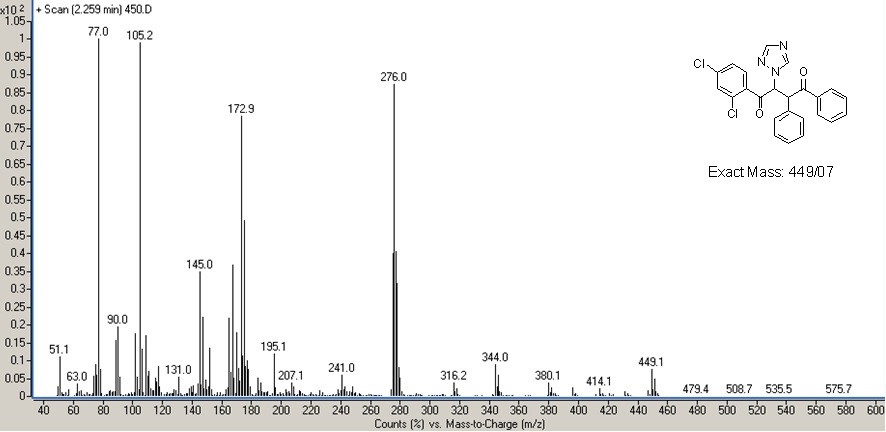


**Figure 58.** Mass spectrum of compound **10k.**

1. *Corresponding author: Zahra Rezaei, Pharmaceutical Sciences Research Center, Shiraz University of Medical Sciences, P.O. Box: 71345-1798 Shiraz, Iran. Email: rezaeiza@sums.ac.ir

   1: These authors contributed equally to this work. [↑](#footnote-ref-1)
